# Factors in AIDS Dementia Complex Trial Design: Results and Lessons from the Abacavir Trial

**DOI:** 10.1371/journal.pctr.0020013

**Published:** 2007-03-30

**Authors:** Bruce J Brew, Mark Halman, Jose Catalan, Ned Sacktor, Richard W Price, Steve Brown, Hamp Atkinson, David B Clifford, David Simpson, Gabriel Torres, Colin Hall, Christopher Power, Karen Marder, Justin C. Mc Arthur, William Symonds, Carmen Romero

**Affiliations:** 1 Department of Neurology and Centre for Immunology, National Centre in HIV Epidemiology and Clinical Research, St. Vincent's Hospital, Darlinghurst, Sydney, Australia; 2 Department of Psychiatry, Saint Michael's Hospital, Toronto, Ontario, Canada; 3 Psychological Medicine Unit, South Kensington and Chelsea Mental Health Centre, Imperial College, London, United Kingdom; 4 HIV Neuroscience Group, Department of Neurology, Johns Hopkins University School of Medicine, Baltimore, Maryland, United States of America; 5 San Francisco General Hospital, Department of Neurology, San Francisco, California, United States of America; 6 University of California Los Angeles, Los Angeles, California, United States of America; 7 HIV Neurobehavioral Research Center, San Diego, California, United States of America; 8 Department of Neurology, Neurologic AIDS Research Consortium, Washington University Medical Center, St. Louis, Missouri, United States of America; 9 Neurophysiology, Mt. Sinai Medical Center, New York, New York, United States of America; 10 St. Vincent's Hospital, New York, New York, United States of America; 11 Department of Neurology, University of North Carolina School of Medicine, Chapel Hill, North Carolina, United States of America; 12 Section of Neurology, Faculty of Medicine, University of Manitoba, Winnipeg, Manitoba, Canada; 13 Neurology, Columbia University, New York, New York, United States of America; 14 Clinical Research, GlaxoWellcome, Research Triangle, North Carolina, United States of America; 15 Clinical Research Department, GlaxoWellcome, Madrid, Spain

## Abstract

**Objectives:**

To determine the efficacy of adding abacavir (Ziagen, ABC) to optimal stable background antiretroviral therapy (SBG) to AIDS dementia complex (ADC) patients and address trial design.

**Design:**

Phase III randomized, double-blind placebo-controlled trial.

**Setting:**

Tertiary outpatient clinics.

**Participants:**

ADC patients on SBG for ≥8 wk.

**Interventions:**

Participants were randomized to ABC or matched placebo for 12 wk.

**Outcome Measures:**

The primary outcome measure was the change in the summary neuropsychological Z score (NPZ). Secondary measures were HIV RNA and the immune activation markers β-2 microglobulin, soluble tumor necrosis factor (TNF) receptor 2, and quinolinic acid.

**Results:**

105 participants were enrolled. The median change in NPZ at week 12 was +0.76 for the ABC + SBG and +0.63 for the SBG groups (*p* = 0.735). The lack of efficacy was unlikely related to possible limited antiviral efficacy of ABC: at week 12 more ABC than placebo participants had plasma HIV RNA ≤400 copies/mL (*p* = 0.002). There were, however, other factors. Two thirds of patients were subsequently found to have had baseline resistance to ABC. Second, there was an unanticipated beneficial effect of SBG that extended beyond 8 wk to 5 mo, thereby rendering some of the patients at baseline unstable. Third, there was an unexpectedly large variability in neuropsychological performance that underpowered the study. Fourth, there was a relative lack of activity of ADC: 56% of all patients had baseline cerebrospinal fluid (CSF) HIV-1 RNA <100 copies/mL and 83% had CSF β-2 microglobulin <3 nmol/L at baseline.

**Conclusions:**

The addition of ABC to SBG for ADC patients was not efficacious, possibly because of the inefficacy of ABC per se, baseline drug resistance, prolonged benefit from existing therapy, difficulties with sample size calculations, and lack of disease activity. Assessment of these trial design factors is critical in the design of future ADC trials.

## Editorial Commentary

**Background:** AIDS dementia complex (ADC) was first identified early in the HIV epidemic and at that time affected a substantial proportion of patients with AIDS. Patients with ADC experience dementia as well as disordered behavior and problems with movement and balance. ADC is now much less common in locations where patients have access to HAART (highly active antiretroviral therapy), consisting of combinations of several drugs that attack the virus at different stages of its life cycle. At present, however, there are no generally accepted guidelines for the best treatment of people with ADC. It has been thought for some time that the best treatment regimens for people with ADC would include drugs that cross the barrier between blood and brain well. Shortly following the introduction of HAART, a trial was carried out to find out whether adding one particular drug, abacavir, to existing combinations of drugs would be beneficial in people with ADC. This drug is known to cross the barrier between the blood and brain. The trial enrolled HIV-positive individuals with mild to moderate ADC and who were already receiving antiretroviral drug treatment. 105 participants were assigned at random to receive either high-dose abacavir or a placebo, in addition to their existing therapy. The primary outcome of the trial was a summary of performance on a set of different tests, designed to evaluate cognitive, behavior, and movement skills, at 12 weeks. Other outcomes included levels of HIV RNA (viral load) in fluid around the brain, as well as other neurological evaluations, and the level of HIV RNA and CD4^+^ T cells (the cells infected by HIV) in blood.

**What this trial shows:** When comparing the change in performance scores for individuals randomized to either abacavir or placebo, the results showed an improvement in scores for both groups, but no significant difference in improvement between the two groups. Similarly, the levels of HIV RNA in the cerebrospinal fluid did not differ between the two groups being compared, and other neurological tests did not show any differences between the two groups. However, at 12 weeks, patients receiving abacavir were more likely to have low levels of HIV RNA in their blood, suggesting that abacavir was active against the virus, but this did not translate into an additional improvement of these patients' dementia. The overall rates of adverse events were roughly comparable between the two groups in the trial, although participants receiving abacavir were more likely to experience certain types of events, such as nausea.

**Strengths and limitations:** The trial was appropriately randomized and controlled, using central telephone procedures for randomizing participants and subsequent blinding of patients and trial investigators. These procedures help minimize the chance of bias in assigning participants to the different arms as well as in the subsequent performance of individuals within the trial and the assessment of their outcomes. Limitations in the study design have been identified. One limitation is that individuals enrolled into the trial may not in fact have been receiving their existing HAART regimen for long enough to experience its optimal effect, and therefore the improvement seen in both groups could have resulted from an ongoing response to their existing regimen. It is also possible that patients improved in their test scores over the course of the trial simply because they became more familiar with the tests and not because their condition improved. This is a problem in all such trials that try to improve mental function. Finally, a limitation may have been the inclusion of patients who did not have active disease leading to worsening dementia.

**Contribution to the evidence:** The findings from this trial suggest that adding high-dose abacavir to existing HAART is not beneficial for patients with ADC. However, the trial provides several insights into the way that future studies of this type can be done, and which typically pose a number of challenging design problems. In particular, sensitive markers are needed that will allow researchers to monitor progression of ADC and patients' response to therapy.

## INTRODUCTION

AIDS dementia complex (ADC) is a relatively common complication of advanced HIV disease, occurring in approximately 20% of patients in the era before highly active antiretroviral therapy (HAART) [1]. ADC is characterized by a complex of cognitive, motor, and behavioral abnormalities [2]. While the incidence in the HAART era has halved relative to pre-HAART, the prevalence has approximately doubled, because of the life-extending effect of HAART [3]. Zidovudine (ZDV) is the only antiretroviral drug whose efficacy in ADC is supported by the results of a randomized, placebo-controlled trial [4]. In addition, the efficacy of ZDV is supported by anecdotal and observational studies [5,6], as well as evidence of significant declines in the concentrations of cerebrospinal fluid (CSF) immune activation markers such as β-2 microglobulin and quinolinic acid, both of which are closely correlated with the severity of ADC and its response to therapy [7,8]. A number of antiretroviral drugs may be effective in the treatment of ADC, based upon reduction of HIV-1 RNA in the CSF, as seen with stavudine (d4T), lamivudine (3TC) [9], and indinavir [10]; the measurement of potentially efficacious drug concentrations in the CSF, as observed with nevirapine and efavirenz [11,12]; and efficacy in in vitro models (d4T, 3TC, and efavirenz) [13]. Although the effect of combining these and other antiretroviral drugs within HAART on clinical and neuropsychological performance has not been systematically studied, there is increasing evidence suggesting a beneficial impact on ADC [14–17].

For an antiretroviral therapy (ART) to be effective in ADC, theoretically it should have potent antiviral activity, ability to penetrate the brain at efficacious concentrations, and activity in macrophages and microglia, the primary targets of HIV infection within the brain. While the need for adequate brain penetration is controversial, studies addressing this issue thus far have been incomplete. There has been variability in the number of drugs that have been assessed in the regimen (at times one drug and at other times three), in the comprehensiveness of neuropsychological assessments, in the level of impairment of the patients, and in the potential for drug interactions. Moreover, there has been no head-to-head comparison of a brain-penetrating three-drug regimen versus one that is not penetrating. In a recent study, we have found that while *overall* there does not appear to be any benefit from a brain-penetrating regimen in neurologically asymptomatic patients, there is neuropsychological benefit in a *subset* of patients with significant dysfunction [18].

Abacavir (Ziagen, ABC) is metabolized to a novel 2′ deoxyguanosine analog nucleoside reverse transcriptase inhibitor (NRTI) that fulfils these criteria. The compound has potent antiviral efficacy, can cross the blood brain barrier to an extent similar to ZDV, and has good antiviral activity in macrophages in vitro [19] and microglia [20]. Furthermore, while ZDV is active against de novo infection of macrophages, but not against *chronic* infection in resting monocyte lineage cells, ABC is active against both [21]. Consequently, a controlled study was conducted to determine the efficacy of ABC as part of combination therapy in ADC. An ABC dose of 600 mg twice a day (double the standard dose) was selected in an attempt to maximize the central nervous system ABC concentrations while providing an acceptable level of safety [22]. Results for the 12-wk randomized phase of the study, presented below, did not show any benefit for the addition of ABC. The trial details are analyzed here to determine the reasons for this lack of efficacy.

## METHODS

### Participants

Thirteen sites in Australia, Canada, the United Kingdom, and the United States participated. Each site was a tertiary referral outpatient clinic. Confirmed HIV-1 seropositive male or female participants, aged 18 to 65 y, diagnosed with Stage 1 or 2 (mild to moderate) ADC [23] and stable on optimal ART (SBG) for a minimum of 8 wk prior to study were enrolled. Participants had to be impaired by at least 1.5 standard deviations (SDs) below normal in at least two neuropsychological domains from the chosen test battery (see below). Participants with evidence of confounding neurological disease or presenting with other central nervous system opportunistic infections or neoplasms were excluded. No restriction on previous ART was imposed**.**

### Interventions

This was a phase III randomized, placebo-controlled, double-blind study. Ethics approval was obtained from each site. Informed consent was obtained from each participant or from their legal guardian. All clinical investigations were conducted according to the principles expressed in the Declaration of Helsinki.

Participants received 600 mg of ABC or matched placebo twice a day in addition to their current SBG for the 12 wk of the study; changes in drugs or drug dosages within the SBG were not permitted during this time. Participants who experienced progression or severe antiretroviral drug toxicity not related to ABC during the study could receive open label ABC, 600 mg twice a day, and change the SBG as appropriate if a minimum of 6 wk on study had been completed. Participants were defined as having clinically progressed if their ADC clinical status deteriorated by at least one stage [23] with this endpoint validated by a core committee.

### Objectives

The objective of the trial was to determine the efficacy of adding ABC to SBG to patients with ADC.

### Outcome Measures

The primary outcome measure was the change in the summary neuropsychological Z score (NPZ). Secondary measures were CSF HIV RNA and the immune activation markers β-2 microglobulin and soluble tumor necrosis factor (TNF) receptor 2 and quinolinic acid. Ancillary measures were semi-quantitative neurological evaluations, plasma HIV RNA, CD4^+^ cell count, mutations in the reverse transcriptase (RT) gene, and pharmacokinetic analyses.

#### Neuropsychological performance.

Neuropsychological performance was measured by a test battery comprised of the Rey Auditory Verbal Learning Test (RAVLT) Trials I–V (total words across five trials); Delayed Recall, Grooved Pegboard (dominant and nondominant hand); Trail Making B, Symbol Digit, Cal Cap Reaction Time (choice and sequential RT); and Verbal Fluency (word generation on FAS test). These tests are widely used in the United States, the United Kingdom, and Australia and have been validated in these populations. Z scores were constructed using the Multicentre-AIDS Cohort Study (MACS) reference data corrected for age and education [24].

#### CSF HIV-1 RNA.

Collection of CSF samples was compulsory at day 7, but voluntary at weeks 6 and 12. CSF HIV-1 RNA was stored with appropriate precautions at −70 °C and later batch-extracted, amplified, hybridized, and detected using NASBA HIV-1 QT technology (Organon Teknika) with a detection limit of 100 copies/mL.

#### CSF markers of immune activation.

β-2 microglobulin and soluble TNF receptor 2 (sTNF-R2) [25] were measured using commercially available kits. Quinolinic acid was measured using gas chromatography/mass spectrometry.

### Other Measures

####  Neurological evaluation.

Neurological evaluations were performed using the AIDS Clinical Trials Group (ACTG) assessments, pre-study (day 14), weeks 6 and 12. Cognitive function, motor function, behavior/mood, mental status, level of consciousness, adapted mini-mental status exam, response slowing, cranial nerves, ocular motility, facial expression, limb strength, coordination, reflexes, and neuropathy were examined. The total neurological subscore was calculated (from a previously validated formula [B. J. Brew and R. W. Price, personal communication] that gave certain weightings to the latter aspects of the neurological evaluation) and used as an objective tool to confirm the participants' ADC stage (see Text S1). The neurological subscores were as follows: ADC Stage 1 (4–6), Stage 2 (7–9), and Stage 3 (10–12). Neuropathy was defined as the presence of symptoms of numbness and/or pain in the feet with absent or depressed ankle reflexes and diminished perception of pain, temperature, and vibration in the feet.

#### Plasma HIV-1 RNA.

Plasma HIV-1 RNA was assessed pre-study (day 7), at weeks 2, 4, 6, 8, and 12. Quantitation of plasma HIV-1 RNA was carried out using the RNA PCR technique developed by Roche Molecular Systems (http://www.roche-diagnostics.com) (Amplicor HIV-1 MONITOR v1.0, detection limit 400 copies/mL).

#### CD4^+^ cell count.

CD4^+^ cell count was assessed by flow cytometry at day 0, weeks 2, 4, 6, 8, and 12.

#### Mutations in the HIV-1 RT gene in plasma and CSF.

The HIV-1 coding region was amplified using the rTth XL RT-PCR kit (Perkin-Elmer). Purified cDNA was sequenced using the PRISM FS dye terminator cycle sequencing kit (Applied Biosystems, http://www.appliedbiosystems.com) and resolved on an ABI 373 DNA sequencer. Data were aligned and analyzed using the Sequencher program. Where mixed viral populations were present, a ratio of mutant to wild-type electropherogram peak size greater than 70% was designated as mutant.

#### Pharmacokinetic measures.

Voluntary CSF samples (0.5 mL) were collected from a subset of participants at weeks 6 and 12 at various times after dosing. ABC concentrations were determined by a validated analytical method. Results are reported in four pre-defined time intervals.

#### Safety and tolerability.

Safety and tolerability were evaluated by assessing clinical adverse events and clinical laboratory values. Adverse events and toxicities were graded using the ACTG grading scale.

### Sample Size

We calculated a priori that a sample size of 45 participants per treatment group would be required to provide 90% power to detect a difference between treatment groups of 0.6 in the change from baseline of summary NPZ at week 12. This sample size assumes α = 0.05, SD = 0.75, and a dropout rate of 20%–25%.

### Randomization

Randomization was stratified by ZDV use (use versus nonuse) as part of their SBG to determine if there was any synergy between ZDV and ABC: stratum A if their existing SBG contained ZDV or stratum B if it did not. There were no limits on sample size within each stratum. Participants were then centrally randomized by ClinPhone at GlaxoSmithKline. The randomization schedule was loaded into the IVRS at ClinPhone and subsequently administered by them. The randomization schedule remained blinded until the database authorization was complete and after the data had been collected and cleaned. The patient, the investigator, the assessor, and GlaxoSmithKline were all blind to the treatment status of each patient. The ABC and placebo tablets were identical in appearance and taste. No steps, however, were undertaken to check the success of the blinding.

### Statistical Methods

All efficacy analyses were performed on the Intent-to-Treat (ITT) population, defined as all participants with data who were enrolled in the 12-wk phase of the study as randomized, regardless of actual treatment and the eventual outcome of study participation. Missing data were dealt with by carrying the last observation forward.

The NPZ was calculated as the actual test result minus the reference group mean divided by reference group SD. A summary NPZ was computed as the mean of the eight individual NPZ. Treatment groups were compared using the stratified Wilcoxon rank sum test on the change from baseline to week 12 in the summary NPZ. Study termination at week 12 was chosen on the basis of the previously mentioned randomized placebo controlled trial of ZDV in ADC [4].

Several retrospective exploratory sub-analyses were targeted at treatment effect within each of the three neuropsychological domains, effect of impairment at baseline on neuropsychological outcome and impact of background therapy. In addition to NPZ analyses, neuropsychological performance data were also examined using NP deficit score analysis as described by Heaton et al. [26]. The results from NP deficit score analysis did not differ from NPZ summary score method and only the results of the NPZ summary score will be presented.

## RESULTS

### Participant Flow

**Figure 1:**
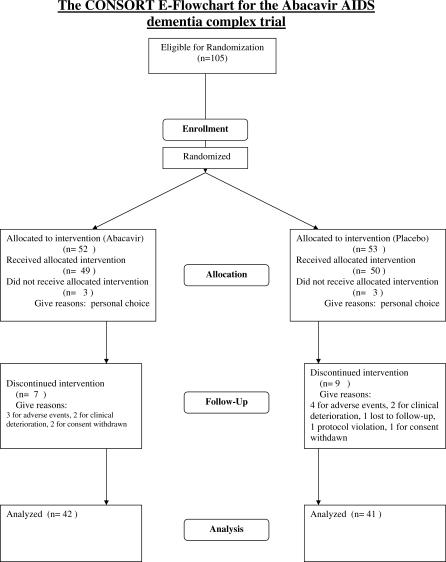
The CONSORT Flowchart for the ABC ADC Trial

A total of 105 participants were randomized: 52 to the ABC + SBG group and 53 to the SBG alone group. Three participants from each group withdrew from the study prior to baseline evaluation for personal reasons. There were three protocol violations: two participants with ADC Stage 3 and one with ADC Stage 0.5 were entered into the trial and allowed to complete the study. Of the 99 participants from whom data were collected, 42 (86%) in the ABC + SBG group and 41 (82%) in the SBG group completed the 12-wk randomized phase (Figure 1). Seven participants from the ABC + SBG group discontinued randomized therapy prematurely; three participants discontinued study drug due to adverse events (one of which proved fatal), two participants experienced clinical deterioration, and two participants withdrew consent. Nine participants from the SBG group discontinued randomized therapy prematurely; four participants discontinued study drug due to adverse events (one of which proved fatal), two participants experienced clinical deterioration, one participant was lost to follow-up, one participant was a protocol violation, and one participant withdrew consent. The trial commenced on September 4, 1996 and ended January 8, 1998.

### Baseline Data

Table 1 shows the baseline characteristics of the two groups, baseline measures and treatment group. The median plasma HIV-1 RNA was the only variable statistically significantly different between the groups. Only two participants were female despite there being no restrictions to entering females. All participants, except one, were receiving treatment with between one and five ARTs at study entry; approximately half were receiving triple ART. The distribution of nucleoside reverse transcriptase inhibitors (NRTIs), non-nucleoside reverse transcriptase inhibitors, and protease inhibitors (PIs) was fairly even between the groups. The most commonly administered PI was indinavir (37% of the ABC + SBG group and 40% of the SBG alone group).

**Table 1:**
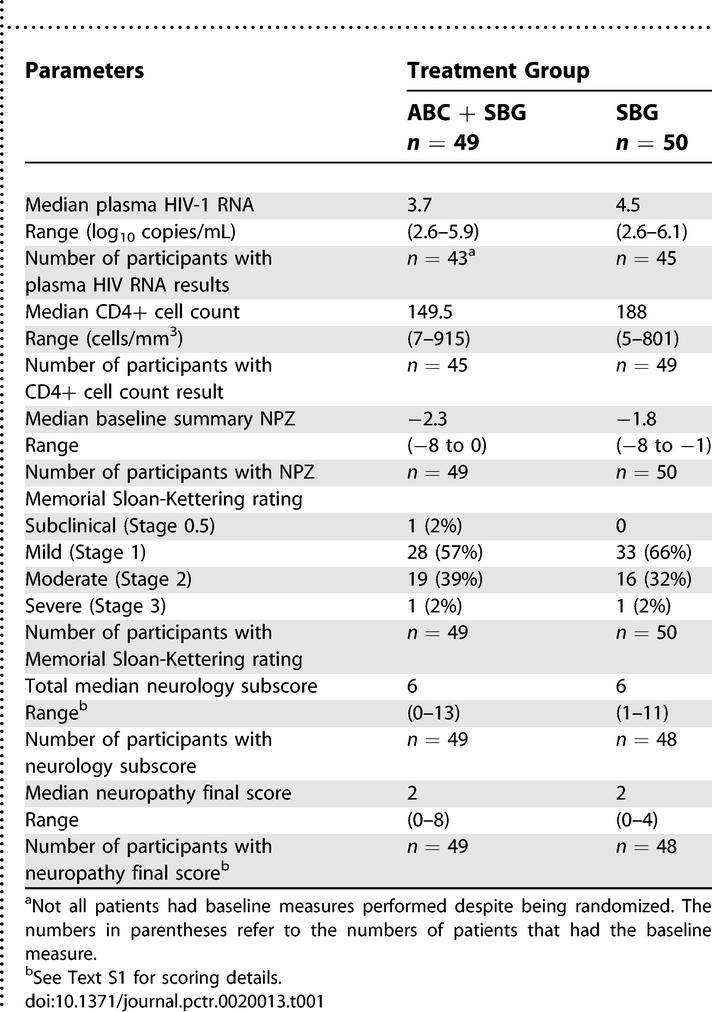
Baseline Measures

### Primary Outcomes

####  Neuropsychological performance.

Table 2 shows the improvement in both groups without any additional benefit from ABC by intention to treat analysis (*p* = 0.735).

**Table 2:**
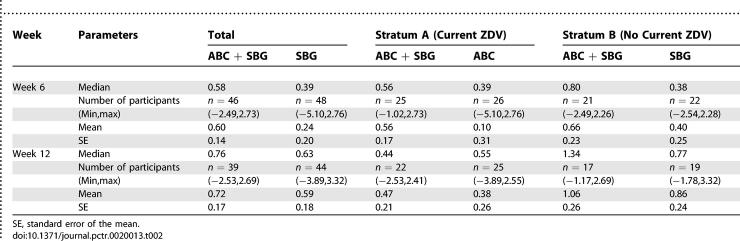
Summary Neuropsychological Z-Score Changes from Baseline: Intent-to-Treat

### Secondary Outcomes

####  CSF HIV-1 RNA.

The baseline HIV-1 RNA levels in CSF, measured in a subset of 79 participants, were similar between treatment groups; 25/45 (56%) of participants from the ABC + SBG group and 19/34 (56%) from the SBG alone group had HIV-1 RNA levels of ≤100 copies/mL. At week 12, the majority of available CSF samples had HIV-1 RNA levels below the limit of detection; 19/23 (83%) of participants from the ABC + SBG group and 12/16 (75%) of participants from the SBG alone group had CSF HIV-1 RNA ≤100 copies/mL. If the median reductions in CSF HIV-1 RNA from baseline are estimated using week 12 data (or using week 6 data if week 12 value is missing), the median HIV-1 RNA reduction for the ABC + SBG group of −0.64 log_10_ copies/mL (range −1.95 to +0.23, *n* = 13) was slightly more favorable than that observed for the SBG alone group of −0.26 log_10_ copies/mL (range −3.46 to +0.67, *n* = 8) but still not statistically significant. There was no statistically significant correlation between change in CSF HIV-1 RNA and changes in either NPZ or plasma HIV-1 RNA.

#### CSF markers of immune activation.

Changes in sTNF-R2, quinolinic acid, and β-2 microglobulin levels from baseline were compared with CSF HIV-1 RNA changes by treatment group. There were decreases in sTNF-R2 from baseline of 30 pg/mL (*n* = 7) for the ABC + SBG group and 88 pg/mL (*n* = 6) for the SBG alone group, and a decrease from baseline in quinolinic acid of 80 μg/mL (*n* = 8) for the ABC + SBG group and 186 μg/mL (*n* = 6) for the SBG group, but no significant associations with CSF HIV-1 RNA changes were identified. At baseline, 12/16 (75%) of participants from the SBG alone group and 22/25 (88%) of participants from the ABC + SBG group had CSF β-2 microglobulin levels <3.0 nmoles/mL; by week 12 decreases of 0.10 nmoles/mL (*n* = 7) in the ABC + SBG group and 0.12 nmoles/mL (*n* = 5) in the SBG group were observed. None of these changes in CSF markers reached statistical significance, even when the analyses were repeated as a percent change from baseline.

### Ancillary Analyses

####  Neurological evaluations.

There was improvement in the neurological subscore in both groups at week 6 to 5 and at week 12 to 4. There was no difference in the ADC stages at week 12 between the groups. Only two participants, both in the SBG group, demonstrated progression of ADC stage. There was no difference in neuropathy between the groups.

#### Plasma HIV-1 RNA.

**Figure 2:**
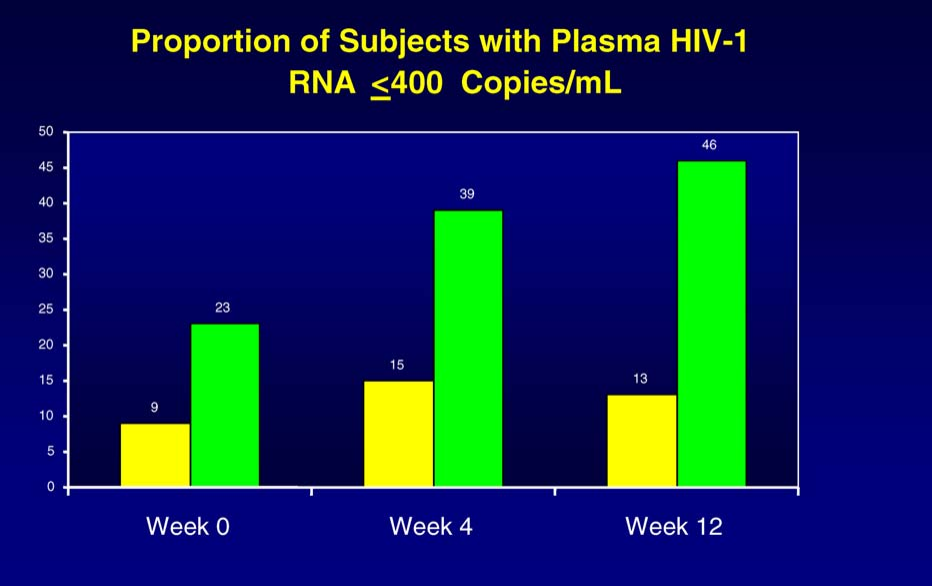
Antiviral Efficacy of ABC The percentage of participants in the two arms of the trial (ABC arm is the further bar to the right at each time point) on the y-axis with undetectable plasma HIV viral load at the beginning, week 4, and at the end of the trial (week 12).

Median change in plasma HIV-1 RNA from baseline showed no sustained response in either treatment group. In contrast, the percentage of participants with plasma HIV-1 RNA ≤400 copies/mL was statistically significant at week 12 (*p* = 0.002) in favor of ABC (Figure 2). By week 12, approximately 4-fold more participants in the ABC + SBG group had plasma HIV-1 RNA ≤400 copies/mL when compared to the SBG group: 5/39 (46%, ABC + SBG group) versus 17/37 (13%, SBG group). However, the groups were not balanced with respect to median plasma HIV-1 RNA at baseline, hence an additional analysis was conducted that examined changes in baseline detectability status at weeks 4 and 12. Participants were classified as improved (detectable at baseline but became undetectable at a later time point), worsened (undetectable at baseline and became detectable at a later time point), or no change (remained detectable or undetectable). Using this conservative analysis, which penalizes the ABC + SBG group for having a greater percentage of patients with undetectable plasma HIV-1 RNA at baseline, the results still favored the ABC + SBG group at week 12 (*p* = 0.029) (Figure 2).

#### Viral mutations in the RT gene in plasma and CSF.

Due primarily to low HIV-1 RNA copy number, only 67 baseline plasma samples and 26 baseline CSF samples were successfully genotyped. Approximately 90% (60/67) of participants for whom baseline genotyping of plasma viral isolates was successfully completed had evidence of mutations in the HIV-1 RT related to prior NRTI use, reflecting the extensive prior treatment of these participants. A baseline M184V plasma HIV-1 RT mutation was detected in 50/67 (75%) of participants. Three or more mutations associated with NRTI resistance at baseline were seen in 44/67 (66%) participants. Despite this, 46% of participants in the ABC + SBG group and 13% in the SBG alone group had plasma HIV-1 RNA ≤400 copies/mL at week 12.

CSF and plasma HIV-1 RT genotypes were discordant in 14/21 (67%) of baseline pairs. The majority of the differences were minor changes in ZDV/d4T mutation profiles. At baseline, one participant in the ABC + SBG group and six participants from the SBG alone group had the M184V mutation in virus from plasma but not from CSF.

Correlations between CSF HIV-1 RNA response at week 12 and baseline CSF viral genotype were available for five participants in the ABC + SBG group and six participants in the SBG alone group. In the ABC + SBG group, 3/5 baseline CSF isolates carried ≥3 NRTI-associated mutations and 2/3 had CSF HIV-1 RNA ≤100 copies/mL at week 12, and in the SBG alone group 5/6 baseline CSF isolates carried ≥3 NRTI-associated mutations and 3/5 had CSF HIV-1 RNA ≤100 copies/mL at week 12.

#### CD4+ cell count.

There was no notable difference in response between treatment groups with a median change from baseline at week 12 of +9 cells/mm^3^ (range 194 to +411 cells/mm^3^) in the ABC + SBG group and −1 cells/mm^3^ (range −162 to +278 cells/mm^3^) in the SBG alone group.

#### Pharmacokinetic measures.

Thirty-five CSF pharmacokinetic samples were obtained from participants randomized to receive ABC. The mean CSF ABC concentration increased consistently between the first and last collection intervals (0.126 μg/mL from 0.5–1.0 h, 0.356 μg/mL from 1.0–2.0 h, 0.575 μg/ml from 2.0–3.0 h, and 0.741 μg/ml from 3.0–4.0 h). While peak CSF concentrations may not have been attained due to the limited collection period (4 h post-dose in a 12-h dosing interval), the observed peak values are 9-fold greater than a representative IC_50_ for ABC (0.08 μg/mL) [17].

#### Post hoc exploratory analyses.

In order to further investigate why a difference between treatment groups was not detected, additional analyses were conducted on completion of the study. These focused on two hypotheses: (1) the presence of a PI in the therapy influenced the result and (2) the degree of neuropsychological impairment influenced the trial result. Analyses of these hypotheses did not reach statistical significance though positive trends were evident in favor of the ABC + SBG group in participants not receiving PIs and in those who were more severely neurologically impaired (Table 3).

**Table 3:**
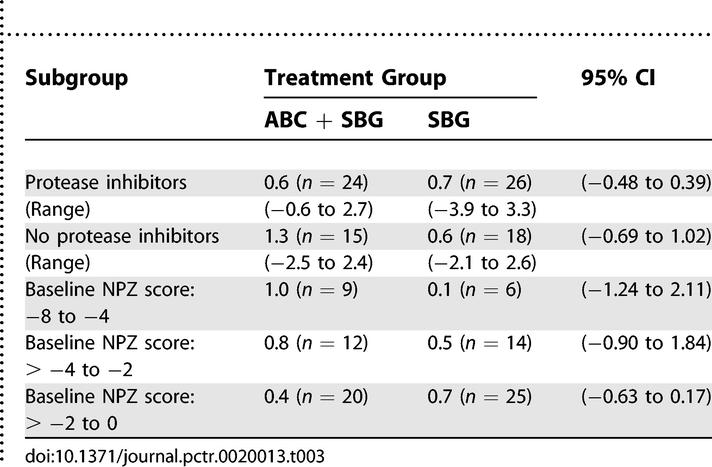
Summary of Median Neuropsychological Z-scores: Change from Baseline to Week 12 by Subgroup

### Adverse Events

In the ABC + SBG and SBG alone groups, 96% (ABC + SBG group) and 92% (SBG group) of participants experienced an adverse event during the randomized study period. The most frequent drug-related adverse events were nausea (41% ABC + SBG; 18% SBG alone), diarrhea (16% ABC + SBG; 8% SBG alone), malaise and fatigue (16% ABC + SBG; 12% SBG alone), nausea and vomiting (10% ABC + SBG; 4% SBG alone), and headache (10% ABC + SBG; 18% SBG alone). The incidences of these drug-related adverse events were not significantly different between treatment groups, with the exception of nausea in the ABC + SBG group (*p* = 0.01).

Three participants in the ABC + SBG group discontinued treatment before week 12 due to an adverse event, one of which was fatal: seizures followed by a cardiac arrest. The fatality was not considered related to ABC as the patient was a known epileptic, and there have been no reports of exacerbation of epilepsy control since ABC has been approved for use. One participant experienced an ABC-related hypersensitivity reaction, which resolved on cessation of ABC. Four participants in the SBG alone group experienced adverse events, one of which was fatal, respiratory arrest secondary to anemia. No notable trends were observed in any of the safety parameters examined, including the clinical laboratory values.

## DISCUSSION

### Interpretation

This 12-wk, randomized, placebo-controlled, double-blind study did not show any additional benefit from adding ABC to SBG ART in this group of participants with ADC; indeed, both treatment groups showed an improvement in neuropsychological performance during the 12 wk of study. Given that approximately two thirds of the patients in the trial had Stage 1 ADC and that Stage 1 overlaps with minor cognitive motor disorder as defined by the American Academy of Neurology, it seems likely that these results also pertain to less severe forms of HIV-related cognitive impairment. Analysis of the data reveals important issues in ADC trial design and patient management.

### Generalizability

There are two general theoretical reasons for the lack of additional demonstrable clinical activity: either ABC does not have efficacy or aspects of the trial design were flawed.

The median CSF HIV-1 RNA was reduced, albeit by a modest 0.64 log_10_ copies/mL over the 12 wk and ABC was present in the CSF in potentially efficacious concentrations, on average 9-fold greater than the IC_50_ reported for ABC for the wild-type virus. Moreover, there is published evidence for the systemic and neurological efficacy of ABC. This was obtained by searching PubMed for the keyword “abacavir.” Lanier et al. have shown in a meta-analysis of five studies where ABC was added to existing therapy that there was significant reduction in plasma HIV viral load even in patients with baseline NRTI mutations as long as the number of these did not exceed four [27]. The evidence for the neurological efficacy of ABC to date is more complex. Limoges et al. [28] have shown that in the brains of SCID mice with HIV encephalitis ABC decreased viral load by approximately one log while the efficacy of ZDV and d4T were variable but in general inferior. Indeed, ABC was very effective at limiting the spread of infection and microglial nodule formation. On the other hand, Kandanearatchi et al. [29] found that neither ABC nor ZDV made a significant difference to HIV replication in a human fetal brain aggregate model and that only d4T was efficacious when given 24 h prior to HIV infection. These findings do not accord with the known clinical efficacy of ZDV and raise potential concerns over the appropriateness of the model for assessment of antiretroviral efficacy. The only other study that tried parenthetically to assess the neurological efficacy of ABC is that by McCoig et al. [30]. In a small substudy, 13 children were randomized to receive ABC, ZDV, and 3TC compared to ten who received ZDV and 3TC. Approximately half in each group had neuropsychological impairment and this did not significantly change at week 48. However, there were several dropouts and the study was very significantly underpowered.

Given the above-mentioned points, it seems that there is adequate theoretical reason to expect ABC to have neurological efficacy. So perhaps this did not translate into practice because of flaws in trial design. In this regard, the first concern would be the presence of pre-existing resistance to the drug. Certainly, at baseline, 90% of participants had virus with evidence of resistance to the NRTIs and two thirds of isolates had three or more NRTI-associated mutations at baseline. The latter participants had less reduction in plasma HIV-1 RNA compared to those with virus with two or fewer mutations. As previously mentioned, recent data have shown that the greater the number of NRTI mutations, the greater the likelihood of ABC resistance [27]. Nonetheless, despite the presence of multiple mutations, in this limited group of patients significantly more participants treated with ABC had undetectable HIV-1 RNA at week 12 in the plasma and CSF. So, while this was clearly a factor it is unlikely to be the most important.

A second issue in trial design is that of the neurological stability of the patient groups at entry. An 8-wk period prior to entry was chosen because of pre-HAART data showing that the vast majority of improvement had occurred by 8 wk from commencement of a particular drug [4,31]. It was presumed that even though some patients were on HAART, approximately half in both groups at baseline, any improvement would still have stabilized by 8 wk from commencement of the regimen. At the time, this was based on the presumption that HAART would not make a significant impact on ADC because of the limited penetration into the brain of the most common component of such a regimen, namely the PIs. At the time of the trial, HAART consisted of two NRTIs and a PI. However, the fact that both the SBG and ABC groups improved at week 12 of the study (that is, after at least 5 mo on HAART) and that there were only two ADC progressions (both in the SBG group) strongly suggest that HAART does have efficacy in ADC leading to a lengthening of the time window. While there are no precise data on progression rates prior to the introduction of HAART for the different ADC stages, the mean time to death for Stage 2 ADC was 4.6 mo and for all ADC patients the time was 6 mo [31,32]. These figures therefore suggest that there should have been more ADC progressions in the study participants if HAART had no effect on dementia. The other interpretation, namely that the neuropsychological improvement was related to a learning effect for the tests seems unlikely: no similar improvement has been observed in other clinical studies and further analyses of the neuropsychological data comparing the results of testing in the memory domain (the most sensitive to learning effect) with the other tests of psychomotor function and reaction time did not show any significant differences. Thus, unlike the issue of baseline resistance, this does seem to be a major factor. In future ADC trials, patients should only be entered after a period of 5 mo on existing HAART to ensure stability.

A third issue in study design relates to whether or not the trial was appropriately powered. The sample size calculation was based on an estimated SD of 0.75, a difference between treatment group responses of 0.60, and a dropout rate of 20%–25% giving the study a 90% power. These estimates had been derived from previous MACS studies. However, the observed SDs on the change in neuropsychological perfor-mance from baseline were 1.27 (ABC + SBG group) and 1.15 (SBG group). Because the variability of the neuropsychological data was much greater than expected, the likelihood of detecting any treatment differences was compromised. Indeed the power of the study was reduced to 52%. With the same sample size, but with the larger SD of 1.20, only differences between treatment group responses on the order of 0.96 would have been detected. Had the study been planned with an SD of 1.20 (using all other assumptions) rather than 0.75, approximately 115 patients per treatment group (that is, approximately twice the number enrolled) would have been required. It should be noted that two HAART era studies conducted after the completion of this study also showed unexpectedly large SDs of 1 and 1.5 [33,34]. It therefore seems that the MACS cohort data that were used in the power analyses do not reflect ADC patients in the HAART era. Indeed, future trial designs should utilize these sample size re-estimations.

Lastly, and perhaps most importantly, the study was designed to include participants with a diagnosis of ADC not confounded by other illnesses, but there was no provision to restrict entry to those who had “active” disease (that is, ongoing brain damage). Some patients may have had virologically or immunologically inactive disease while others may have been in a “reparative” phase, perhaps analogous to head injury or stroke patients who may take months to slowly improve after the initial insult. Studies have shown a correlation between ADC severity and CSF HIV-1 RNA and β-2 microglobulin levels when used in pre-HAART era ADC patients without other confounding conditions [31]. However, there are no consensus criteria by which “active” versus “burnt out” disease versus a “reparative” phase can be differentiated. The fact that 56% of participants had a CSF HIV-1 RNA level below 100 copies/mL at baseline strongly suggests that many participants had a component of their neurological deficit that was unlikely to be driven by ongoing viral replication, and hence unlikely to be reversed by antiretroviral agents. Moreover, the fact that 83% of participants had CSF β-2 microglobulin concentrations that were normal for an HIV-infected population also suggests that most patients had a significant component of their deficit that was not driven by immune activation or active viral replication. How can this lack of virological and immunological activity be reconciled with the fact that most of the patients improved? We suggest that this may be related to the patients being in a reparative phase of the illness: possibly new synaptic connections are being made. It could be hypothesized that CSF HIV-1 RNA >100 copies/mL and β-2 microglobulin >3 nmoles/L should identify “active” ADC. However, in a recent manuscript, we thoroughly explored this with a negative result [35]. Elevation or non-elevation of any one or combination of CSF markers at entry did not predict stability, improvement, or worsening of ADC at week 12, although there was a weak negative association with the degree of elevation of CSF viral load at entry. Future studies will need to focus on markers that can identify active ADC, inactive or “burnt out” ADC, and address the possibility of some patients entering a reparative phase. It is still possible that CSF HIV viral load may be a good marker of activity if the limit of detection can be reliably measured down to 10 cpml, for example. Thus, the issue of CSF markers will need to be approached in two ways: the refinement of existing markers with greater sensitivity and the development of new markers. Neuroimaging with spectroscopic analyses may also be able to address the issue of ADC activity.

Moreover, this study highlights an important change in CSF ADC markers in HAART-treated patients. No longer does the CSF compartment as assessed by HIV RNA and immune activation markers such as β-2 microglobulin reflect what is occurring in the brain: there is no relationship to the severity of ADC or to the risk of its worsening. This has been confirmed independently in another publication using a different cohort of patients [36]. There are two explanations: either the CSF compartment still reflects brain disease, but current CSF markers are insensitive, or the CSF compartment has become “separate” to the brain through HAART—perhaps through differential activity in these compartments. Future studies will need to address these possibilities.

### Overall Evidence

This study highlights four issues that are critical in the design of future ADC trials and in individual patient management. Firstly, if a new antiretroviral agent is to be trialed in ADC the details of resistance mutations to it should be known before commencement of the trial so that patients with resistant virus can be excluded from the trial. A potential means of circumventing this problem in the case of a novel agent where resistance patterns have not yet been described (this was the case for ABC at the beginning of this trial) would be to recruit only antiretroviral naïve patients. However, this would mean that a large number of study sites would have to be involved to enable the requisite number of patients to be reached. Secondly, HAART has a beneficial effect on ADC which continues for at least 5 mo after commencement of therapy. Thus, future ADC trials should enroll patients after at least 5 mo of HAART. Thirdly, the variability in neuropsychological performance over time is greater than previously reported. These data should be used to estimate the required sample size in future trials. Fourthly, ADC patients should only be recruited into future anti-retroviral trials if they have active virally mediated ADC. At an individual patient level, it is not possible to be certain of the degree of activity of ADC in a HAART-treated patient. Consequently, clinicians are forced to consider an empirical trial either of intensification or change of HAART. Thus, markers that allow “real time” evaluation of ADC activity, as opposed to its retrospective identification by history, are urgently needed. In this regard, emerging data on the CSF concentrations of the neuronal markers neurofilament and perhaps t-tau are promising [37–39].

While this trial delivered a negative result, it highlights important factors that must be incorporated into a new design methodology for future ADC trials in HAART-treated patients.

## Supporting Information

CONSORT Checklist Click here for additional data file. (50 KB PDF)

Trial Protocol Click here for additional data file. (4.5 MB PDF)

Text S1 Clinical Evaluation Form Click here for additional data file. (1.7 MB PDF)

## Abbreviations

ABC: abacavir
ACTG: AIDS Clinical Trials Group
ADC: AIDS dementia complex
ART: antiretroviral therapy
CSF: cerebrospinal fluid
HAART: highly active antiretroviral therapy
NPZ: neuropsychological Z score
NRTI: nucleoside reverse transcriptase inhibitor
PI: protease inhibitor
RT: reverse transcriptase
SBG: stable background antiretroviral therapy
SD: standard deviation
TNF: tumor necrosis factor
ZDV: zidovudine
