# Supplementary material for: Factors in AIDS Dementia Complex Trial Design: Results and Lessons from the Abacavir Trial
Source: PLoS Clin Trials. 2007 Mar 30;2(3):e13. doi: 10.1371/journal.pctr.0020013 (PMC1845158; doi:10.1371/journal.pctr.0020013)
Supplement: Trial Protocol [file pctr.0020013.sd002.pdf]

CONFIDENTIAL

GLAXO WELLCOME
Research and Development

Department/Division: European Antiviral, Clinical Research

Document Code: BQPL/96/0002/04

Total No. of Pages: 160
(count excludes Cover Sheet)

Confidentiality Level: C

Date of Document: 28 August 1997

Title: **A Phase III, Randomised, Double-Blind, Multicentre Study to Evaluate the Safety and Efficacy of 1592U89 in HIV-1 Infected Patients with AIDS Dementia Complex - Incorporating Amendment Number 4**

Protocol Number CNAB3001

Author(s): Carmen Romero

Purpose/Summary:

Revision Chronology:

BQPL/96/0002	1 July 1996	Original Document
BQPL/96/0002/01	25 September 1996	Protocol Amendment Number 1
BQPL/96/0002/02	17 February 1997	Protocol Amendment Number 2
BQPL/96/0002/03	13 June 1997	Protocol Amendment Number 3
BQPL/96/0002/04	28 August 1997	Protocol Amendment Number 4

Keywords:

Typist Ref.: TPC - Medical (UK) **Electronic Filename:** BQPL/96/0002/04

Distribution:

Records Centre, Internal Information Services (2 copies, plus one copy for RDM Central Records at RTP)

Original to Medical Business Records

©Copyright: Glaxo Wellcome, all rights reserved. This document and its contents are the property of and confidential to Glaxo Wellcome. Unauthorised copying or use of this document is prohibited. Removal of this document from Company premises without managerial approval is expressly forbidden.

CONFIDENTIAL

GLAXO WELLCOME
Research and Development

BQPL/96/0002/04

A Phase III, Randomised, Double-Blind, Multicentre Study to Evaluate the Safety and Efficacy of 1592U89 in HIV-1 Infected Patients with AIDS Dementia Complex - Incorporating Amendment Number 4

Protocol Number CNAB3001

Carmen Romero
European Antiviral, Clinical Research

28 August 1997

C Romero

28th August 1997
Date

©Copyright: Glaxo Wellcome, all rights reserved. This document and its contents are the property of and confidential to Glaxo Wellcome. Unauthorised copying or use of this document is prohibited. Removal of this document from Company premises without managerial approval is expressly forbidden.

BQPL/96/0002/04

GLAXO WELLCOME
Research and Development

BQPL/96/0002/04

A Phase III, Randomised, Double-Blind, Multicentre Study to Evaluate the Safety and Efficacy of 1592U89 in HIV-1 Infected Patients with AIDS Dementia Complex - Incorporating Amendment Number 4

STUDY CHAIR

Bruce James Brew, MBBS MD FRACP
Associate Professor of Medicine
University of New South Wales
Head of Department of Neurology
National Centre in HIV Epidemiology and Clinical Research
St. Vincent's Hospital, Medical Centre
376 Victoria Street
Darlinghurst
Sydney
Australia
Phone: (61) (2) 332-3421
Fax: (61) (2) 332-1837
E-Mail: Brew@UNSW.EDU.AU

©Copyright: Glaxo Wellcome, all rights reserved. This document and its contents are the property of and confidential to Glaxo Wellcome. Unauthorised copying or use of this document is prohibited. Removal of this document from Company premises without managerial approval is expressly forbidden.

CONTENTS

SUMMARY .... NO TAG

NO TAG. INTRODUCTION .....
NO TAG
NO TAG Background .....
NO TAG
NO TAG Rationale .....
NO TAG
NO TAG Study Drug: 1592U89 .....
NO TAG
NO TAG General Pre-clinical Information .....
NO TAG
NO TAG In Vitro Anti-HIV Activity .....
NO TAG
NO TAG Toxicology .....
NO TAG
NO TAG Clinical Studies .....
NO TAG

NO TAG. OBJECTIVES .....
NO TAG
NO TAG Primary Objectives .....
NO TAG
NO TAG Secondary Objectives .....
NO TAG

NO TAG. STUDY DESIGN .....
NO TAG
NO TAG Dose Rationale .....
NO TAG

NO TAG. TRIAL SUBJECT SELECTION .....
NO TAG
NO TAG Inclusion Criteria .....
NO TAG
NO TAG Exclusion criteria .....
NO TAG
NO TAG Number and Source of Subjects .....
NO TAG

NO TAG Subject Stratification and Randomisation .....
NO TAG
NO TAG Subject Enrolment .....
NO TAG

NO TAG. CLINICAL TRIAL MATERIAL .....
NO TAG
NO TAG Study Drugs .....
NO TAG
NO TAG Supply of CTM to the Study Site .....
NO TAG
NO TAG Dose Regimens .....
NO TAG
NO TAG Clinical Trial Material (CTM) Management .....
NO TAG
NO TAG Dispensing of CTM .....
NO TAG
NO TAG Disposition of CTM .....
NO TAG
NO TAG Concomitant Medication .....
NO TAG

NO TAG. MEASUREMENTS AND EVALUATIONS .....
NO TAG
NO TAG Pre-Entry Evaluations .....
NO TAG
NO TAG On-Study Evaluations (Randomised phase) .....
NO TAG
NO TAG Open Phase Evaluations .....
NO TAG
NO TAG Extra-Assessment Evaluations (Unscheduled Visits) .....
NO TAG
NO TAG Last Visit Evaluation (Premature Discontinuation) .....
NO TAG
NO TAG Follow-up Evaluation (Off-drug) .....
NO TAG
NO TAG Pharmacokinetic Evaluations .....
NO TAG
NO TAG Collection of Pharmacokinetic Samples .....
NO TAG
NO TAG Collection of Plasma with Corresponding CSF Samples .....
NO TAG

NO TAG Analytical Methodology .....
NO TAG
NO TAG Virology Evaluations .....
NO TAG
NO TAG Collection of Virology Samples .....
NO TAG
NO TAG Adverse Event Reporting .....
NO TAG
NO TAG Recording .....
NO TAG
NO TAG Documentation .....
NO TAG
NO TAG Definition of an Adverse Event (AE) .....
NO TAG
NO TAG Definition of a Serious Adverse Event (SAE) .....
NO TAG
NO TAG Dose Modification due to Toxicity or Adverse Experience .....
NO TAG
NO TAG Neuropsychological Evaluations .....
NO TAG
NO TAG Neurological Evaluations .....
NO TAG
NO TAG ADC rating (MSK scale) .....
NO TAG
NO TAG Validation of Dementia End-Points .....
NO TAG
NO TAG Validation of HIV Clinical End-Points .....
NO TAG
NO TAG Patient and Care Given Impact Evaluations .....
NO TAG
NO TAG Patient Health Status Questionnaires (MOS-HIV) .....
NO TAG
NO TAG HIV Caregiver Impact Assessment (HIV-CIA) .....
NO TAG
NO TAG Questionnaire Translations Available .....
NO TAG
NO TAG Administration of Questionnaires .....
NO TAG

NO TAG. DATA MANAGEMENT AND ANALYSIS .....
NO TAG
NO TAG Case Report Form .....
NO TAG
NO TAG Sample Size .....
NO TAG
NO TAG Statistical Analysis .....
NO TAG
NO TAG Pharmacoeconomic Analysis .....
NO TAG

NO TAG. STUDY MANAGEMENT .....
NO TAG
NO TAG Approval and Consent .....
NO TAG
NO TAG Approval from Regulatory Authorities .....
NO TAG
NO TAG Ethical Approval .....
NO TAG
NO TAG Informed Consent .....
NO TAG
NO TAG Patient Withdrawal or Discontinuation .....
NO TAG
NO TAG Study or Site Closure .....
NO TAG
NO TAG Study Monitoring .....
NO TAG
NO TAG Source Document Verification .....
NO TAG
NO TAG Audit .....
NO TAG
NO TAG Records Retention .....
NO TAG
NO TAG Documentation and Publication .....
NO TAG
NO TAG Compensation for Injury .....
NO TAG

9. EXTENSION PHASE 45
9.1 Objectives 45
9.2 Rationale 45

9.3 Study Design 45
9.4 Subject Selection 45
9.4.1 Inclusion Criteria 45
9.4.2 Exclusion Criteria 45
9.5 Concomitant Medications 46
9.6 Clinical Trial Material 46
9.6.1 Dose & Regimens 46
9.7 Measurements & Evaluations 46
9.7.1 On Study 46
9.7.2 Study Discontinuation 46
9.7.3 Follow-up (Off-drug) 47

REFERENCES .... NO TAG

NO TAG. VIRAL LOAD DETERMINATIONS IN PLASMA (HIV RNA PCR) ..... NO TAG
NO TAG Required Materials ..... NO TAG
NO TAG Collection and Processing of Blood Samples ..... NO TAG
NO TAG Collection and Processing of Cerebro Spinal Fluid Samples ..... NO TAG
NO TAG Virological Analysis ..... NO TAG
NO TAG CSF Markers Analysis ..... NO TAG

NO TAG. VIRAL PHENOTYPE (SI/NSI) ..... NO TAG

NO TAG. SHIPMENT OF SAMPLES ..... NO TAG

NO TAG.COLLECTION OF BIOLOGICAL SAMPLES FOR PHARMACOKINETIC EVALUATIONS .... NO TAG
NO TAG Blood Samples ..... NO TAG
NO TAG Cerebrospinal Fluid Samples ..... NO TAG
NO TAG Instructions for the Use of Bar Code Labels ..... NO TAG

NO TAG. SHIPMENT OF SAMPLES ..... NO TAG

Protocol Amendment Number 1 .... NO TAG

Protocol Amendment Number 2 .... NO TAG

Protocol Amendment Number 3 124

Protocol Amendment Number 4 129

LIST OF TABLES

NO TAG. Mean (%CV) Pharmacokinetic Parameters for 1592U89 Following Single, Oral Doses of 100, 300, 600, 900 and 1200 mg Under Fasting Conditions (n=8) .... NO TAG

NO TAG. Immunological and Virological Responses - Preliminary Results of 1st Cohort - 1592U89 200 mg TID .... NO TAG

NO TAG. CSF Sample Time–Frame Assignments .... NO TAG

NO TAG. CSF/Plasma Samples Coordinated Time Frames .... NO TAG

NO TAG. Plasma Samples Time Frame Assignments .... NO TAG

LIST OF FIGURES

NO TAG. Mean (± SE) Plasma 1592U89 Concentration vs. Time Curves Following Single, Oral Doses of 100, 300, 600, 900 and 1200 mg Under Either Fasted or Fed Conditions . . . . NO TAG

LIST OF APPENDICES

NO TAG: STUDY FLOW CHART ..... NO TAG

Appendix A1: Extension Phase Study Flow-Chart 54

NO TAG: AIDS DEMENTIA COMPLEX RATING SCALES ..... NO TAG

NO TAG: Memorial Sloan Kettering Rating Scale ..... NO TAG

NO TAG: American Academy of Neurology Diagnosis of HIV-1 Associated Dementia Complex and Cognitive Impairment ..... NO TAG

NO TAG: KARNOFSKY PERFORMANCE SCALE ..... NO TAG

NO TAG: NEUROPSYCHOLOGICAL EXAMS ..... NO TAG

NO TAG: NEUROLOGICAL EXAMS ..... NO TAG

NO TAG: LISTING OF SPECIFIC CLINICAL LABORATORY TESTS . NO TAG

NO TAG: Listing of Specific Clinical Laboratory Tests: Blood ..... NO TAG

NO TAG: Listing of Specific Clinical Laboratory Tests: Cerebrospinal Fluid ..... NO TAG

NO TAG: VIROLOGY SAMPLES PROCEDURES ..... NO TAG

NO TAG: PHARMACOKINETIC SAMPLES PROCEDURES ..... NO TAG

NO TAG: ADVERSE EXPERIENCE DATA HANDLING GUIDELINES ..... NO TAG

NO TAG: TABLES FOR GRADING THE INTENSITY OF ADVERSE EXPERIENCES ..... NO TAG

NO TAG: Laboratory Test Abnormalities ..... NO TAG

NO TAG: Clinical Adverse Experiences ..... NO TAG

NO TAG: OPERATING PRINCIPLES OF END-POINT COMMITTEE ..... NO TAG

NO TAG: PATIENT AND CAREGIVER QUESTIONNAIRES ..... NO TAG

NO TAG: Patient Health Status Questionnaires ..... NO TAG

NO TAG: HIV Caregiver Impact Assessment (HIV-CIA) ..... NO TAG

NO TAG: Other Pharmacoeconomic Evaluations ..... NO TAG

NO TAG: ABPI COMPENSATION ..... NO TAG

NO TAG: PROJECT-SPECIFIC EXEMPTIONS TO SERIOUS ADVERSE EVENTS ..... NO TAG

LIST OF ABBREVIATIONS

AAN	American Academy of Neurology
ABPI	Association of the British Pharmaceutical Industry
ADC	AIDS Dementia Complex
AIDS	Acquired Immunodeficiency Syndrome
ALT	Alanine Aminotransferase (SGOT)
AST	Aspartate Aminotransferase (SGPT)
AUC	Area Under the Curve
BBB	Blood Brain Barrier
β-HCG	β Human Chorionic Gonadotrophin
CBC	Complete Blood Count
CDC	Centers for Disease Control
CL/F	Apparent Total Clearance
C_{max}	Maximum Concentration
CNS	Central Nervous System
CPK	Creatinine Phosphokinase
CRF	Case Report Form
CSF	Cerebrospinal Fluid
CTM	Clinical Trial Material
CV	Coefficient of Variation
ddC	Zalcitabine, HIVID
ddI	Didanosine, VIDEX
d4T	Stavudine, ZERIT
EC	Ethics Committee
EDTA	Ethylene Diaminetetracetic Acid
GI	Gastrointestinal
HBsAg	Hepatitis B Virus Surface Antigen
HCV Ab	Hepatitis C Virus Antibody
HIV	Human Immunodeficiency Virus type 1
HPLC	High Performance Liquid Chromatography
IC_{50}	Fifty Percent Inhibitory Concentration
IQ	Intelligence Quotient
IRB	Institutional Review Board
MCV	Mean Cell Volume
MOS	Medical Outcome Study (questionnaire)
MSK	Memorial Sloan Kettering
NNRTI	Non-nucleoside Reverse Transcriptase Inhibitor
NSI	Non Syncytium Inducing
OI	Opportunistic Infection
PBMC	Peripheral Blood Mononuclear Cells
PCR	Polymerase Chain Reaction
RNA	Ribonucleic Acid
RT	Reverse Transcriptase
SAE	Serious Adverse Event
SD	Standard Deviation

SE	Standard Error
SI	Syncytium Inducing
T_{max}	Time taken for drug to reach maximum plasma concentration
$T_{1/2}$	Half life or time taken for drug to reach 50% of the C_{max}
VDRL	Venereal Disease Research Laboratory
WAIS-R	Wechsler Adult Intelligence Scale - Revised
WHO	World Health Organisation
ZDV	Zidovudine, RETROVIR, AZT
3TC	Lamivudine, EPIVIR

A Phase III, Randomised, Double-Blind, Multicentre Study to Evaluate the Safety and Efficacy of 1592U89 in HIV-1 Infected Patients with AIDS Dementia Complex - Incorporating Amendment Number 4

SUMMARY

This is a phase III randomised, double-blind, multicentre study to evaluate the safety and efficacy of 1592U89 in patients with AIDS dementia complex. Patients may continue to receive other antiretroviral agents which are approved for marketing in at least one country. Agents available by expanded access or other parallel track programs will be assessed on a case-by-case basis with the sponsor.

The primary objectives of the study will be to evaluate the benefit on neuropsychological performance in ADC patients as well as the safety and tolerance. Secondary objectives will include changes in clinical dementia, neurological status, survival, AIDS defining conditions, virological and immunological markers both in plasma and CSF.

A minimum of 90 patients will be enrolled across sites in Europe, Australia, Canada and the US. Patients will be pre-stratified into groups A or B depending on whether their existing therapy contains zidovudine or not. Each stratum will then be centrally randomised separately to receive 600 mg of 1592U89 or matching placebo every twelve hours. Study medication will be administered in addition to the patient's existing antiretroviral therapy which may include a combination of agents.

Change or addition of background antiretroviral agents will not be permitted during the 8 week pre-entry period or the randomised phase of the study.

The study will be comprised of a 12 week randomised phase followed by an optional open phase of 40 weeks when open label 1592U89 will be offered. Study participants who progress on treatment or experience severe antiretroviral drug toxicity not related to 1592U89 may be offered to commence the open-label phase of the study after a minimum of 6 weeks on study have been completed. Patients will be defined as having progressed on study treatment if their ADC clinical status deteriorates by one stage on the MSK rating scale (ie. from stage 1 to 2 or stage 2 to 3). There will be an end-point committee to validate all the clinical dementia progressions occurring in the study.

Patients will be evaluated every 2 weeks during the randomised phase of the study, every 2-4 weeks for the first 8 weeks of the open phase, and every 8 weeks for the remainder of the trial.

1. INTRODUCTION

1.1 Background

The spectrum of HIV-1 involvement in the CNS is wide. Neurological manifestations of HIV-1 can be divided into primary and secondary depending on whether the damage is thought to be mediated by HIV-1 directly or through opportunistic processes as a result of the severe immunosuppression induced by HIV-1. The most predominant manifestation of direct HIV involvement in the brain is a dementia syndrome which has been designated by a variety of terms including HIV-1 encephalopathy, subacute encephalitis, AIDS-related dementia, AIDS dementia complex (ADC) and more recently HIV-associated dementia.

There is evidence to suggest that the nervous system is exposed to HIV-1 during the initial stages of infection. Schmid et al (1) examined provirus levels in CD4 cells from blood and CSF in relation to the stage of infection and found that there was a significant CSF penetration in asymptomatic individuals. They also showed that the proviral load was higher in CSF than in blood regardless of disease stage. In addition, HIV can reach the brain rapidly following primary infection. Davis et al (2) reported a case where a 68 year old man was accidentally inoculated with HIV contaminated white blood cells and subsequently died within 15 days of the transfusion. At the autopsy, HIV-1 was isolated from the brain, furthermore proviral DNA was detected in multiple areas of the brain.

Despite this early presence of HIV-1 in the CNS, ADC does not generally develop until the individual becomes severely immunosuppressed. One possible explanation for this apparent "delay" may be that, as the systemic HIV disease and CD4 cell loss progresses, the CNS infection can no longer be controlled and compensatory immune mechanisms are triggered. Such activation of macrophages and microglia may result in the production of neurotoxic factors that cause bystander damage of neurological tissue. In addition, it is likely that the previously controlled infection, possibly localised in the subcortical areas of the brain and the meninges, now expands and becomes amplified by the circulating infected cells from the systemic circulation.

Neuronal damage and destruction has been observed in the absence of infection. It is thought that the protagonist cells are the infected macrophages which initially originate from the systemic circulation and eventually reach the brain to spread infection to microglial cells and possibly astrocytes. Emerging *in vitro* research is providing a good insight into the substances and mechanisms involved in neuronal damage. Viral proteins such as gp120 together with activation factors and toxins secreted by macrophages (including glutamate-like neurotoxic molecules, free radicals, eicosanoids, quinolinic acid, neopterin and cytokines such as TNF-α) have all been shown

to cause neuronal damage through a series of mechanisms that ultimately result in an excessive influx of calcium ions into the neurons which leads to neuronal death (3-9).

The pathological findings at autopsy in patients with ADC are heterogeneous and may not always correlate with severity of clinical symptoms. Some patients with severe dementia show surprisingly mild neuropathological changes and similarly, severe neuropathology has been observed in the absence of clinical neurological symptoms. Overall however, the neuropathological abnormalities observed in ADC can be divided into four overlapping categories:

1. White matter pallor and gliosis. This is the most common finding at autopsy in patients with ADC, albeit the least pathologically specific and is typically accompanied by reactive astrocytosis and increased numbers of microglia. It is sometimes the sole neuropathological finding in patients dying with milder ADC.

2. Multinucleated-cell (or giant cell) encephalitis characterized by the presence of giant cells which represent the result of fused macrophages infected by HIV. It is a hallmark of productive HIV and it is usually observed in individuals with severe ADC.

3. Vacuolar myelopathy involving periaxonal vacuolation in thoracic segments of the spinal cord. It is a characteristic finding in patients with myelopathy, presenting with spastic paraparesis and sensory ataxia (10-11).

4. Cortical atrophy with neuronal loss is a frequent finding that may be associated with productive HIV-1 infection of the brain (12-13).

Clinically, the major characteristic of ADC is initially that of a subcortical dementia with dominant slowing of intellectual processing and poor attention. Patients complain of decreased concentration and forgetfulness. Complex tasks at work or in the home become increasingly difficult and take longer to complete. With advancing disease, they perform increasingly poorly on tasks requiring concentration and attention such as word and digit reversals and serial subtractions. In conjunction with these symptoms there are motor complaints manifested as clumsiness, sloppy handwriting, tremor and poor balance. Frequent early findings on examination include nonfocal motor abnormalities, such as retardation of rapid successive and alternating movements of the extremities and eyes. Abnormal reflexes are common, with generalised hyperreflexia along with release signs such as snout or glabellar responses. With progression there may be symptomatic difficulty with balance or coordination. In more advanced disease ataxia and, subsequently, leg weakness limit ambulation. Patients with early or predominating spastic ataxic gait usually will be shown to have vacuolar myelopathy pathologically (14). In those with a severe progressive course, the end stage of ADC is nearly vegetative, patients lie in bed with a vacant stare, paraparesis and incontinence.

The speed of progression varies considerably between patients. Complaints develop subacutely over a number of weeks to months in patients with advanced immunosuppression. However, some patients may progress without experiencing major systemic complications of HIV-1 (15).

Diagnosis of ADC is by recognition of the characteristic cognitive, behavioural and motor dysfunctions as well as the exclusion of secondary CNS opportunistic infections and/or neoplasms due to the fact that many of these conditions present common symptomatology. Other factors that may mask, mimic or exacerbate the features of ADC include severe systemic infection, cachexia, metabolic toxicity due to organ failure and medications that affect CNS function such as narcotic analgesics, sedatives, benzodiazepines, antidepressants, anti-psychotic agents and other psychoactive drugs commonly administered to late stage patients. These confounding factors must be taken into consideration when diagnosing or assessing ADC.

Two classifications currently exist to define dementia associated with HIV, one issued by the American Academy of Neurology (16) and the other by the World Health Organisation (17). For the assessment of entry criteria the AAN classification will be used (Appendix B2). To evaluate the level of progression of ADC throughout the study, the rating scale designed by Price and Brew (18) will be used; this scale is also referred to as the Memorial Sloan Kettering or MSK scale.

1.2 Rationale

Data from two major cohorts in Europe and the US have yielded important ADC prevalence figures. ADC has been reported to be present at the time of AIDS diagnosis in 4.5% and 3.3% of cases in the European and US cohorts respectively. The overall prevalence in AIDS patients was found to be 12% and 15% respectively. The US cohort reported an annual incidence of approximately 7% during the 2 years after AIDS, with a median survival after diagnosis of 6 months (19,20). The above data represents dementia cases at MSK stages 2 and above, if stage 1 diagnoses are included, the prevalence increases to 30% (Perdices and Brew, Journal of Neurology, Neurosurgery and Psychiatry in press).

To date zidovudine has been the main antiretroviral used in ADC. Epidemiologically, the introduction of zidovudine in the late 80s was accompanied by a marked reduction in the incidence and prevalence of ADC (19,21). Therapeutically, zidovudine has been shown to enhance performance on quantitative neuropsychological testing in both affected adults and children (22-25) as well as reduce CSF surrogate markers including β_2 microglobulin, neopterin and quinolinic acid (4, 26-28). These observations on the neuroprotective effect of an antiretroviral drug suggests that the virus must somehow drive the pathogenetic mechanisms described and at the same time offer some hope for antiretroviral therapy to treat and possibly prevent ADC.

Despite zidovudine's invaluable contribution to the treatment of ADC, its effect appears to be short-lived. In addition, patients that develop ADC are often intolerant or have strains of virus which are resistant to zidovudine. No other alternative antiretroviral has been identified to date and little clinical neurological data exists from the newer compounds such as protease inhibitors, non-nucleoside reverse transcriptase inhibitors, or other nucleoside reverse transcriptase inhibitors. Therefore the need still exists to search for additional drugs that are able to exert their effect on the CNS.

1592U89 has been shown to cross the blood brain barrier in animals and humans to a similar extent to zidovudine (see sections 1.3.1 and 1.3.4.2).

In addition, *in vitro* data suggest that 1592U89 has better inhibitory activity in macrophages (0.65 μM) than in peripheral blood lymphocytes (4.0 μM) (M. St. Clair unpublished data), this observation could potentially make 1592U89 a good candidate to treat macrophage infection in the CNS which appears to be the main vehicle driving the neuropathological changes seen in ADC.

Preliminary clinical results, from patients receiving 12 weeks of treatment, have shown 1592U89 to be more potent than other nucleoside analogues studied to date and similar to that seen with the newer protease inhibitors. In addition, the preliminary viral load data from this 12 week trial have shown good sustainability of the antiviral response. The described profile of 1592U89, whilst it is still at an early stage of development, indicates that it is a promising candidate both against systemic and neurological HIV disease.

1.3 Study Drug: 1592U89

1.3.1 *General Pre-clinical Information*

1592U89 succinate, a carbocyclic 2′,3′-ene nucleoside, has the following structure:

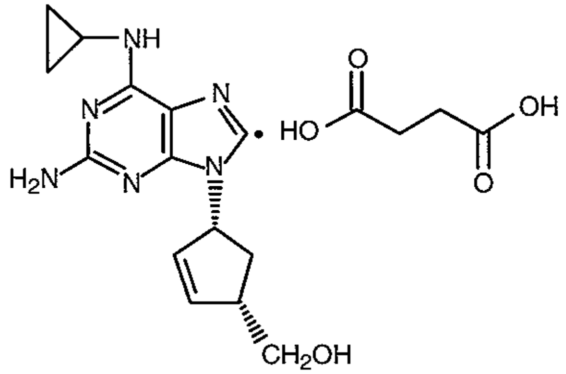

structure of 1592U89
succinate

1592U89 is activated intracellularly to the triphosphate derivative of the carbocyclic guanine analog (1144U88, (1R,4S)-9-(4-(hydroxymethyl)-2-cyclopenten-1-yl) guanine). The triphosphate of 1144U88 is a potent inhibitor of HIV reverse transcriptase *in vitro* with a Ki of 0.02 μM.

Pharmacokinetic evaluation showed good oral bioavailability (92% in mice, 77% in monkeys) in the anticipated therapeutic range.

Penetration of 1592U89 into monkey CSF and rat brain was comparable to that of zidovudine. In rats the average brain/plasma concentration ratios for 1592U89 and ZDV were 0.13 and 0.11 respectively. In monkeys, CSF/plasma concentration ratios for 1592U89 and ZDV were 0.26 and 0.21 respectively. No demonstrable CNS adverse effects were observed.

1.3.2 *In Vitro Anti-HIV Activity*

The anti-HIV activity 1592U89 was evaluated *in vitro*, using a laboratory strain of HIV (IIIb strain) cultured in a range of cell types, including MT-4 cells (a human leukemic cell line transformed with HTLV-1), peripheral blood lymphocytes (PBLs) and macrophages. The mean fifty percent inhibitory concentrations (IC_{50}) in these cells were 4.0, 3.7 and 0.65 μM, respectively. In comparison to the *in vitro* results with HIV_{IIIb}, the mean (± SD) IC_{50} for 1592U89 against eight fresh clinical isolates of HIV isolated from zidovudine-naive patients and cultured in PBLs was 0.26 (± 0.18) μM, compared to 0.12 (±0.07) μM for ZDV using the same assay system.

High level resistance to 1592U89 is not rapidly selected *in vitro*; multiple mutations in HIV-1 RT at codons K65R, L74V, Y115F and/or M184V (29) appear to be required to confer approximately 11 fold reduction in susceptibility to 1592U89 (M. Tisdale unpublished data). In addition, clinical isolates of HIV with decreased sensitivity to ZDV are not cross-resistant to 1592U89. In vitro studies (using $HIV\text{-}1_{IIIb}$ in MT-4 cells) have shown 1592U89 to have strong synergistic anti-HIV activity when combined with ZDV, 3TC, saquinavir, and the protease inhibitor 141W94, weaker synergy has been observed with ddI and ddC (M.St. Clair unpublished data).

1.3.3 *Toxicology*

In toxicology studies completed to date, there have been limited effects associated with 1592U89 succinate.

In vitro, 1592U89 did not demonstrate any mutagenic activity in the Ames Salmonella/mammalian-microsome mutagenicity assay.

Similarly, no significant *in vitro* toxicity in human bone marrow stem cells has been observed. In an in vitro assay for toxicity of human red blood cell precursors, the IC_{50} value of 1592U89 (100M) was 300-fold greater than that of zidovudine.

In CD-1 mice given 1592U89 succinate orally for 28 to 30 days, findings were limited to reversible increases in serum triglycerides and cholesterol at 330 and 1000 mg/kg/day and reversible increase in liver weight at 1000 mg/kg/day only. Similar increases in serum triglycerides (140 and 420 mg/kg/day) and increased liver weight (420 mg/kg/day only) were

noted in cynomolgus monkeys given 1592U89 succinate orally for 28 days. Lower doses in mice (110 mg/kg) and monkeys (50 mg/kg) had no demonstrable effects. In 90 day oral toxicology studies performed in female cynomolgus monkeys, findings were limited to reversible increases in liver weight in the highest dose treatment group (420 mg/kg/day), with no histopathological or ultra structural changes observed. CD-1 mice were given up to 1000 mg/kg/day in a 90 day oral toxicology study. Effects were again limited to the liver in mice receiving 330 and 1000 mg/kg/day and included increases in cholesterol, alanine aminotransferase (ALT) and triglycerides. Hepatocellular hypertrophy, apparently due to an increase in smooth endoplasmic reticulum, bile stasis and individual cell necrosis were also seen. There were no demonstrable effects at the 110 mg/kg dosing level.

Results from the six and twelve month toxicology studies are now available in our updated version of the clinical investigators brochure dated 14th March 1997.

1.3.4 *Clinical Studies*

1.3.4.1 *Study 131-001*

The initial Phase I study was a randomised, double-blind, placebo-controlled, parallel group, dose-escalation trial, which evaluated the safety and pharmacokinetics of single oral doses of 1592U89. Eighteen HIV-infected subjects (15 males and 3 females), with a mean CD4+ count of 338 cells/mm^3 (range: 10 to 713 cells/mm^3), each received five single escalating doses of 1592U89 (100, 300, 600, 900 and 1200 mg) or placebo. Subjects were randomly assigned to receive either 1592U89 (n = 12) or placebo (n = 6), with successive doses separated by an interval of at least 7 days. At the 300 mg dose level (3 x 100 mg 1592U89 tablets), subjects were additionally randomised to receive their dose either in a fasted state or following a standardised meal to evaluate the effect of food on the bioavailability of 1592U89. All other doses were administered in the fasted state. Six subjects were also asked to return at the end of the study to receive an additional 300 mg dose of 1592U89 in the form of an oral solution to evaluate the relative bioavailability of the 1592U89 tablets. Serial blood and urine samples were collected prior to each dose and thereafter at pre-specified intervals up to 24 hours post-dosing for determination of the plasma and urine concentrations of 1592U89 by a validated high performance liquid chromatography (HPLC) method. Subjects were closely monitored for the occurrence of adverse experiences (AEs).

The mean plasma concentration-time profiles of 1592U89 for each dose group (n=8) are presented in Figure 1.

Figure 1. Mean (± SE) Plasma 1592U89 Concentration vs. Time Curves Following Single, Oral Doses of 100, 300, 600, 900 and 1200 mg Under Either Fasted or Fed Conditions

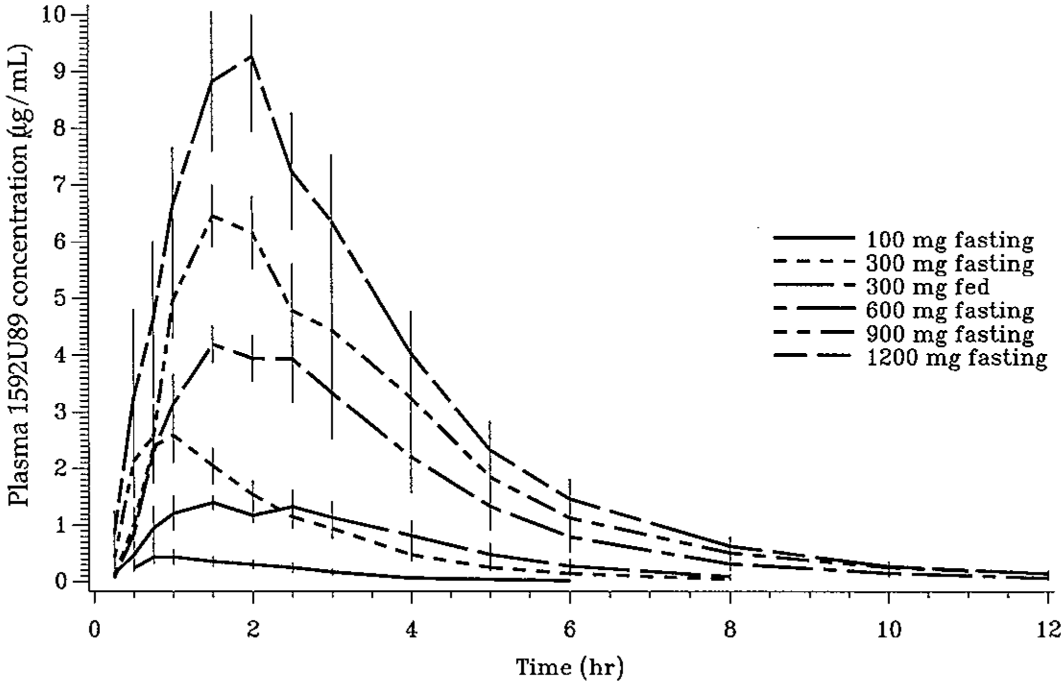

Study 131–001: N=8, Same Subjects

Individual and group mean pharmacokinetic parameters were calculated for each dose group, using model-independent methods and are summarised in Table 1.

Table 1. Mean (%CV) Pharmacokinetic Parameters for 1592U89 Following Single, Oral Doses of 100, 300, 600, 900 and 1200 mg Under Fasting Conditions (n=8)

Parameter (units)	Dose of 1592U89									
	100 mg		300 mg		600 mg		900 mg		1200 mg	
$AUC_{0\rightarrow\infty}$ (hr·µg/mL)	1.09	(65)	5.83	(49)	15.86	(48)	25.27	(47)	33.12	(42)
CL/F (mL/min/kg)	29.3	(62)	13.8	(41)	10.1	(41)	9.3	(34)	9.2	(30)
C_{max} (µg/mL)	0.59	(57)	2.80	(48)	4.72	(31)	8.10	(32)	9.61	(33)
t_{max} (hr)	1.26	(45)	1.03	(30)	1.71	(44)	1.60	(33)	1.56	(27)
$t_{½}$ (hr)	0.88	(20)	1.14	(16)	1.42	(14)	1.41	(15)	1.50	(13)

At the 300 mg dose, the tablet AUC was 99% of the oral solution AUC. Administration with food lowered the tablet AUC by 5% and C_{max} by 35%, compared to administration under fasted conditions. 1592U89 was safe and well tolerated by all subjects, with few treatment-emergent adverse experiences reported, primarily GI disturbances. Four subjects withdrew for reasons unrelated to drug.

1.3.4.2 *Study CNAA2001 (Previously 131-002)*

Study CNAA2001 is currently ongoing. This study was designed to evaluate the safety, tolerance and pharmacokinetics of 1592U89 when administered alone and in combination with ZDV. In this Phase I/II, multi-centre, multiple-dose escalation trial, 80 HIV-infected subjects without AIDS, are enrolled sequentially into each of four dosing cohorts (20 subjects per cohort). The study will ultimately examine the following dosages of 1592U89: 200, 400 and 600 mg, TID and 300 mg, BID. All four dosages are expected to achieve plasma concentrations above the *in vitro* IC_{50} for HIV-1. In each dosing cohort, subjects receive 1592U89 alone for the first four weeks of the study. At the beginning of the fifth week, subjects within each dose cohort are randomised to receive, under double-blind conditions, either ZDV or matched placebo, at the same dosing frequency as the 1592U89, for an additional eight weeks. Escalation to higher dose cohorts only occurs once the previous dosing cohort is fully accrued and at least 10 subjects have completed a full four weeks of treatment.

Preliminary blinded results obtained from the first dose cohort (1592U89 200 mg TID for 4 weeks followed by the addition of AZT/Placebo for a further 8 weeks) show a marked response in both the CD4+ cells and the HIV RNA viral load, measured by the Roche assay (Table 2).

Table 2. Immunological and Virological Responses – Preliminary Results of 1st Cohort – 1592U89 200 mg TID

	1592U89		1592U89 + ZDV/Placebo	
	**Baseline**	**Week 4**	**Week 8**	**Week 12**
Median CD4	352	435 (+80)	474 (+112)	449 (+90)
Median log viral load	5.11	3.37 (-1.8)	3.02 (-2.1)	3.22 (-2.1)

__Note:__ Numbers in parentheses represent median of changes from baseline n=19

Monthly safety reviews have shown 1592U89 to be well tolerated. In the first cohort (200 mg tid), the more commonly reported adverse experiences (regardless of attributability) included nausea, headache, asthenia, rash, fever, diarrhoea, dyspepsia, stomatitis, depression, insomnia, paresthesia and pruritus among others. One patient had to discontinue treatment as he presented with fever, rash, vomiting and diarrhoea which recurred on re-challenge, compatible with a drug allergic reaction. The same patient showed a reversible grade 3 elevation of ALT. One serious adverse experience was reported. This was a hospitalization for 24 hours due to a severe headache which was attributed to the lumbar puncture performed earlier that day.

In this study CSF samples were obtained from four individuals in the first cohort from 1.5 to 2.0 hours following dose administration. The 1592U89

concentrations in CSF averaged 0.14 µg/mL (0.5 µM) with a CV of 10% (range: 0.13 to 0.16 µg/mL). This 1592U89 CSF concentration (0.5 µM) compares favourably with the IC_{50} for 1592U89 in clinical isolates (0.26 µM). The CSF/plasma ratios of 1592U89 concentrations averaged 0.18 with a CV of 35% (range: 0.10 to 0.25). Due to the changing 1592U89 concentrations in both the CSF and the plasma, the CSF/plasma ratios are at best only an estimate of the CSF penetration of 1592U89. The only situation in which a single CSF/plasma ratio gives a reliable measure of CSF penetration is when a constant-rate infusion has achieved constant concentrations in both CSF and plasma. Balis et al (30) have investigated the CSF/plasma ratio of zidovudine under these conditions and they found it to be 0.24±0.09.

A more detailed review of the *in vitro* antiretroviral activity and all preclinical and clinical, pharmacological, pharmacokinetic and toxicological data is contained within the Investigator's Brochure.

1.3.4.3 *Clinical Experience in nRTI-Experienced Adults*

There has been very limited experience with the use of 1592 in patients previously treated with other anti-retroviral agents; especially nucleoside reverse transcriptase inhibitors (nRTI). Fifty two (52) patients who participated in CNAA2001 (a phase 2 dose-escalating study of 1592 and AZT/1592 in treatment naive adults) and discontinued 1592 therapy after completing the 12 week treatment phase of the protocol have reinstated 1592 therapy after a 1-6 month interim (treatment discontinuation required until adequate pre-clinical safety data were available to support longer term treatment in humans). During this interim, patients were encouraged to receive alternative anti-retroviral therapy with commercially available agents. Preliminary data are available from 15 patients who re-started 1592 treatment in addition to continuing their other anti-retroviral agents. The type of interim anti-retroviral treatment which these patients received was quite variable with the most common treatment chosen (6 patients) being AZT/3TC. At week 4 after re-starting 1592 treatment, 14 of these 15 patients (93%) had plasma HIV RNA below the limits of detection of the assay (400 c/ml; Roche Amplicor). At week 12 after re-starting 1592 treatment, 8 of 9 patients (89%) had plasma HIV RNA below the limits of detection of the assay. These data suggest that patients treated with other ant-retroviral agents for period of up to 6 months continue to experience additional anti-retroviral activity when 1592 is added to their current treatment regimen.

A phase II study (protocol CNAA2003) is currently being conducted to evaluate the anti-retroviral effect of 1592 in patients who have received long-term nRTI therapy and are currently failing this treatment regimen (e.g. plasma HIV RNA >10,000 c/ml). This population was studied to assess the effect of 1592 in patients who probably harbor nRTI-resistant virus (e.g. resistant to one or more nRTI). Forty patients were intended to be enrolled in this protocol, however, enrollment was discontinued after 33 patients had been enrolled due to difficulties in identifying patients who met the strict inclusion criteria regarding prior treatment history. Patients were eligible for

enrollment if they met one of the following treatment history criteria: 1) ≥6 months d4T monotherapy, 2) ≥6 months ddI (±ZDV) therapy, 3) ≥12 months ZDV monotherapy, or 4) ≥12 months ZDV/3TC therapy. These prior treatments were chosen to assure that the patient population studied would include a broad distribution of nRTI experience and potential resistance. All patients received 1592 300 mg BID for up to 24 weeks in addition to their current anti-retroviral therapy.

Preliminary virology data from 23 patients in this study are available for the first 4 weeks of treatment with 1592. Median baseline plasma viral load in these 23 patients was 4.70 $\log_{10}$ c/ml (range 3.07 to 5.83 $\log_{10}$ c/ml) Addition of 1592 to current treatment in these patients resulted in an additional median 1.02 $\log_{10}$ c/ml decrease in plasma HIV RNA (range 0.42 increase to -3.15 decrease) at week 4 of treatment. While this median decrease in plasma HIV RNA in this patient population is quite impressive, especially for a patient population that was failing their prior treatment regimen, a detailed analysis of individual patient data indicated a variable response to 1592 treatment. For example, 10 of 23 patients (43%) had a decrease in plasma HIV RNA of <0.5 $\log_{10}$ c/ml. However, 5 of 23 patients (21%) had a decrease in plasma HIV RNA to below the limits of detection of the assay (<400 c/ml; Roche Amplicor assay). While analysis of virologic genotypic and phenotypic data and viral load changes from this study are still incomplete, it appears that the patients in this study with prior long term exposure to multiple (≥2) nRTI therapies (and who are failing treatment probably due to the development of resistance to these agents as implied by a high viral load) tended to exhibit an attenuated response to the addition of 1592 to existing therapy. Viral isolates from these patients demonstrated multiple genotype changes associated with resistance to a number of nRTI. The implications of these data are not currently clear although it suggests that patients who develop viral resistance to multiple nRTI may exhibit only limited anti-retroviral response to 1592 when administered alone. This study does not give us any information on the contribution of 1592 in combination with other new agents (agents that the patient has never received) in which other factors such as synergy may play a role in the activity of the new regimen. A more detailed analysis of the data from this study will be conducted after the completion of the study. Also, additional studies are planned or ongoing to better define the benefit of 1592 treatment in nRTI-experienced patients.

---

2. OBJECTIVES

2.1 Primary Objectives

- To evaluate the benefit of adding 1592U89 to current antiretroviral therapies in AIDS dementia complex patients as determined by performance in standardised neuropsychological tests
- To assess the safety and tolerance of the treatment regimens in subjects with AIDS dementia complex.

2.2 Secondary Objectives

- To determine the effect of regimens containing 1592U89 on :

  Clinical dementia status determined by changes of stage of the Memorial Sloan Kettering (MSK) rating scale

  Neurological Status

  Survival

  Occurrence of other AIDS-defining conditions

  Viral load, both in CSF and peripheral blood, as measured by HIV RNA PCR

  CSF surrogate markers -β_2 microglobulin, quinolinic acid and TNF receptor-2.

  Viral mutations in the RT gene in plasma and CSF

  CD4+ cell count

  Self-assessed health status as measured by the medical outcome study (MOS) questionnaire

  Extent of assistance required with activities of daily living as measured by the HIV-Caregiver Impact Assessment (HIV-CIA)

  Characterisation of steady state plasma and CSF concentrations of 1592U89 in patients with AIDS dementia complex.

---

3. STUDY DESIGN

This is a phase III randomised, double-blind, multicentre study to evaluate the safety and efficacy of 1592U89 in patients with AIDS dementia complex. Patients may continue to receive other antiretroviral agents which are approved for marketing in at least one country. Agents available by expanded access or other parallel track programs will be assessed on a case-by-case basis with the sponsor.

Patients will be pre-stratified into groups A or B depending on whether their existing therapy contains zidovudine or not. Each stratum will then be centrally randomised separately to receive 600 mg of 1592U89 or placebo every twelve hours. Study medication will be administered in addition to the patient's existing antiretroviral therapy which may include a combination of agents. Patients will be required to be stable on their current antiretroviral treatment for a minimum of 8 weeks prior to study entry. Dose increase or addition of background antiretroviral agents will not be permitted during the 8 week pre-entry period or the randomised phase of the study.

The study will be comprised of a 12 week randomised phase followed by an optional open phase of 40 weeks when open label 1592U89 will be offered provided that no unacceptable toxicities are observed in this or other studies involving 1592U89.

Study participants who progress on treatment or experience severe antiretroviral drug toxicity not related to 1592U89 may be offered to commence the open-label phase of the study provided a minimum of 6 weeks on study have been completed.

Patients will be defined as having progressed on study treatment if their ADC clinical status deteriorates by at least one stage on the MSK rating scale (ie. from stage 1 to 2 or stage 2 to 3). To confirm such deterioration is caused by HIV-1, any CNS opportunistic infections or other conditions will be excluded by:

- MRI scan (or CT scan with contrast if MRI scanner facilities are not available to the site)
- CSF analysis (as detailed in Appendix F2) and/or
- Any other required supporting tests

In addition, these patients will need to be evaluated on a weekly basis until the dementia deterioration is confirmed to be caused by HIV-1.

The end-point committee will validate all the clinical dementia progressions occurring in the study. Requests to commence the study's open phase prior to week 12 will require the approval of the end-point committee.

All patients will be required to complete a follow-up assessment within 4 to 6 weeks of receiving the last dose of 1592U89.

Clinical end-points not related to ADC will also be validated on an ongoing basis by an external physician.

3.1 Dose Rationale

The dose for 1592U89 in this study will be 600 mg every twelve hours. Effective viral suppression and CSF penetration has been observed with the lower dose of 200 mg every eight hours (section 1.3.4.2). However given the good safety profile observed to date and in the belief that a higher dose may increase the good blood-brain barrier penetration already observed with 1592U89, it has been agreed that the chosen dose would provide a balanced level of safety and efficacy.

---

4. TRIAL SUBJECT SELECTION

4.1 Inclusion Criteria

A. Male or female

B. Participants must be ≥ 18 and ≤ 65 years of age

C. HIV-Infection documented by one or more of the following methods:

- Licensed ELISA / Western Blot detection of HIV antibody OR
- Positive HIV blood culture OR
- Positive p24 HIV serum antigen OR
- Quantitative HIV RNA PCR

D. Patients must have evidence of AIDS Dementia Complex as defined by the AAN (American Academy of Neurology) (Appendix B2). Patients fulfilling ADC stage 1 criteria as defined by the MSK scale will also be eligible to enrol into this study (Appendix B1).

E. Patients will be required to be impaired by 1.5 Standard Deviations below normal in at least two neuropsychological domains from the battery described in Appendix D.

F. Patients must have been stable on their current antiretroviral treatment for a minimum of 8 weeks prior to study entry.

G. Women of childbearing potential who have a negative pregnancy test (serum β-HCG) within 14 days of the start of dosing. Both male and female study subjects must use an adequate and reliable form of contraception starting one month prior to entry and throughout the study period.

H. Ability to provide written informed consent by the patient or legal guardian (subject to local regulatory and IRB/Ethics Committees regulations/guidelines).

4.2 Exclusion criteria

A. Patients so debilitated as a result of their HIV disease or associated illness or therapies who, in the investigator's opinion, will not reasonably be expected to complete the 12 week randomised dosing period.

B. Patients who have an MSK (Memorial Sloan Kettering) rating scale score of dementia severity of ≥ 3.

C. Patients with a Karnofsky score <60 (Appendix C)

D. Patients with confounding neurological disease which may interfere with interpretation of neurological and neuropsychological assessments including:

   i. Patients with previous neurological disease unrelated to HIV infection: multiple sclerosis, documented stroke, degenerative

disease. Patients with chronic seizure disorders or head injury will only be excluded if the condition results in functional impairment or is likely to interfere with the evaluation. Patients who have been stable for at least 2 months or are on maintenance anticonvulsant therapy following a seizure may be enrolled after discussion with the sponsor.

ii. Patients who have current CNS infections or neoplasms as revealed by MRI scan (CT scan with contrast if MRI scanner facilities not available) and CSF analysis (as detailed in appendix F2).

Note: Patients who have been previously treated for cerebral toxoplasmosis or tuberculous, cryptococcal or other fungal meningitis and are clinically stable, may be enrolled after obtaining approval from the sponsor and the clinical end-point committee. In addition, CMV retinitis patients on chronic maintenance may be enrolled in the study after discussion with the sponsor.

E. Patients with current alcohol or illicit drug use which, in the opinion of the investigator, may interfere with the patient's ability to comply with the study protocol.

F. Patients with a history of clinically-relevant hepatitis within the last 6 months.

G. Patients with a history of clinically-relevant pancreatitis in the last 6 months

H. Patients with a malabsorption syndrome or other gastrointestinal dysfunction which renders them unable to take oral medication.

I. Patients with the following laboratory test results within 14 days of study entry/drug administration

- Hemaglobin[1]<10 g/dl
- Neutrophil count[2] <1000 cells/mm^3
- Platelet count <75,000 cells/mm^3
- AST or ALT >5 times the upper limit of normal
- Estimated creatinine clearance <50 ml/min

  Estimated creatinine clearance should be calculated using the

---

NO TAG
Transfusions and/or erythropoietin may be used in order to reach or maintain this value
NO TAG
G-CSF use is permitted to reach or maintain this value

following formula:

$$\frac{(140\text{–age})(\text{weight in kg})}{\text{serum creatinine x 72}} \text{ x (0.85 for women only)}$$

- Amylase >1.5 upper limit of normal

J. Pregnant women or women who are breast feeding (subject to local regulatory and IRB/Ethics Committees regulations/guidelines)

K. Treatment with radiation therapy or cytotoxic chemotherapeutic agents within one month of entry, or an anticipated need for such treatment within the next 12 weeks (with the exception of local treatment for Kaposi's sarcoma)

L. Patients who, within the past 3 months, have participated in an investigational antiretroviral trial or received a dose of HIV vaccine.

M. Treatment with immunomodulating agents such as systemic corticosteroids, interleukins, thalidomide, anti-cytokine agents, anti-oxidants or interferons within one month of study entry.

N. Patients who have received or have an anticipated need for the use of nerve growth factor within the next 12 weeks.

O. Patients that during the trial require the use of narcotic analgesics, sedatives, benzodiazepines, antidepressants, anti- psychotic agents and other psychoactive drugs which in the opinion of the investigator would interfere with the assessment of the neurological and neuropsychological status of the patient.

P. Patients suffering from chronic diseases such as diabetes, congestive heart failure, cardiomyopathy, other cardiac dysfunctions, etc, which, in the opinion of the investigator, would compromise the safety of the patient.

Q. Patients with symptomatic AIDS-defining opportunistic infection not responsive to therapy e.g. Pneumocystis carinii pneumonia, Mycobacterium avium complex.

4.3 Number and Source of Subjects

A minimum of 90 subjects will be enrolled in the study. Subjects who are enrolled from centres in which the standardized neuropsychological tests have not been validated will not contribute to the 90 subjects needed to compare neuropsychological profiles of the treatment groups. The study will be conducted at study centres in Australia, the United States, Europe and Canada.

4.4 Subject Stratification and Randomisation

Subjects will be stratified during the pre-entry period into group A if their existing therapy contains zidovudine or into group B if they are not taking

zidovudine in their antiretroviral regimen. Subjects receiving d4T will be stratified into group B.

Randomization will be done centrally through the GW study co-ordinators either in the US or the UK depending on whether the sites are in the US/Canada or Europe/Australia.

Each stratum will be randomised separately to ensure a good distribution of active/placebo treatment.

4.5 Subject Enrolment

Once patients have been screened and confirmed to be eligible for enrolment, the following enrollment procedures should be followed:

1. The site will complete a patient enrollment form and URGENTLY fax or E-mail the GW study coordinator confirming patients details of stratification and eligibility criteria.
2. The patient will be randomised and assigned a treatment number. This information will be faxed back to the site and the local GW monitor.
3. The CTM centre will dispatch by courier the randomised supply of 1592U89 direct to the site or the local GW operating company as appropriate.

Given the wide geographic distribution of sites, care should be taken not to incur unnecessary delays in any of the above procedures in order to ensure CTM delivery on time for the study start.

---

5. CLINICAL TRIAL MATERIAL

5.1 Study Drugs

1592U89 will be supplied by Glaxo Wellcome in bottles of 100mg and bottles of 70 tablets of 300mg. The 300mg formulation will be provided for open supplies only, whilst the randomised treatment will continue to be provided as 100mg tablets. To facilitate the switch of the two formulations, patients will be asked to finish their existing 100mg tablets supplies. The patient's information document will be amended to reflect this change, however, site personnel responsible for the dispensing of study drug, must ensure that both the patient and the patient's caregiver are fully informed of this change in dose administration to avoid any risk of overdose.

Study medication must be stored in a secured area with restricted access, between 15-25°C (59-77°F) and protected from light and moisture.

5.2 Supply of CTM to the Study Site

For the randomised phase of the study, 1592U89 will be distributed from the GW European Clinical Trial Supplies Centre based at Stevenage, UK.

Dispatch will be triggered by the receipt of a patient enrolment form by the GW study coordinator. The estimated timelines for the receipt of drug at the study site are 5-10 days depending on the location of the site.

5.3 Dose Regimens

Study participants will be randomised within each stratum as described below to receive either 1592U89 or placebo in addition to their current antiretroviral therapy for the first 12 weeks of the study. At the end of the randomised phase or at the time of ADC progression, or severe antiretroviral drug toxicity not related to 1592U89, there will be the option of continuing the study further for 40 weeks receiving open label 1592U89 (see section 3).

Treatment Stratum	1592U89/PLO	Background Antiretrovirals
A	600 mg bid	Existing therapy that includes zidovudine
B	600 mg bid	Existing therapy that does not include zidovudine

In the event of suspected drug toxicity dose modification procedures as outlined in section 6.9.3 must be followed.

5.4 Clinical Trial Material (CTM) Management

5.4.1 *Dispensing of CTM*

Medication will be dispensed by the designated study pharmacist or study co-ordinator under the principal investigator's authorisation.

For the initial 12 week randomised phase of the study, sufficient study medication for two weeks at a time will be dispensed to the patient. Once the open phase of the study is started, sufficient medication for eight weeks will be provided in order to coincide with study visits.

Study personnel will be responsible for maintaining accurate records of all clinical trial material received from Glaxo Wellcome, the dispensing to the patient and return of unused study medication.

5.4.2 *Disposition of CTM*

All medication not utilized during the study will be appropriately recorded and returned to Glaxo Wellcome. The study monitor will regularly review all CTM records.

5.5 Concomitant Medication

Subjects should take as few concomitant medications as medically feasible. All prescribed medication or blood products administered during the study must be carefully recorded on the case report form (CRF).

Immunomodulating agents such as systemic corticosteroids, interleukins, thalidomide, anti-cytokine agents, or interferons, cytotoxic chemotherapeutic agents, anti-oxidants and radiation therapy (with the exception of local KS treatment) must not be used throughout the randomised phase of the study.

Concomitant use of narcotic analgesics, sedatives, benzodiazepines, antidepressants, anti- psychotic agents and other psychoactive drugs which in the opinion of the investigator would interfere with the assessment of the neurological and neuropsychological status of the patient will not be permitted.

1592U89 has been observed to bind to human plasma proteins (49.4%±0.9). The observed binding was independent of concentration. Given the moderate extent of binding in human plasma at expected therapeutic concentrations, drug interactions mediated by protein binding displacement are not expected.

In humans, the 5′-carboxylic acid and the 5′-glucuronide are the major metabolites measured in plasma. Preliminary results from a single dose study in adults indicates that approximately 35% of a 300mg dose of 1592 is eliminated in the urine as the carboxylic acid metabolite and 26% as the 1592U89-glucuronide metabolite. Less than 3% is excreted unchanged in the urine, and oxidative metabolites of 1592U89 account for minor biotransformation products. *In vitro* experiments indicate that cystolic alcohol dehydrogenase (ADH) and UDP-glucuronyl transferase (UDPGT) are the enzymes responsible for the formation of the 5′-carboxylic acid and the 5′-glucuronide metabolites of 1592U89, respectively. At a clinically relevant concentration, *in vitro* experiments reveal that 1592U89 is not a substrate nor does it inhibit CYP3A4 activity.

To date, three formal drug interaction studies have been initiated for 1592U89. A single-dose drug interaction study has been conducted for 1592U89 in combination with ZDV and 3TC. No clinically significant interactions were observed and the study is summarized in the Investigator's Brochure. An ethanol interaction study has concluded its clinical phase and samples are being assayed. The third study is a methadone interaction study which has just begun enrolment. Other larger scale clinical studies are collecting population pharmacokinetic samples to screen for drug interactions affecting 1592U89.

Based upon the known metabolism of 1592U89, the following table has been constructed and highlights potential drug-drug interactions. Compounds with the potential for interaction with 1592U89 are identified as co-substrates or inducers of the ADH and UDPGT metabolic pathways. Because 1592U89 metabolism predominately occurs by two distinct pathways, and thus an alternate metabolic route is available, the potential for an increase in 1592U89 concentration is less likely when concomitantly administered with a co-substrate for one metabolic pathway. However, an increase in plasma concentration of the co-substrate may result upon concomitant administration with 1592U89. The scientific literature on the metabolism of the following

UDPGT substrates is being searched to determine whether or not UDPGT acts on the parent substrate or on a secondary metabolite. If it acts on the parent substrate then there is a potential for increases in the parent concentrations while if it acts on the secondary metabolite then the potential increases would be in the metabolite concentrations. Compounds known to induce UDPGT enzymes may cause the plasma concentrations of 1592U89 to decrease and thereby compromise the optimal therapeutic effect.

POTENTIAL INCREASE IN CONCENTRATIONS OF A CONCOMITANT MEDICATION (CO-SUBSTRATES)					
ADH SUBSTRATES		UDPGT SUBSTRATES			
alcohol	isoniazid	imipramine	chlorpromazine	lamotrigine	morphine
chlorzoxazone	chloral hydrate	amitriptyline	promethazine	valproic acid	dapsone
chlorpromazine		doxepin	chlorpheniramine	acetaminophen	propofol
		propranolol	labetalol	NSAIDS	temazepam
		oxazepam	naloxone	bumetanide	ketoconazole
		miconazole	fluconazole		
POTENTIAL DECREASE IN 1592U89 CONCENTRATIONS (UDPGT INDUCERS)					
phenytoin	clofibrate	phenobarbital	cigarette smoke	isoniazid	T3-thyroid hormone

- If a patient is taking one of the above co-therapies in the co-substrate categories and is not experiencing an adverse reaction or change in a pre-existing adverse reaction, then there is no reason to alter therapy. If, on the other hand, a patient is experiencing an increase in adverse reactions with the co-therapy, then a reduction in the dose of the co-therapy should be considered.

- If a patient is taking one of the above co-therapies in the inducer category, then the potential interaction would be to decrease 1592U89 exposure and potentially, efficacy.

To date, no specific contra-indications to 1592U89 have been identified. The listing above is produced in the interest of completeness to highlight potential interferences that could theoretically occur. In this light, it is advised to exercise cautious monitoring when co-administering drugs known to cause liver toxicity or induce myelosuppression.

---

6. MEASUREMENTS AND EVALUATIONS

Central Laboratories will be appointed to test the following parametres : serum chemistries, haematology, lymphocytes subsets, screening specialised tests such as VDRL, HIV-1 and pregnancy. The central laboratories will also be responsible for the preparation of PBMCs, shipment and storage of all frozen samples from the study.

Sites will be expected to carry out the screening CSF analysis as well as the processing and temporary storage at -70°C of subsequent CSF and plasma samples for pharmacokinetic, virology and marker analyses.

Note: CSF analyses during this trial will provide key information on the penetration and activity of 1592U89, therefore CSF sampling will be strongly encouraged. However, patients will not be refused enrolment or excluded from the study if they refuse to undergo a lumbar puncture that is not required for the exclusion of CNS opportunistic infections or cancers.

6.1 Pre-Entry Evaluations

After determining that a patient is eligible for enrolment, the following pre-entry evaluations should be completed within 14 days of study drug administration. However, given the nature of the population under study and the possible side effects experienced from the lumbar puncture at day -7, screening evaluations performed within 21 days of entry may be accepted after discussion with the sponsor.

Note: written informed consent must be obtained from each patient or legal guardian prior to initiation of pre-entry evaluation.

Day -14

Clinical Evaluations

- Demography
- Medical history
- History of neurological symptoms
- Neurological and neuropsychological assessments (to be used as baseline)
- ADC rating (MSK scale)
- Karnofsky Score
- Concurrent AIDS-defining indicator diseases/secondary infections
- Computed Tomography or Magnetic Resonance Imaging

Laboratory Evaluations

- Haematology
- Serum chemistries
- Serum β-HCG for females of childbearing potential
- Dipstick urinalysis
- VDRL (venereal disease research laboratory)

- HBsAg and anti-HCV antibody
- Anti-HIV-1 antibody

Day -7

Laboratory Evaluations

Note: CSF sampling on this visit is mandatory.

- CSF analysis: protein, glucose, cell counts, cell differential, VDRL, cryptococcal antigen, and fungal culture.
- CSF storage for markers analysis : β_2-microglobulin, quinolinic acid and TNF receptor 2
- CSF storage for virology analysis : HIV RNA PCR and genotype characterisation
- Plasma storage for virology analysis: HIV RNA PCR and genotype characterisation

6.2 On-Study Evaluations (Randomised phase)

During the randomised phase of the study patients will be evaluated in the clinic during Baseline and study weeks 2, 4, 6, 8 and 12.

Note : Baseline (Day 0) is the day on which the first dose of study drug is administered.

Clinical Evaluations

- Height (Baseline)
- Body weight (Baseline, weeks 4, 8, and 12)
- Vital signs (Baseline, weeks 2, 4, 6, 8, and 12)
- Neurological and neuropsychological assessments (weeks 6 and 12)
- ADC rating (MSK scale) (weeks 6 and 12)
- Karnofsky Score (Week 12)
- Concomitant medication recording (throughout the study period)
- Concurrent antiretroviral treatment recording (throughout the study period)
- Study drug recording (throughout the study period)

- Adverse experiences (throughout the study period)
- Concurrent medical conditions (AIDS-defining indicator diseases/secondary infections) (throughout the study period)
- Self-assessed health status (MOS questionnaire) (Baseline, weeks 6 and 12)
- HIV Caregiver impact assessment (Baseline, weeks 6 and 12)
- Hospitalisation/Inpatient Resource Utilization (throughout the study period)

Laboratory Evaluations

- Haematology (Baseline, weeks 2, 4, 6, 8 and 12)
- Serum chemistries (Baseline, weeks 2, 4, 6, 8 and 12)
- Dipstick urinalysis (Baseline, weeks 2, 4, 6, 8 and 12)
- Lymphocyte subset (Baseline, weeks 2, 4, 6, 8 and 12)
- Plasma storage for virology analysis - HIV RNA PCR and genotype characterisation, (Baseline, weeks 2, 4, 6, 8 and 12)
- PBMC processing and storage - SI/NSI phenotype (Baseline and week 12)
- CSF storage for virology analysis - HIV RNA PCR and genotype characterisation (weeks 6 and 12) (see note in section 6)
- CSF storage for markers analysis -β_2 microglobulin, quinolinic acid, and TNF receptor 2 (weeks 6 and 12) - (see note in section 6)
- Pharmacokinetic sampling and storage of

  - Plasma (Weeks 6 & 12)
  - CSF (Weeks 6 and/or 12) (see note in section 6)

6.3 Open Phase Evaluations

Patients will be offered to continue in the study's open phase after week 12 or prior to that if treatment failure and/or antiretroviral drug toxicity not associated with 1592U89 are confirmed. For the latter, a week 12 evaluation should be conducted once the decision has been made to enter the open phase and prior to commencing open treatment. Both groups may then be assessed during the open phase as follows:

Clinical Evaluations

- Body weight (weeks 14, 16, 20, 28, 36, 44, and 52)

- Vital signs (weeks 14, 16, 20, 28, 36, 44, and 52)
- Neurological and neuropsychological assessments (weeks 20, 28, 36, 44, and 52)
- ADC rating (MSK scale) (weeks 20, 28, 36, 44, and 52)
- Karnofsky Score (week 52)
- Summarised Concomitant medication recording (throughout the study period)
- Concurrent antiretroviral treatment recording (throughout the study period)
- Study drug recording (throughout the study period)
- Adverse experiences (throughout the study period)
- Concurrent medical conditions (AIDS-defining indicator diseases/secondary infections) (throughout the study period)
- Self-assessed health status (MOS questionnaire) (weeks 20, 28, 36, 44, and 52)
- HIV Caregiver impact assessment (weeks 20, 28, 36, 44, and 52)
- Hospitalisation/Inpatient Resource Utilization (throughout the study period)

Laboratory Evaluations

- Haematology (weeks 14, 16, 20, 28, 36, 44, and 52)
- Serum chemistries (weeks 14, 16, 20, 28, 36, 44, and 52)
- Dipstick urinalysis (weeks 14, 16, 20, 28, 36, 44, and 52)
- Lymphocyte subset (weeks 16, 20, 28, 36, 44, and 52)
- Plasma storage for virology analysis - HIV RNA PCR and genotype characterisation, (weeks 16, 20, 28, 36, 44, and 52)
- PBMC processing & storage - SI/NSI phenotype (weeks 36, and 52)
- CSF storage for virology analysis - HIV RNA PCR and genotype characterisation (weeks 20, 36 & 52) (see note in section 6)
- CSF storage for markers analysis -β_2 microglobulin, quinolinic acid, and TNF receptor 2 (weeks 20, 36 and 52) - (see note in section 6)

6.4 Extra-Assessment Evaluations (Unscheduled Visits)

Additional assessments may be performed throughout the study if deemed necessary by the investigator e.g. ADC progression and adverse experiences. The following evaluations should be conducted:

Clinical Evaluations

- Body weight
- Vital signs
- Concomitant medication assessment
- Adverse experiences
- Concurrent medical conditions (AIDS-defining indicator diseases/secondary infections)

The following additional assessments should be performed in the case of suspected ADC progression :

- Neurological and neuropsychological assessments
- ADC rating (MSK scale)
- Magnetic Resonance Imaging Scan (or CT scan with contrast if MRI scanner facilities not available)

Laboratory Evaluations

- Haematology
- Serum chemistries
- Dipstick urinalysis
- Exclusion Diagnosis CSF Analysis (as detailed in appendix F2)

NOTE: Should CSF sampling be required to diagnose any other CNS conditions, a 0.5 ml sample for pharmacokinetic analysis may be taken and stored with the patient's permission.

6.5 Last Visit Evaluation (Premature Discontinuation)

For patients who wish or need to withdraw from the study at any stage before the end of the study, the last study visit should be performed as specified for weeks 12 or 52, depending on whether the patient is on the randomised or open phase of the study at the time of premature discontinuation. In addition, a follow-up visit as described in section 6.6 must also be performed within 4-6 weeks after the last dose of study drug.

6.6 Follow-up Evaluation (Off-drug)

For those patients who decline to enter the open phase of the study or discontinue prematurely from the study at any stage, a follow-up visit should be performed between 4 to 6 weeks after the last dose of study drug.

The following evaluations should be performed:

Clinical Evaluation

- Neurological and neuropsychological assessments
- ADC rating (MSK scale)
- Karnofsky Score
- Body weight
- Vital signs
- Concomitant medication assessment recording
- Concurrent antiretroviral treatment recording
- Adverse experiences
- Concurrent medical conditions (AIDS-defining indicator diseases/secondary infections)
- Self-assessed health status (MOS questionnaire)
- HIV Caregiver impact assessment

Laboratory Evaluations

- Serum β-HCG testing for females of childbearing potential
- Haematology
- Serum chemistries
- Dipstick urinalysis
- Lymphocyte subset
- Plasma storage for virology analysis - HIV RNA PCR and genotype characterisation.

6.7 Pharmacokinetic Evaluations

Population pharmacokinetics will be conducted with CSF and plasma concentration data obtained from this study. The CSF samples will be

collected from a subset of patients at weeks 6 and/or 12. CSF samples will be optional.

Patients will be asked to attend the clinic to receive a supervised dose of study drugs on those days when pharmacokinetic samples are to be taken.

6.7.1 *Collection of Pharmacokinetic Samples*

A detailed description of the collection, labelling, processing and shipment of plasma and CSF samples is located in Appendix H.

6.7.2 *Collection of Plasma with Corresponding CSF Samples*

6.7.2.1 *CSF Samples*

CSF samples will be obtained on a voluntary basis in a subset of patients at week 6 and/or week 12. These patients will be sequentially assigned at each site to one of the following time-frames for CSF sample collection:

0.5-1 hours post-dose

1-2 hours post-dose

2-3 hours post-dose

3-4 hours post-dose

Each CSF sample should have a matching plasma sample taken at the same time point (Table 4).

Sites should attempt to obtain CSF samples from different patients, so that the set of samples obtained at each site is distributed equally across the four time frames.

Table 3. CSF Sample Time–Frame Assignments

Patient No	CSF Samples Hours post-dose			
	0.5-1	1-2	2-3	3-4

For each CSF sample, obtain 0.5ml of CSF and place in a biofreeze tube. Record the date and actual sample time on the case report form. Label each

tube with the bar-coded labels provided by Glaxo Wellcome. Upon collection, CSF samples should be frozen upright at -70°C until shipped.

For overall CSF volume requirements please see Appendix G section 1.3.2.

6.7.2.2 *Plasma Samples*

At weeks 6 and 12, three blood samples will be collected from every patient, one prior to the dose administration and the other two separated in time by approximately two hours. All samples should be collected within 0.5 to 4 hours post-dose.

Study dose must be supervised by site personnel on these visit days.

If the patient consents to have CSF taken, the timing of plasma samples will be dependent upon the assigned time of CSF sampling. Thus, one blood sample will be collected at the same time as the CSF sample and the other will be taken either two hours before or two hours after the CSF sample, depending on which time-frame the CSF sample is to be drawn. For example:

> If the CSF sample is to be drawn during the 0.5-1 hr or the 1-2 hour post-dose time-frames, one blood sample would be obtained simultaneously with the CSF sample and the other should be collected during the 2.5-3 or the 3-4 hour post dose time-frame as appropriate.
>
> If the CSF sample is to be taken at 3 hours post-dose, one blood sample would be drawn at the same time as the CSF sample but the additional blood sample would have been taken two hours prior to the CSF at 1 hour post-dose.

Table 4. CSF/Plasma Samples Coordinated Time Frames

CSF Samples	Plasma Samples	
Hours post-dose	Sample	Hours post-dose
0.5 - 1	1	Pre-dose
	2	0.5 - 1
	3	2.5 - 3
1 - 2	1	Pre-dose
	2	1 - 2
	3	3 - 4
2.5 - 3	1	Pre-dose
	2	0.5 - 1
	3	2.5 - 3
3 - 4	1	Pre-dose
	2	1 - 2
	3	3 - 4

Should the patient not consent to providing CSF, the plasma samples will still be collected and sites should attempt to obtain these samples across a range of time-frames, in order to obtain a good spread of concentration-time data which will facilitate the population pharmacokinetic modelling.

Table 5. Plasma Samples Time Frame Assignments

	Plasma Samples (Hours post-dose)		
Week 6	Sample 1<br>Sample 2<br>Sample 3	Pre-dose<br>0.5 - 1<br>2.5 - 3	
Week 12	Sample 1<br>Sample 2<br>Sample 3		Pre-dose<br>1 - 2<br>3 - 4

For each plasma sample, obtain 3ml of whole blood in a powdered EDTA containing tube. Record the date and actual sample time on the case report form. The plasma from each sample should be separated by centrifugation within one hour of blood collection, then stored in a biofreeze tube. Label each tube with the bar-coded labels provided by Glaxo Wellcome. Upon collection, plasma samples should be frozen upright at -70°C until shipped.

6.7.3 *Analytical Methodology*

Plasma and CSF concentrations of 1592U89 will be determined in all samples by a validated analytical method. Plasma and CSF concentrations of zidovudine and its glucuronide metabolite will be determined in all samples using a validated RIA method. Validation of the assay will include accuracy, precision, sensitivity and specificity.

6.8 Virology Evaluations

Quantitative plasma and CSF HIV-1 RNA PCR will be assessed by Glaxo Wellcome at the time points specified in sections 6.1 to 6.6. Viral genotype and phenotype will also be analysed at the most appropriate time points relevant at the time of analysis.

6.8.1 *Collection of Virology Samples*

A detailed description of the collection, labelling, processing and shipment of virology samples is located in Appendix G.

6.9 Adverse Event Reporting

One of the aims of this study is to assess the safety and tolerability of the drug and therefore the investigator is responsible for recording and reporting adverse events observed during (commencing from Day 1) and after drug treatment (up to the last follow-up visit in the study). In addition, serious

adverse events related to study participation only should be reported in the screening phase. If treatment for an adverse event is being considered, the investigator should bear in mind that the new treatment may obscure the results of study drug withdrawal or discontinuation (dechallenge), or cause further adverse events.

6.9.1 *Recording*

The investigator should record all adverse events (see definitions below) and pregnancies. At each visit, after the patient has had an opportunity spontaneously to mention any problems, the investigator should enquire about adverse events by asking the following standard questions:

- "Have you had any (other) medical problems since your last visit/assessment?
- Have you taken any new medication, other than those given to you within this study, since your last visit/assessment?"

6.9.2 *Documentation*

All adverse events and pregnancies must be promptly documented by completing the adverse event and pregnancy notification forms respectively supplied by Glaxo Wellcome. Their completeness and accuracy will be checked by the study monitor who visits the investigating centre.

6.9.3 *Definition of an Adverse Event (AE)*

An adverse event is defined as any untoward medical occurrence in a patient or clinical investigation subject administered a pharmaceutical product which does not necessarily have a causal relationship with this treatment. An adverse event (AE) can therefore be any unfavourable and unintended sign (that could include a clinically signficant abnormal laboratory finding), symptom, or disease temporally associated with the use of a medicinal product, whether or not considered related to the medicinal product.

An adverse event does include:

- An exacerbation of a pre-existing illness.
- Increase in frequency or intensity of a pre-existing episodic event or condition.
- A condition detected or diagnosed after trial medication administration even though it may have been present prior to the start of the study.
- Continuous persistent disease/symptoms present at baseline that worsen following the start of the study.

An adverse event does not include:

- Medical or surgical procedures (e.g., surgery, endoscopy, tooth extraction, transfusion); the condition that leads to the procedure is an AE.
- Pre-existing disease or conditions present or detected at the start of the study that do not worsen.
- Situations where an untoward medical occurrence has not occurred (e.g., hospitalisations for cosmetic elective surgery/social/convenience admissions).
- The disease being studied or sign/symptoms associated with the disease unless more severe than expected for the subject's condition.
- Overdose of either trial medication or concurrent medication without any signs or symptoms."

6.9.4 *Definition of a Serious Adverse Event (SAE)*

A serious adverse event includes any experience or event that is:

a. **fatal**

b. **life-threatening (except if clearly related to HIV disease as listed in Appendix N)**

A life-threatening event is present when the subject was, in the view of the investigator, at immediate risk of death as the event occurred.

This definition does not include an event that, had it occurred in a more serious form, might have caused death.

c. **disabling or incapacitating (except if clearly related to HIV disease as listed in Appendix N)**

d. **requires in-patient hospitalisation (except if clearly related to HIV disease as listed in Appendix N)**

Hospitalisation for elective treatment of a pre-existing condition that did not worsen during the study is **not** considered an adverse event.

Complications which occur during hospitalisation are adverse events and if a complication prolongs hospitalisation then the event is serious.

e. **a congenital anomaly in the offspring of a patient who received drug (except if clearly related to HIV disease as listed in Appendix N)**

f. **cancer (except if clearly related to HIV disease as listed in Appendix N)**

g. **an event resulting from an overdose of the study drug**

Overdose is defined as the ingestion of at or above 1200mg/dose of the study drug (1592U89).

h. **an event which, though not included in (a) through (g) above, may jeopardise the patient or may require intervention to prevent one of the outcomes listed in (a) to (g), (except if clearly related to HIV disease as listed in Appendix N).**

i. **all grade 4 laboratory abnormalities, regardless of symptomatology are regarded as serious adverse events**

Laboratory abnormalities < grade 4 toxicity will not automatically be considered serious. However, if they meet any other of the SAE criteria they should be reported as SAEs.

6.9.4.1 *Project-specific Exemptions from the AE and SAE Definition*

The following events are not to be considered AEs or SAEs:

- **any condition which occurred prior to the patient being enrolled in the study and which did NOT worsen during the study**

These conditions should be recorded at screening.

The following HIV-related events are to be excluded from the definition of adverse events and serious adverse events and all of the associated reporting time frames (unless they are fatal), even if there is reason for their being classified as serious (e.g. hospitalisation, life-threatening etc):

- **any condition that is clearly related to HIV disease as listed in Appendix N. Lymphomas and invasive cervical carcinoma are excluded from this exemption; they must be reported even if they are considered to be HIV-related.**

Any of the HIV-related conditions which appear in Appendix N should be recorded on the "HIV-associated conditions" page in the CRF.

Fatal HIV-related events are not part of this exemption; they should be recorded and reported according to the criteria for SAEs.

6.9.4.2 *Reporting*

Life-threatening adverse events and deaths, whether causally related to treatment or not, must initially be notified by the investigator to Glaxo Wellcome within 24 hours. All data immediately available must be reported, preferably by completing and faxing the adverse event forms.

Telephone notification is required in the absence of facsimile facilities. Following the initial notification, the investigator must then record all

additional information and fax or mail a copy of the completed SAE reporting forms within 48 hours.

For all other serious adverse events (see definition), the Serious adverse event form must be fully completed and a copy returned within 48 hours.

6.9.4.3 *Other Investigations*

The investigator and others responsible for patient care should institute any supplementary investigations of serious adverse events based on their clinical judgement of the likely causative factors. This may include extra laboratory tests, histopathological examinations, or seeking a further opinion from a specialist in the field of the adverse event. Glaxo Wellcome may also request extra tests or extra follow-up information from the trialist. If a patient dies, any post-mortem findings including histopathology must be provided to Glaxo Wellcome.

Pregnancies must be notified by the investigator to the Glaxo Wellcome monitor within two weeks of the investigator learning of the pregnancy.

Glaxo Wellcome will provide a Pregnancy Notification Form to collect the required data. Women who become pregnant must discontinue from the study."

6.9.4.4 *Pregnancy Reporting*

Pregnancies must be notified by the investigator to the Glaxo Wellcome monitor within two weeks of the investigator learning of the pregnancy.

Glaxo Wellcome will provide a Pregnancy Notification Form to collect the required data. Women who become pregnant must discontinue from the study.

6.9.4.5 *Regulatory Aspects*

Glaxo Wellcome has a legal responsibility to notify both the local regulatory authority and other overseas agencies about the safety of a drug under clinical investigation. Prompt notification of serious adverse events to the Glaxo Wellcome staff concerned, by the investigator, is essential so that legal obligations and ethical responsibilities towards the safety of other patients are met.

If there is difficulty in contacting the Glaxo Wellcome Personnel responsible for the study, information can be relayed through the Head of Clinical Studies Safety Management in International Product Safety & Pharmacovigilance, UK (0)181 422 3434.

In agreeing to the provisions of this protocol, the investigator accepts responsibility from prompt notification of SAEs to Glaxo Wellcome.

6.9.5 *Dose Modification due to Toxicity or Adverse Experience*

Adverse experiences which occur during the trial should be evaluated by the investigator and graded according to the ACTG toxicity scales (see Appendix J).

Study treatment dosing may be modified at the discretion of the investigator and according to the severity of the adverse experience. All dose modifications must be accurately recorded on the study drug pages of the case report forms. If treatment for an adverse event is being considered the investigator should bear in mind that the new treatment may obscure the results of study drug withdrawal or discontinuation (dechallenge), or cause further adverse events.

Should the investigator have evidence that the adverse experience is not caused by study medication, dosage may then remain unaltered. However, if the attributability of the AE cannot be ascertained dose modifications should be instigated.

Subjects requiring a dose modification must be re-evaluated on a weekly basis.

6.9.5.1 *Grade 1Toxicity/Adverse Experience*

Subjects who develop a grade 1 adverse experience may continue therapy without alteration of the study treatment.

6.9.5.2 *Grade 2Toxicity/Adverse Experience*

Subjects who develop a grade 2 adverse experience may continue therapy at the discretion of the investigator.

6.9.5.3 *Grade 3Toxicity/Adverse Experience*

Subjects who develop a grade 3 adverse experience should have their study medication withheld until the AE diminishes to ≤ grade 1 for one week. At that time, subject may be re-challenged with 50% of study dosing. If the AE remains at ≤ grade 1 for two weeks, full treatment may be reinstated.

Should the same grade 3 adverse experience recur within 4 weeks in the same subject, the study medication should be permanently discontinued. Patients experiencing recurrence of haematological toxicity may remain on study at the discretion of the investigator and under strict clinical management.

Note : The aetiology of neuropathies may be difficult to ascertain, therefore study treatment should initially be withheld. Should the neuropathy not improve within one week, study treatment may be reinstated at the discretion of the investigator. The end-point committee must be notified.

6.9.5.4 *Grade 4Toxicity/Adverse Experience*

Subjects who develop a grade 4 adverse experience should have their study medication permanently discontinued. The subject should be followed

weekly until resolution of the adverse experience and encouraged to complete the protocolled study evaluations. Grade 4 neuropathies may be handled as above.

6.9.5.5 *Handling of Rash/Allergic Reaction Episodes*

Rash associated with systemic symptoms (eg. fever, nausea, vomiting) has been reported in approximately 4% of patients treated with 1592U89 which recurs within a few hours of rechallenge. Subjects who develop a rash in association with systemic symptoms should have all study medications interrupted. Subjects must not be rechallenged with 1592U89 without prior discussion with the sponsor. When the symptoms have resolved, subjects may be rechallenged with background antiretrovirals at the discretion of the investigator.

6.10 Neuropsychological Evaluations

Neuropsychological performance will be the primary end-point of the study. The tests that will be used in this study include the Rey Auditory Verbal Learning Test Trials I-V and Delayed Recall, Grooved Pegboard (dominant and non-dominant), Trail Making A and B, Symbol Digit, Cal Cap Reaction Time and Verbal Fluency (see Appendix D).

At pre-entry patients will be assessed with each neuropsychological test and the raw scores will be compared against standardised look-up tables which have been adjusted for age and education. The scores in the tables correspond to performance of 1.5 Standard Deviation below the reference mean for seronegative controls. A patient will be considered to have sufficient impairment for study entry if the performance is below those given in the tables on two or more tests. There will be one standardised look-up table provided for each test in the CRF for visit day -14.

The standardised tables have been constructed from data accumulated from a group of uninfected (HIV-1 seronegative) homosexual and bisexual men enrolled in the multicentre-AIDS Cohort Study (MACS) (31). These data have been kindly provided for reproduction and use in this study by Dr. Justin McArthur and Dr. Ola Selnes from the Johns Hopkins University, Baltimore.

For the remaining of the study, raw scores will be collected and entered into the database. Neuropsychological performance will be assessed from the changes observed in the "Z" score within the individual. "Z" scores will be calculated at the analytical stage by the GW project statistician using normative data from the mentioned cohort(31).

In order to standardise the administration of the neuropsychological tests, training and detailed instructions (written and videotaped) will be provided to the participating neuropsychologists.

Copyrighted tests will be purchased by GW and be provided together with the remaining tests at the back of the CRF. They will be used as source

documentation and will not be entered into the database. A summary score sheet will be placed within the main CRF for entry into the database.

6.11 Neurological Evaluations

Neurological status will be evaluated using the assessments used by the AIDS Clinical Trials Group (for further detail please consult Appendix E and the CRF).

The neurological subscore will be calculated and used to assess the ADC stage of the patient as defined by the MSK scale.

Neurological evaluations will be kept at the back of the CRF and will not be entered into the database; they will be used as source documentation. A summary score sheet will be placed within the main CRF for entry into the database.

6.12 ADC rating (MSK scale)

The MSK scale will be used to assess the ADC status and progress of the patient. In order to standardise and provide a guide to participating investigators, a neurological subscore range has been assigned to stages 1, 2 and 3 of the MSK scale as follows:

Stage 1 will encompass patients with a neurological score between 4 and 6

Stage 2 will encompass patients with a neurological score between 7 and 9

Stage 3 will encompass patients with a neurological score between 10 and 12.

Clinical judgement will prevail and cases in which the neurological score may disagree with the stage assigned should be documented to the end-point committee who will ultimately be responsible for the validation of all the clinical dementia end-points in the study.

6.13 Validation of Dementia End-Points

For the purpose of this protocol, a negative dementia end-point is defined as progression by at least one stage within the MSK scale for ADC. At the time the subject is considered to have met a negative end-point, an exclusion diagnosis, confirmed by MRI Scan, CSF analysis and any other supporting documentation will be required.

Given the subjectivity involved in the assessment and staging of ADC, an end-point committee comprised by four participating investigators (see attached appendix K), has been established to independently review all dementia end-points occurred during the study. In addition to the validation of negative end-points, improvement by at least one stage within the MSK scale will also require validation by the end-point committee. For the

validation of improvement, a clinical rationale, which will include a brief description of the improvement observed by the physician, caregiver and/or participating subject, will be submitted to the end-point committee. To ensure an independent assessment, the origin of the site or investigator producing the end-point, will not be revealed to the committee. Similarly, any end-points arising from the site of any of the committee members will not be reviewed by the member in question.

The validation of the dementia end-points will be co-ordinated through the GW study co-ordinator. Further details of the process for confirmation of end-points can be found in the study procedures manual.

6.14 Validation of HIV Clinical End-Points

Systemic clinical end-points will be analysed as part of the secondary objectives of this study. All AIDS defining events occurring during both phases of the study (randomised and open), will be validated by an external expert physician.

A section of the study procedures manual will be dedicated to define standardised criteria which must be met for the diagnosis of all AIDS events which develop during the study. In addition, a list of the diagnostic procedures which should be used, and the documentation which should be available to support the unequivocal diagnosis of a particular AIDS event, will be provided. Please refer to the procedures manuals for further details.

6.15 Patient and Care Given Impact Evaluations

6.15.1 *Patient Health Status Questionnaires (MOS-HIV)*

In addition to motor and cognitive impairment evaluated with neuropsychological tests, patients with ADC experience impaired ability to perform normal daily activities. These functional limitations, as well as impaired social functioning associated with flattened affect or depressed mood are likely to result in impaired quality of life or perceived poor health status. Lack of self-awareness seen in ADC patients with MSK scores of 2 or 3 may prevent patients from recognizing the extent of their impairment. However, it is not clear at what stage self-awareness is so diminished patients are no longer able to reliably report on their own health status or quality of life.

To examine how patients with ADC at varying stages evaluate their functioning and quality of life, all patients in this trial will be asked to complete questionnaires containing the Medical Outcomes Study Health Status Questionnaire for HIV (MOS-HIV; NO TAG). See Appendix L for sample questionnaires.

All patients will be asked to complete questionnaires during clinic visits at weeks 0, 6, 12 and study discontinuation. The investigator will ask the study

patient to complete a questionnaire during the clinic visit after the routine patient history has been taken and after the standard adverse event questions (See Section 6.9) have been answered.

6.15.2 *HIV Caregiver Impact Assessment (HIV-CIA)*

Providing help to a patient with ADC may have significant impact on the psychological, emotional, physical and economic well-being of the caregiver. If a therapy can effectively reduce the extent of patient dependence on a caregiver, the caregiver may be more productive, less prone to illness, and better able to cope with the challenges of ADC.

Most patients rely on other people for social support to cope with their HIV infection. Supporters or "caregivers" may be friends, family members, buddies, partners or health professionals (e.g., home healthcare providers, case workers, counsellors). Caregivers may provide any or all of the following forms of support: affection, friendship, compassionate listening, transportation, help with household chores, economic assistance, nursing care or aid with basic self-care.

To identify the patient's primary caregiver, the investigator will ask the patient to identify the person on whom they most rely for help in coping with their HIV infection (including providing a telephone number and address which can be used to contact the caregiver). The patient then will be asked to bring the person they named with them to some study visits during the course of the trial. If the patient has no one on whom he/she relies for help or support but has an assigned case worker or home healthcare worker, the investigator will request that the patient authorize the study coordinator to send the caregiver impact assessment to the patient's case worker/home healthcare worker to complete.

If the caregiver is unable to come to the clinic to complete caregiver assessments, the study coordinator will mail the questionnaire with a prepaid, preaddressed return envelope to the caregiver to complete and return to the clinic.

6.15.3 *Questionnaire Translations Available*

The following translations of patient and caregiver questionnaires will be available for this study:

Countries	MOS-HIV	HIV Caregiver Impact Assessment
The Netherlands	Dutch	English
Australia	English	English
UK	English	English
US & Canada	English<br>Spanish	English

6.15.4 *Administration of Questionnaires*

Questionnaires should be completed in a quiet place (preferably the same place, if possible), and at as consistent a time of day as possible according to the protocol. Generally, to avoid biasing responses to the instrument, respondents (patients for MOS-HIV; caregiver for HIV-CIA) should not be told the results of diagnostic tests prior to completing the questionnaire. Regardless of when the questionnaire is completed, the respondent should be given adequate time to complete all items. No stated or implied time limit for completing the questionnaire will be given.

The investigator will complete header information on the respondent's questionnaire, then read the instructions aloud to the patient. If the patient/caregiver refuses to complete the questionnaire, the Investigator will ask him/her to initial and date the questionnaire cover in the space indicating that he/she declined the opportunity to complete the questionnaire.

The investigator should ask the respondent to complete the questionnaires as completely and as accurately as possible. If the respondent is physically or visually impaired, the investigator may read aloud the questionnaire and/or record the respondents answers but the investigator should not interpret a question or response for the patient or caregiver.

If the respondent requests clarification of any question or assistance in interpreting a question or response option, he or she should be asked to read the instructions again and to give the best answer possible to each question. Patients and caregivers should be encouraged that it is their experiences and opinions that are requested. Respondents may use a dictionary to clarify unfamiliar words. The investigator will not provide respondents with an answer to any question nor interpret any portion of a question for a respondent.

---

7. DATA MANAGEMENT AND ANALYSIS

7.1 Case Report Form

Case report forms (CRFs) will be provided for each patient by Glaxo Wellcome. They will be supplied as NCR paper, the top copy will be collected

by the sponsor on a regular basis and the bottom copy will be left at the investigative site. CRF completion guidelines will also be provided prior to the start of the study.

7.2 Sample Size

A sample size of 45 patients per treatment group will be required in order to provide 90% power to detect a difference between treatment groups of 0.6 in the change from baseline of summary neuropsychological Z scores at Week 12. This sample size assumes alpha = 0.05, standard deviation = 0.75 and a drop-out rate of 20-25%.

7.3 Statistical Analysis

Analyses will be performed by the Department of Clinical Statistics, Glaxo Wellcome. A detailed analysis plan will be formulated according to company procedures to describe the statistical analyses proposed for this trial.

Data from all subjects enrolled in the study will be summarized. If protocol violations are numerous, a preferred analysis of the efficacy variables may be performed.

The evaluation of safety and antiretroviral activity of 1592U89 will be presented in summary and/or graphical form. This will include adverse experience reports, clinical laboratory analyses, virological and immunological markers. Other data will be collected to examine neurological evaluations, AIDS-defining conditions, and survival.

The primary efficacy measure will be the comparison of the changes in the standardised neuropsychological tests between standard therapy (i.e. zidovudine for the stratum A or current approved antiretroviral therapy for the stratum B) with and without concurrent 1592U89 therapy. Study subjects who are enrolled from centres in which the standardized neuropsychological test have not been validated will be excluded from this analysis.

Treatment groups will be compared using the non-parametric stratified Wilcoxon rank-sum test on the change from baseline to week 12 of the summary "Z" score.

The summary Z score will be computed as follows:

1. Convert all individual test scores to a Z score by subtracting the reference group mean and dividing by the reference group standard deviation.
2. Take the average of all individual Z scores for each patient

Each individual test will also be compared for illustrative purposes.

Because the drop-out rate is expected to be at or above 20%, the same analyses described above will also be performed using a Last Observation

Carried Forward approach. For patients who do not have a Week 12 summary measure, the Week 6 summary measure (or the baseline value if Week 6 is missing) will be carried forward as the Week 12 result.

The attrition rate at Week 12 will be evaluated using Fisher's Exact Test.

The change of dementia clinical stage as measured by the MSK rating scale will be evaluated by the time to increase of at least one stage. The distribution of the time to each event will be estimated using the Kaplan-Meier product-limit survival method.

Data from this protocol may be pooled with data from other protocols to perform a meta analysis exploring the treatment effect on clinical progression or death. The meta analysis, stratified by protocol, would compare 1592U89 -containing regimens against regimens not containing 1592U89 with respect to the incidence of new CDC class B or C event/death and the distribution of the time to event/death. This analysis could provide supportive information for demonstrating the clinical benefit of 1592U89.

Safety analyses will examine adverse experiences, clinical laboratory and vital signs over time. Data will be presented in summary and/or graphical form by strata and treatment group.

7.4 Pharmacoeconomic Analysis

Pharmacoeconomic analyses will compare treatment groups using non-parametric stratified Wilcoxon rank-sum tests on the change from baseline to week 12 in MOS-HIV (Medical Outcome Study-HIV) and HIV-CIA (HIV-Caregiver Impact Assessment) scale scores. Analyses will compare treatment groups on change in :

- MOS-HIV

  - Mental Health summary scale scores
  - Physical Health summary scores

- HIV-CIA

  - ADL support scores

A last observation carried forward analysis also will be conducted on these measures to estimate the impact of the expected 20% drop-out rate for this trial. This analysis will carry forward the last questionnaire completed by the patient or caregiver as the last observation for change score analyses.

Treatment differences in procedure costs associated with unscheduled visits or hospitalisations will be examined using non-parametric analysis of variance. Costs for procedures will be assigned based on average costs for each procedure reported in the Maryland Medicaid database for the most recent 3 year period.

8. STUDY MANAGEMENT

8.1 Approval and Consent

8.1.1 *Approval from Regulatory Authorities*

This protocol will be submitted to the FDA under IND 45,331 prior to initiation of the study. All investigative sites participating in the study will be registered with the FDA, under form 1572.

The study will not commence in a particular country until the appropriate approval is obtained from the necessary regulatory authorities.

8.1.2 *Ethical Approval*

The protocol must be reviewed and approved by the appropriate local or national Ethics Committees (EC)/IRB for each study centre. In addition, the principal investigator must submit a sample copy of the Informed Consent Form and the Patient Information Sheet, if appropriate, to the local EC/IRB for their review and approval. A copy of the EC/IRB approval of both the protocol and Informed Consent form/Patient Information Sheet must be forwarded to the study sponsor prior to initiation of the study.

8.1.3 *Informed Consent*

It is anticipated that all patients eligible for this study will retain the ability to provide initial written consent. Therefore informed written consent from each participant or legal guardian must be obtained BEFORE any protocol mandated procedures are performed. Should the dementia status of the patient deteriorate during the course of the study, the legal/named guardian may revoke any permission previously granted by the participant. A copy of the signed Informed Consent Form must be retained by the patient and the original must be retained by the principal investigator.

All women of childbearing age must be informed of the unknown risk of study drug to the developing embryo or fetus.

Patient confidentiality will be strictly maintained. No patient names or contact addresses may be disclosed. All patients will be identified by means of initials and patient study number only. These details should be included on all CRFs, laboratory samples and source documents which are forwarded to the sponsor.

Patients will be informed by the Investigator of any relevant advances in antiretroviral therapies as they become available.

8.2 Patient Withdrawal or Discontinuation

Patients will be terminated from the study for any of the following reasons:

- Any adverse reaction deemed sufficiently serious to require discontinuation of therapy.
- Women who become pregnant.
- Patient non-compliance.

  All study participants are required to adhere to the protocol evaluation schedule. Failure to adhere with this schedule without having first provided justification to the investigator may result in the participant being withdrawn from the study.

  Patients are required, at each visit, to return all partially used or unused packs of study medication and to report all episodes of non-compliance with dosing schedules, without prejudice. Patients should, as far as is possible, also keep records of concurrent medications taken between scheduled visits.

  Patients must not give study medication to any other person or agency. Participants found to be in violation of the above will be withdrawn from the study.

- Investigator non-compliance.

  Glaxo Wellcome reserves the right to discontinue study centres for significant deviations from the protocol without prior approval by the sponsor and regulatory authorities. The principal investigator at each site is responsible for the accuracy and completeness of al research records, ensuring appropriate clinical and laboratory evaluations are conducted as outlined in the protocol, and that study drugs are appropriately stored, dispensed and accounted for.

- At the request of the patient or investigator without prejudice to future healthcare.
- At the request of the sponsor with reasonable cause.
- Progression of disease which, in the opinion of the principal investigator, should preclude further study.
- Intolerance to study drugs at doses indicated in the protocol.
- If the subject requires cytotoxic chemotherapy or radiation therapy (with the exception of local treatment for Kaposi's Sarcoma)
- If the subject requires another investigational drug deemed unacceptable by the principal investigator.

8.3 Study or Site Closure

The sponsor reserves the right to terminate the study or a site at any time, giving a period of 30 days notice to the investigator. Upon receipt of this

notice to terminate, the investigator will be expected to take all reasonable steps necessary to terminate the study as soon as possible.

8.4 Study Monitoring

Monitoring visits by sponsor personnel will be made on a frequent basis with advanced notification to study site personnel. Scheduling of such visits will be in line with the requirements of the sponsor.

8.5 Source Document Verification

Source document verification will be carried out in accordance with sponsor policy. The investigator must agree to allow access for sponsor personnel to study documents and relevant hospital/clinic records for confirmation of data throughout the study period and at any time thereafter in line with regulatory requirements.

8.6 Audit

This study may be subject to audit by the sponsor and/or regulatory authorities. Under such circumstances, the investigator must agree to allow access to study documents and relevant hospital/clinic records.

8.7 Records Retention

The investigator must arrange for the retention of study records at the site. The nature of the records and the duration of the retention period must meet the requirements of the relevant regulatory authorities.

8.8 Documentation and Publication

A detailed report of the study for internal use and possible submission to regulatory authorities will be prepared. There will be a publication committee appointed for the preparation of a manuscript for publication. The committee will be coordinated by the study chair and sponsor. Authorship of the manuscript will be considered by the committee and will be based on the contributions to the study and preparation of the manuscript. No other publications, either in writing or verbally, will be made before the definitive manuscript has been agreed upon and accepted for publication, without prior approval of the Sponsor.

8.9 Compensation for Injury

Glaxo Wellcome subscribes to the guidelines on compensation to patients for injuries incurred in clinical trials as issued by ABPI, January 1991.

In those countries where similar compensation is provided under local legislation or regulation or other appropriate arrangements, the above will not apply and appropriate local procedures will be followed.

9. EXTENSION PHASE

9.1 Objectives

1. To assess long term safety and tolerance of 1592U89 in subjects with AIDS Dementia Complex
2. To evaluate survival

9.2 Rationale

Safety updates on the use of 1592U89 in clinical trials is regularly reported to the FDA. These data suggest that 1592U89 is generally well tolerated with an acceptable adverse experience and laboratory toxicity profile.

GW is committed to provide access to 1592U89 treatment to those subjects who have come to the end of the CNAB3001 study and continue to benefit from 1592U89. Extended therapy beyond 52 weeks is therefore warranted.

9.3 Study Design

This amendment provides a means to prolong access to 1592U89 treatment to those study participants who continue to benefit from 1592U89. Enrolment into this extension phase will only be offered to those subjects who successfully complete 52 weeks in the CNAB3001 study. Prior to enrolment patients will provide informed consent incorporating the provisions of this treatment extension phase. This study is extended until 1592U82 becomes commercially available. Subjects will be monitored every 12 weeks for safety and limited efficacy evaluations. Neurological assessments will be performed whenever a subject's clinical dementia status is suspected to have deteriorated. End-point validation will not be required in this part of the study.

9.4 Subject Selection

9.4.1 *Inclusion Criteria*

a. Subjects must have participated in study CNAB3001.

b. Written informed consent which incorporates this protocol amendment.

c. Subjects must be considered by the investigator to derive benefit from continued therapy with 1592U89.

9.4.2 *Exclusion Criteria*

a. Any Subjects that permanently discontinued 1592U89 therapy during the original 52 weeks of this protocol are excluded.

9.5 Concomitant Medications

There are no restrictions on the use of concomitant medication in this part of the study, however, attention is drawn to those compounds listed in section 5.5 to minimise the risk of any drug interaction.

9.6 Clinical Trial Material

1592U89 will be supplied as described in Section 5.1. Study drug will be shipped to study centres by Glaxo Wellcome. Accurate drug accountability records will be maintained by study personnel.

9.6.1 *Dose & Regimens*

Subjects will continue to receive 1592U89 600mg twice a day (approximately every 12 hours). Toxicity management guidelines are described in section 6.9.5. These guidelines are to be used at the discretion of the investigator.

9.7 Measurements & Evaluations

9.7.1 *On Study*

<u>Laboratory Evaluations</u>

Haematology (Weeks 64, 76, 88 , 100 and 12 weekly thereafter)
Serum Chemistries (Weeks 64, 76, 88 , 100 and 12 weekly thereafter)
Lymphocyte Subset (Weeks 64, 76, 88, 100 and 12 weekly thereafter)
Plasma storage for virology analysis (Weeks 64, 76, 88, 100 100 and 12 weekly thereafter)
CSF storage for virology/inflammatory markers (optional)

<u>Clinical Evaluations</u>

ADC Rating (MSK scale) (Weeks 64, 76, 88 , 100 and 12 weekly thereafter)
Neurological assessment (as required)
HIV Associated Conditions (as required)
Adverse Event reporting (as required)
Study Drug Recording
Concomitant Medication (Summarised data)

9.7.2 *Study Discontinuation*

<u>Laboratory Evaluations</u>

Haematology
Serum Chemistries
Lymphocyte Subset
Plasma storage for virology analysis

CSF storage for virology/inflammatory markers (optional)

Clinical Evaluations

ADC Rating (MSK scale)
Neurological assessment
HIV Associated Conditions
Adverse Event reporting
Karnofsky Score
Study Drug Recording
Concomitant Medication

9.7.3 *Follow-up (Off-drug)*

A Follow-up evaluation should be scheduled at approximately 30 days following discontinuation of therapy with 1592U89

Laboratory Parameters

Haematology
Serum Chemistries
Lymphocyte Subset
Plasma storage for virology analysis
CSF storage for virology/inflamatory markers (optional)

Clinical Evaluations

ADC Rating (MSK scale)
Adverse Event reporting

REFERENCES

1. Schmid P, Conrad A, Syndulko K et al. Quantifying HIV-1 proviral DNA using the polymerase chain reaction on cerebrospinal fluid and blood of seropositive individuals with and without neurologic abnormalities. J Acquired Imm Def Synd 1994;7:777-788.

2. Davis LE, Hjelle BL, Moller VE et al. Early viral brain invasion in iatrogenic human immunodeficiency virus infection. Neurology 1992;42:1736-1739.

3. Dreyer EB, Kaiser PK, Offermann JT, Lipton SA. HIV-1 coat protein neurotoxicity prevented by calcium channel antagonists. Science 1990;248:364-7.

4. Heyes MP, Brew BJ, Martin A, et al. Quinolinic acid in cerebrospinal fluid and serum in HIV-1 infection: relationship to clinical and neurological status. Ann Neurol 1991;29:202-9.

5. Genis P, Jett M, Bernton EW, et al. Cytokines and arachidonic acid metabolites produced during human immunodeficiency virus (HIV)-infected macrophage-astroglia interactions: implications for the neuropathogenesis of HIV disease. J Exp Med 1992;176:1703-18.

6. Lipton SA, Rosenberg PA. Excitatory amino acids as a final common pathway for neurologic disorders. N Engl J Med 1994;330:613-22.

7. Lipton SA. Models of neuronal injury in AIDS: another role for the NMDA receptor? Trends Neurosci 1992;15:75-9.

8. Griffin DE, Wesselingh SL, McArthur JC. Elevated central nervous system prostaglandins in human immunodeficiency virus-associated dementia. Ann Neurol 1994;35:592-7.

9. Giulian D, Vaca K, Noonan CA. Secretion of neurotoxicity by mononuclear phagocytes infected with HIV-1. Science 1990;250:1593-6.

10. Navia BA, Cho E, Petitio CK, Price RW. The AIDS dementia complex II. Neuropathology. Ann Neurol 1986;19:525-535.

11. Navia BA, Jordan BD, Price RW. The AIDS dementia complex I. Clinical features. Ann Neurol 1986;19:517-524.

12. Everall IP, Luthert PJ, Lantos PL. Neuronal loss in the frontal cortex in HIV infection. Lancet 1991;337:1119-1121.

13. Ketzler S, Weis S, Haug H, et al. Loss of neurons in the frontal cortex in AIDS brains. Acta Neuropathol 1990;80:92-94.

14. Petito CK, Navia BA, Cho E-S, Jordan BD, George DC, Price RW. Vacuolar myelopathy pathologically resembling subacute combined

degeneration in patients with acquired immunodeficiency syndrome (AIDS). N Engl J Med 1985; 312:874-879.

15. Navia BA, Price RW. The acquired immunodeficiency syndrome dementia complex as the presenting or sole manifestation of human immunodeficiency virus infection. Arch Neurol 1987;44:65-69.

16. American Academy of Neurology AIDS Task Force. Report of a working group of the American Academy of Neurology AIDS Task Force. Neurology 1991;41:778-85.

17. Maj M. Organic mental disorders in HIV-1 infection. AIDS 1990;4:831-840.

18. Price RW, Brew BJ. The AIDS dementia complex. J Infect Dis 1988;158:1079-83.

19. Chiesi A, Vella S, Dally LG, Pedersen C, Danner S, Johnson AM, et al. Epidemiology of AIDS Dementia Complex in Europe. J of Acquir Immune Defic Synd & Hum Retrovirol 1996; 11:39-44

20. McArthur JC, Hoover DR, Bacellar H, Miller EN, Cohen BA, Becker JT et al. Dementia in AIDS patients: Incidence and risk factors. Neurology 1993;43:2245.

21. Portegies P, de Gans J, Lange JMA, Derix MMA, Speelman H, Bakker M et al. Declining incidence of AIDS dementia complex after introduction of zidovudine treatment. Br Med J 1989;299:819-821

22. Schmitt FA, Bigley JW, McKinnis R, Logue PE, Evans RW, Drucker JL et al. Neuropsychological outcome of zidovudine (AZT) treatment of patients with AIDS and AIDS-related complex. N Engl J Med 1988; 319;1573-1578.

23. Sidtis JJ, Gatsonis C, Price RW, Singer EJ, Collier AC, Richman DD, et al. Zidovudine Treatment of the AIDS Dementia Complex: Results of a Placebo-Controlled Trial. Ann Neurol. 1993;33:343-349

24. Tartaglione TA, Collier AC, Coombs RW, Opheim KE, Cummings DK, Mackay SR et al. AIDS Cerebrospinal fluid findings in patients before and during long-term oral zidovudine. Arch Neurol 1991;48:695-699

25. Pizzo PA, Eddy J, Falloon J, Balis FM, Murphy RF, Moss H, et al. Effect of continuous intravenous infusion of zidovudine (AZT) in children with symptomatic HIV infection. N Engl J Med 1988;319:889-896.

26. Brew BJ, Bhalla RB, Fleisher M, Paul M, Khan A, Schwartz MK, et al. Cerebrospinal fluid β_2 microglobulin in patients infected with human immunodeficiency virus. Neurology 1989;39:830-834.

27. Brew BJ, Dumbar N, Pemberton L, Kaldor J. Cerebrospinal fluid concentrations of β_2 microglobulin and neopterin predict the development of AIDS Dementia Complex. J Inf Dis (in press).

28. Brew BJ, Dumbar N, Pemberton L, Kaldor J. Elevated cerebrospinal fluid (CSF) concentrations of β_2 microglobulin and neopterin and CD4 cell count predict the development of AIDS Dementia Complex. Neuroscience of HIV Infection 96.

29. Mellors JW, Larder BA, Schinazi RF. Mutations in HIV-1 reverse transcriptase and protease associated with drug resistance. Int. Antiretroviral News 1995; 3:1;8-15

30. Balis FM, Pizzo PA, Murphy RF, et al. The pharmacokinetics of zidovudine (AZT) administered by continuous intravenous infusion in children. Ann Intern Med 1989; 110:279-285.

31. Selnes OA, Jacobson L, Machado AM, Becker JT, Wesch J, Miller E, et al. Normative data for a brief neuropsychological screening battery. Perceptual and Motor Skills 1991;73:539-550.

32. Wu AW, Rubin HR, Mathews WC, et al. A health status questionnaire using 30 items from the Medical Outcomes Study: preliminary validation in persons with early HIV infection. Medical Care, 1991;29:786-798.

33. de Boer JB, van Dam FSAM, Sprangers MAG. Health-related quality-of-life evaluation in HIV-infected Patients. Pharmacoeconomics 1995;8:291-304.

34. Scott-Lennox J, Adams JE, Lennox RD, Rubin M. Impact of 3TC+ZDV Therapy on Quality of Life in HIV Infected Patients with CD4 < 300. 35th Interscience Consortium on Antimicrobial Agents and Chemotherapy, San Francisco, California, September, 1995.

35. Scott-Lennox J, Adams JE, Lee JT, Lennox RD. Quality of life associated with zidovudine plus lamivudine combination treatment in therapy-naive HIV-infected patients. American Society for Microbiology's 3rd Conference on Retroviruses and Opportunistic Diseases, Washington, DC, January 1996.

36. Scott-Lennox J, Lee JT, Lennox JD. Sensitivity of health-related quality of life scores to adverse events in clinical trials. International Society for Quality of Life Research meeting, Montreal, October 1995.

37. Burgess A, Dayer M, Catalan J. The reliability and validity of two HIV-specific health-related quality-of-life questionnaires. AIDS 1993; 7:1001-1006.

38. Scott-Lennox J, Wu AW, Boyer JG, Collis PJ, Ware JE. A Multinational Psychometric Validation of the MOS HIV. 2nd Annual International Conference on AIDS Impact: Biopsychososical Aspects of HIV and AIDS, Brighton, England, July 1994.

Appendix A: STUDY FLOW CHART

Evaluation	Pre-Study		Randomised Phase						Open Phase							Follow Up NO TAG	Extra Assessments
			Baseline Day 0	Week					Week								
	-14	-7		2	4	6	8	12 NO TAG	14	16	20	28	36	44	52 NO TAG		
Informed Consent	✓																
Demography	✓																
Medical History	✓																
History of Neurological Symptoms	✓																
Height			✓														✓ NO TAG
Computed Tomography/Magnetic Resonance Imaging	✓																
Weight			✓		✓		✓	✓	✓	✓	✓	✓	✓	✓	✓	✓	✓
Vital Signs			✓	✓	✓	✓	✓	✓	✓	✓	✓	✓	✓	✓	✓	✓	✓
Neuropsychological Evaluation	✓					✓		✓			✓	✓	✓	✓	✓	✓	✓ NO TAG
Neurological Evaluation	✓					✓		✓			✓	✓	✓	✓	✓	✓	✓ NO TAG
ADC Rating (MSK Scale)	✓					✓		✓			✓	✓	✓	✓	✓	✓	✓ NO TAG
Karnofsky Score	✓					✓		✓							✓	✓	
HIV Caregiver Impact Assessment			✓			✓		✓			✓	✓	✓	✓	✓	✓	
Self-Assessed Health Status MOS Questionnaire			✓			✓		✓			✓	✓	✓	✓	✓	✓	
Hospitalisation/Inpatient Resource Utilization				✓-------											-------✓)		
Concurrent Medical Conditions	✓		(✓-------														-------✓)

Evaluation	Pre-Study		Randomised Phase						Open Phase							Follow Up[NO TAG]	Extra Assessments
			Baseline Day 0	Week					Week								
	-14	-7		2	4	6	8	12 NO TAG	14	16	20	28	36	44	52 NO TAG		
Concomitant Medication			(✓------														------✓)
Concurrent Anti-Retroviral Treatment			(✓------														------✓)
Adverse Experiences			(✓------														------✓)
Study Drug Record			(✓------												------✓)		
LABORATORY ASSESSMENTS																	
Serum β-HCG[NO TAG]	✓																
HIV-1 Ab	✓																
VDRL	✓																
HBsAg	✓																
HCV Ab	✓																
Haematology	✓		✓	✓	✓	✓	✓	✓	✓	✓	✓	✓	✓	✓	✓	✓	✓
Serum Chemistries	✓		✓	✓	✓	✓	✓	✓	✓	✓	✓	✓	✓	✓	✓	✓	✓
Lymphocyte Subsets			✓	✓	✓	✓	✓	✓		✓	✓	✓	✓	✓	✓	✓	
Dipstick Urinalysis[NO TAG]	✓		✓	✓	✓	✓	✓	✓	✓	✓	✓	✓	✓	✓	✓	✓	✓
Plasma Storage (HIV RNA PCR and Genotype)		✓	✓	✓	✓	✓	✓	✓		✓	✓	✓	✓	✓	✓	✓	✓[NO TAG]
PBMC Storage (SI/NSI Phenotype)			✓					✓					✓		✓		
CSF Analysis (Exclusion Diagnosis)		✓															✓[d]
CSF Storage: Markers Analysis[NO TAG]		✓				✓		✓			✓		✓		✓		✓[NO TAG]

Evaluation	Pre-Study		Randomised Phase						Open Phase							Follow Up[NO TAG]	Extra Assessments
			Baseline Day 0	Week					Week								
	-14	-7		2	4	6	8	12[NO TAG]	14	16	20	28	36	44	52[NO TAG]		
CSF Storage: Virological Analysis[NO TAG]		✓				✓		✓			✓		✓		✓		
Pharmacokinetic Sampling<br>Plasma Storage<br>CSF Storage[NO TAG]						✓		✓									✓[NO TAG]

[a] Not mandatory but strongly encouraged (see note in section 6).

[b] Women of childbearing age only.

[c] Dipstick for blood and protein.

[d] To be performed in cases of suspected ADC progression.

[e] If patient withdraws from the study prematurely before week 12 please complete CRF corresponding to visit 12.

[f] If patient withdraws from the open phase of the study prematurely i.e. before week 52 please complete week 52 assessment as the last study visit.

[g] Please complete follow-up assessment within 4-6 weeks after the last dose of 1592U89 was taken.

[h] To be performed if circumstances permit, in cases where a lumbar puncture is required as part of clinical management or end-point validation.

Appendix A1: Extension Phase Study Flow-Chart

Evaluation	Week No								
	52[b]	64	76	88	100	12 weekly	Discontinuation	Follow-up (off drug)	Extra Assessments
Informed Consent	✓								
ADC Rating (MSK Scale)		✓	✓	✓	✓	✓	✓	✓	✓
Neurological Assessment		(✓ - - -	- - -	- - -	- - -	- - -	- - -	- - -	- - - ✓)
Karnofsky Score							✓		
HIV Associated Conditions		(✓ - - -	- - -	- - -	- - -	- - -	- - -	- - -	- - - ✓)
Concomitant Medications		(✓ - - -	- - -	- - -	- - -	- - -	- - -	- - -	- - - ✓)
Study Drug Record		(✓ - - -	- - -	- - -	- - -	- - -	- - -	- - -	- - - ✓)
Adverse Experiences		(✓ - - -	- - -	- - -	- - -	- - -	- - -	- - -	- - - ✓)
LABORATORY PARAMETERS									
Serum β HCG								✓	
Haematology		✓	✓	✓	✓	✓	✓	✓	✓
Serum Chemistry		✓	✓	✓	✓	✓	✓	✓	✓
Lymphocyte Subsets		✓	✓	✓	✓	✓	✓	✓	✓
Plasma Storage (HIV RNA PCR & Genotype)		✓	✓	✓	✓	✓	✓	✓	✓
CSF Storage ([a]) (Virology & Markers)		✓	✓	✓	✓	✓	✓	✓	✓

[a] To be collected optionally and in cases where a lumbar puncture is required as part of clinical management

[b] Evaluations to be conducted as per Appendix A

Appendix B:
AIDS DEMENTIA COMPLEX RATING SCALES

Appendix B1: Memorial Sloan Kettering Rating Scale

STAGE 0 (normal	Normal mental and motor function.
STAGE 0.5 (equivocal/subclinical)	Absent, minimal or equivocal symptoms without impairment of work or capacity to perform activities of daily living (ADL). Mild signs (snout response, slowed ocular or extremity movements) may be present. Gait and strength are normal
STAGE 1 (mild)	Able to perform all but the demanding aspects of work or ADL but with unequivocal evidence (signs or symptoms that may include performance on neuropsychological testing) of functional intellectual or motor impairment. Can walk without assistance.
STAGE 2 (moderate)	Able to perform basic activities of self-care but cannot work or maintain the more demanding aspects of daily life. Ambulatory, but may require a single prop.
STAGE 3 (severe)	Major intellectual incapacity (cannot follow news or personal events, cannot sustain complex conversation, considerable slowing of all output or motor disability (cannot walk unassisted, requiring walker or personal support, usually with slowing and clumsiness of arms as well).
STAGE 4 (end stage)	Nearly vegetative intellectual and social comprehension and output are at a rudimentary level. Nearly or absolutely mute. Paraparetic or paraplegic with urinary and faecal incontinence.

Appendix B2: American Academy of Neurology Diagnosis of HIV-1 Associated Dementia Complex and Cognitive Impairment

Probable (must have **each** of the following)

1. a. Acquired abnormality in at least two of the following cognitive abilities (present for at least 1 month): attention/concentration, speed of processing of information, abstraction/reasoning, visuospatial skills, memory/learning, and speech/language. The decline should be verified by reliable history and mental status examination. In all cases, when possible, history should be obtained from an informant, and examination should be supplemented by neuropsychological testing.

   b. Cognitive dysfunction causing impairment of work or activities of daily living (objectively verifiable or by report of a key informant). This impairment should not be attributable solely to severe systemic illness.

2. At least **one** of the following:

   a. Acquired abnormality in motor function or performance verified by clinical examination (e.g., slowed rapid movements, abnormal gait, limb incoordination, hyperreflexia, hypertonia, or weakness), neuropsychological tests (e.g., fine motor speed, manual dexterity, perceptual motor skills), or both.

   b. Decline in motivation or emotional control or change in social behavior. This may be characterized by any of the following: change in personality with apathy, inertia, irritability, emotional lability, or new onset of impaired judgment, characterized by social inappropriate behavior or disinhibition.

3. Absence or clouding of consciousness during a period long enough to establish the presence of criterion 1. above.

4. Evidence of another etiology - including active CNS opportunistic infection or malignancy, psychiatric disorders (e.g., depressive disorder), active alcohol or substance use, or acute or chronic substance withdrawal - must be sought from history, physical and psychiatric examination, and appropriate laboratory and radiologic investigation (e.g., lumbar puncture, neuroimaging). If another potential etiology (e.g, major depression) is present, it is *not* the cause of the above cognitive, motor, or behavioral symptoms and signs.

Possible (must have **one** of the following)

1. Other potential etiology present (must have **each** of the following):

   a. Same as criteria 1, 2, and 3 in **Probable.**

   b. Other potential etiology is present, but the cause of criterion 1 above is uncertain.

2. Incomplete clinical evaluation (must have **each** of the following):

   a. Same as criteria 1, 2, and 3 in **Probable.**

   b. Etiology cannot be determined (appropriate laboratory or radiologic investigations not performed).

The level of impairment due to cognitive dysfunction should be assessed as follows:

Mild: Decline in performance at work, including work in the home, that is conspicuous to others. Unable to work at usual job, although may be able to work at a much less demanding job. Activities of daily living or social activities are impaired but not to a degree making the person completely dependent on others. More complicated daily tasks or recreational activities cannot be undertaken. Capable of basic self-care such as feeding, dressing, and maintaining personal hygiene, but activities such as handling money, shopping, using public transportation, driving a car, or keeping track of appointments or medications is impaired.

Moderate: Unable to work, including work in the home. Unable to function without some assistance of another in daily living, including dressing, maintaining personal hygiene, eating, shopping, handling money, and walking, but able to communicate basic needs.

Severe: Unable to perform any activities of daily living without assistance. Requires continual supervision. Unable to maintain personal hygiene, nearly or absolutely mute.

Appendix C: KARNOFSKY PERFORMANCE SCALE

100 Normal, no complaints, no evidence of disease
90 Able to carry on normal activity, minor signs or symptoms of disease
80 Normal activity with effort, some signs or symptoms of disease
70 Cares for self, unable to carry on normal activity or to do active work
60 Requires occasional assistance but is able to care for most of his needs
50 Requires considerable assistance and frequent medical care
40 Disabled, requires special care and assistance
30 Severely disabled, hospitalisation is indicated although death is not imminent
20 Very sick, hospitalisation necessary, active supportive treatment is necessary
10 Moribund, fatal processes progressing rapidly
0 Dead

Appendix D: NEUROPSYCHOLOGICAL EXAMS

- **Rey Auditory Verbal Learning Test Trial I-V and Delayed Recall**

  This test measures immediate memory span, provides a learning curve, reveals learning strategies (or their absence), elicits retroactive and proactive interference tendencies and tendencies to confusion or confabulation on memory tasks. It also measures retention following an interpolated activity.

  It consists of two 15 word lists. The first list (A) is read five times by the examiner and the subject is asked to recall as many words as possible after each reading. After completion of trial V, the examiner reads a second list (B) and again asks the subject to recall as many words as possible. Following the B list trial the subject is asked to recall words from list A once more. A 30 minute delayed recall trial is also included which gives additional information on how well the patient can recall what was once learned.

  The examiner writes down the words the patient recalls in the order recalled. The score for each trial is the number of words correctly recalled. The total score is calculated by adding up the scores from each trial.

- **Grooved Pegboard (dominant and non-dominant)**

  This is a manipulative dexterity test that helps to evaluate lateralized brain damage. The Lafayette Model 32025 pegboard will be used. It consists of 25 holes with randomly positioned slots. Pegs which have a key along one side must be rotated to match the hole before they can be inserted. The length of time required to perform each trial to its completion or discontinuation is recorded.

  The task is performed once with the dominant and once with the non-dominant hand. The score will consist of the time (in seconds) taken to complete each trial beginning when the patient starts the task until the last peg is put in. The trial will be discontinued after 240 seconds.

- **Trail Making A and B**

  This test measures visual conceptual and visuomotor tracking abilities. It consists of a worksheet with a set of circled numbers for part A and letters and numbers for part B, randomly placed. The participant is expected to connect the circles consecutively as fast as possible without lifting the pencil. For the part B the two sequences (letters and figures) will need to be alternated e.g. 1-A, 2-B, 3-C, etc.

  Part A is used as a "practice" trial for part B. The total score is the time in seconds it takes the subject to complete part B. Subjects are allowed a

maximum of 240 seconds. If the task is not completed within this time, discontinue the trial and count the number of circles connected correctly.

- **Symbol Digit**

This test provides rapid screening of visual perception and scanning, oculomotor defects or general mental and/or motor slowing.

In this test a key of symbols and corresponding numbers is provided. Subsequently a series of symbols (110) are presented in a random order. The subject is expected to fill in the empty boxes below the symbols with the appropriate numbers, following the key provided. A maximum of 90 seconds per trial is allowed. The score will be the number of boxes correctly completed.

- **Cal-Cap Reaction Time**

This test is modelled after the Continuous Performance Task, a measure of sustained attention and reaction time. It consists of a series of fully normed simple an choice reaction time measures administered and scored by computer. Subjects are asked to focus on a display field and respond only to specific stimuli. An abbreviated version will be used in this study. It comprises those measures from the standard edition that are particularly sensitive to HIV-related cognitive decline, as follows:

Choice Reaction Time for Single Digits. Subjects are asked to press a key as soon as they see a specific number such as "7", otherwise they are to do nothing. This procedure adds a simple element of memory to the task.

Serial Pattern Matching 1 (Sequential Reaction Time 1). Subjects are asked to press a key only when they see two of the same number in sequence, for example, if they see the number "3" followed by a second occurrence of the number "3". This procedure adds a more complex element of memory since the subject must keep in mind the last number that was seen.

In addition to reaction time measures, level of performance on each task is assessed by evaluating the numbers of "Hits" and "False Positives". Signal detection theory provides measures of the subject's ability to discriminate between the true signal and distractor items (d') and of the degree to which the subject deviates from the optimal likelihood reaction (beta). Results are provided as mean and median reaction times, total numbers of true and false positive responses and estimates of the signal detection parameters d' and beta.

- **Verbal Fluency (FAS)**

This test requires the subject to provide as many words as they can, beginning by one of three letters (F, A, S) provided by the examiner,

during the course of one minute. Proper nouns, numbers and variations on the same word are not allowed. The number of correct responses, violations and repetitions are recorded. The overall score corresponds to the number of correct responses.

Appendix E: NEUROLOGICAL EXAMS

The neurological evaluations used by the AIDS Clinical Trials Group (ACTG) will be used in this study.

The following domains will be examined:

1. Cognitive Function
   - a) Concentration/Speed of Thought
   - b) Reading (or TV)
   - c) Memory
   - d) Speech
2. Motor Function
   - a) Gait
   - b) Upper Extrimity Coordination
   - c) Involuntary Movement (Including Tremor, Myoclonus & Chorea)
   - d) Bladder Incontinence
3. Behaviour/Mood
   - a) Mood
   - b) Social Apathy/Withdrawal
   - c) Emotional Liability/Agitation
4. Level of Consciousness
5. Affect
   - a) Degree of Alteration
   - b) Type of Alteration
6. Adapted Mini Mental Status Exam
7. Response Slowing
8. Cranial Nerves
9. Ocular Motility
   - a) Smooth Pursuits

b) Saccades

10. Facial Expression

11. Strength

    a) Right Leg

    b) Left Leg

12. Coordination

    a) Gait Coordination

    b) Limb Coordination

13. Reflexes

    a) Deep Tendon Reflexes

    b) Jaw Jerks

    c) Snout Reflex

14. Neuropathy

    a) Severity of Sensory Loss

    b) Severity of Neuropathy

    c) Type of Neuropathy

Appendix F:
LISTING OF SPECIFIC CLINICAL LABORATORY TESTS

Appendix F1: Listing of Specific Clinical Laboratory Tests: Blood

Haematology
Haemoglobin
MCV
Quantitative Platelet Count
WBC Count
Neutrophils
Lymphocytes
Monocytes
Eosinophils
Basophils

Lymphocyte Subsets
Absolute and % CD4+ and CD8+ counts
Total lymphocytes

Pregnancy Test
Serum β-HCG

Microbiology
VDRL

Virology (In House)
HIV RNA PCR
Genotype
SI/NSI Phenotype

Serum Chemistry
Sodium (Na^+)
Potassium (K^+)
Bicarbonate (HCO_3^-)
Creatinine
Total Bilirubin
Alkaline Phosphatase
ALT
AST
Albumin
Glucose
Amylase
CPK
Cholesterol
Triglycerides

Dipstick Urinalysis
Blood
Protein

Appendix F2: Listing of Specific Clinical Laboratory Tests: Cerebrospinal Fluid

<u>CSF Exclusion Diagnosis Analysis</u>
(To be analysed at site)
Protein
Glucose
Cell Counts
Cell differential
VDRL
Cryptococcal antigen
Fungal culture

<u>CSF Virological Analysis</u>
(Samples to be stored)
HIV RNA PCR
Genotype Characterisation

<u>CSF Markers Analysis</u>
(Samples to be stored)
β_2 microglobulin
Quinolinic acid
TNF receptor 2

Appendix G: VIROLOGY SAMPLES PROCEDURES

1. VIRAL LOAD DETERMINATIONS IN PLASMA (HIV RNA PCR)

Quantitation of viral load in plasma will be carried out by the RNA polymerase chain reaction (PCR) technique developed by Roche Molecular Systems.

IMPORTANT: **Plasma samples must be processed within TWO hour of blood being taken**

1.1 Required Materials

- 2x4 ml lavender stoppered Vacutainer tube containing EDTA dipotassium
- 3x2 ml biofreeze tubes
- Sample labels: 2 for each sample (supplied by Glaxo Wellcome)
- Centrifuge
- Pasteur pipettes/bulbs
- -70°C Freezer
- Shipping materials
- Dry Ice for shipping

1.2 Collection and Processing of Blood Samples

- Collect 6 ml of whole blood into two 4ml lavender topped Vacutainer tubes.
- Immediately invert the tubes GENTLY 8-10 times to mix the EDTA with the blood. DO NOT SHAKE.
- Centrifuge blood samples at 2500 rpm for 10 minutes to separate the plasma from the cells.
- Transfer all plasma from the Vacutainer tubes, dividing the plasma into the 3 biofreeze storage tubes (there should be approximately 1ml in each).
- Affix the pre-printed patient-specific labels to each plasma storage tube BEFORE samples are frozen.
- Freeze the three plasma samples immediately in the upright position in a non-self defrosting freezer at -70°C until shipment to Glaxo Wellcome is arranged.

1.3 Collection and Processing of Cerebro Spinal Fluid Samples

The collection of the CSF during the study is optional for the patient.

1.3.1 *Virological Analysis*

- Optimally, 1 ml sample should be obtained at the same time the pharmacokinetic sample is collected.
- Please transfer the 1 ml CSF sample into two polypropylene tubes labelled with the appropriate pre-printed virology labels.

1.3.2 *CSF Markers Analysis*

- 1.5 ml sample should be obtained at the same time the pharmacokinetic sample is collected.
- Please split the 1.5 ml CSF sample into three polypropylene tubes each containing 0.5 mls.

  NOTE: Please use any volume left over from the pharmacokinetic sample. If less than 0.5 mls of CSF is collected, do not divide the sample, collect it into one tube only.

Freeze the samples upright and store at -70°C.

Analysis	CSF Volume Required	Time Points
Pharmacokinetic	0.5 ml	Weeks 6 and 12
Virology	2 x 0.5ml	Day -7, weeks 6, 12, 20, 36 and 52
CSF Markers		Day -7, weeks 6, 12, 20, 36 and 52
β_2 Microglobulin	0.5 ml	
Quinolinic Acid	0.5 ml	
TNF receptor 2	0.5 ml	
Exclusion Diagnosis	As mandated by local laboratory	Day -7 (Mandatory)

2. VIRAL PHENOTYPE (SI/NSI)

Peripheral blood mononuclear cells will be processed and temporarily stored by the appointed central clinical laboratories until they are shipped to Glaxo Wellcome.

The appointed central laboratories will be providing vacutainer CPT tubes and will be providing instructions of how to collect and process these samples prior to the commencement of the study.

PBMCs will be cultured to determine the SI/NSI phenotype of the virus and subsequently be used as a source of genetic material for additional drug sensitivity assays as deemed appropriate at the time of analysis.

3. SHIPMENT OF SAMPLES

Shipment will be arranged through the appointed central laboratories on an ongoing basis throughout the study. Frequency of shipments will be determined by the number of samples accumulated at site and requirements of analysis of specific samples. The local monitor will act as a central contact for the site and will be able to advise on shipment requirements.

Appendix H: PHARMACOKINETIC SAMPLES PROCEDURES

1. COLLECTION OF BIOLOGICAL SAMPLES FOR PHARMACOKINETIC EVALUATIONS

Population pharmacokinetics will be conducted with CSF and plasma concentration data obtained from this study. The CSF samples will be collected from a subset of patients at weeks 6 and/or 12. CSF samples will be optional.

Patients will be asked to attend the clinic to receive a supervised dose of study drugs on those days when pharmacokinetic samples are to be taken.

1.1 Blood Samples

Required Materials

- 4 ml tube containing powdered EDTA dipotassium
- 3.6 ml fill polypropylene tubes
- Bar-coded labels: 3 for each sample (supplied by Glaxo Wellcome)
- Plastic bags for shipping samples
- Ice or refrigerator
- Dry Ice for shipping
- Pasteur pipettes/bulbs

Collection and processing of Blood Samples

- Collect 3 ml blood samples in labelled tube containing powdered EDTA dipotassium
- Invert the tube gently 8 - 10 times to mix the EDTA with the blood. DO NOT SHAKE. Place on ice or refrigerate upon collection
- Centrifuge blood sample within 30 minutes of collection at 2500 RPM for 10 minutes to separate the plasma from the cells.
- Transfer all plasma from the Vacutainer tube, into a polypropylene tube (approx. 3.5 ml)
- Affix the pre-printed patient-specific bar-code label to each plasma tube. See section 1.3 on use of bar-code labels.
- Freeze plasma samples immediately in the upright position in a non-self-defrosting freezer at - 70°C or lower until they can be shipped to Glaxo Wellcome on dry ice.

1.2 Cerebrospinal Fluid Samples

Collection and processing of CSF samples

The collection of the CSF sample is optional for the patient.

- Each CSF sample should have a matching plasma sample taken at the same time point.
- A 3 ml sample should be obtained at the assigned time following the time schedule described in the protocol section 6.7.2.1.
- Transfer 0.5ml of CSF into a biofreeze tube with the appropriate pharmacokinetic bar-coded pre-printed label. The remaining sample will be used for virology and CSF markers analysis and should be aliquoted as described in Appendix G, section 1.3.2.
- Record the date and actual sample time on the case report form.
- Freeze the sample upright and store at -70°C.

1.3 Instructions for the Use of Bar Code Labels

Bar code labels have been pre-printed in duplicate for both plasma and CSF samples. The first label is used to identify the initial sample as it is drawn from the patient. The second is for the prepared samples in the storage tubes.

The labels are designed to fit on the tubes supplied for the study. **The bar codes can only be read if the labels are correctly attached to the sample tubes.** To ensure the labels are readable, please follow the instructions described below:

- The labels must run lengthways (up to down) on the tubes. Do not wrap the labels around the tubes.
- Please try to prevent any wrinkles forming on the labels.
- Do not put any clear or opaque tape over any part of the labels
- Do not write on or mark the labels. If a sample is not collected at the time that is printed on the label, indicate this change on the case report form and do not attempt to write it on the label.

Incorrectly labelled samples will **NOT** be analysed by the clinical assay laboratory.

2. SHIPMENT OF SAMPLES

Shipment will be arranged through the appointed central laboratories on an ongoing basis throughout the study. Frequency of shipments will be

determined by the number of samples accumulated at site and requirements of analysis of specific samples. The local monitor will act as a central contact for the site and will be able to advise on shipment requirements.

Appendix I: ADVERSE EXPERIENCE DATA HANDLING GUIDELINES

- **Adverse experience definition:** "Any undesirable medical experience occurring to a subject, during a clinical trial, whether or not related to the study drug(s)." An event may consist of a disease, an exacerbation of a pre-existing illness, a recurrence of an intermittent illness, a set of related symptoms or signs or a single symptom or sign.

- **Lack of efficacy:** Lack of efficacy of a study medication should not be recorded as an adverse experience as the expected efficacy has not been established.

- **Diagnosis versus individual symptoms:** When possible, the medically accepted diagnosis should be reported as the adverse experience, rather than the individual symptoms.

- **Change in intensity:** Record each adverse experience once with the maximum intensity/seriousness that occurred over the duration of the adverse experience.

- **Intermittent adverse experiences:** Intermittent adverse experiences should be recorded once at its maximum intensity/seriousness to cover the total duration and the word "intermittent" written in the comment field.

- **Adverse experience(s) precipitating death:** Record the adverse experience(s) precipitating death on the adverse experience page. Do not record any secondary or tertiary adverse experiences which are involved in the death process. These will be collected on the supporting documents including the autopsy report, the SAE form, etc.

- **Abnormal lab values or other findings:** With the exception of grade 4, abnormal laboratory values or other findings without an associated clinical diagnosis should not be recorded on the adverse experience page as these values will be collected elsewhere within the CRF.

- **Adverse experience data recorded in other areas of the CRF:**

    - All serious adverse experiences (SAE) should also be recorded on the adverse experience page.

- **Adverse experiences while receiving combination therapy:** Causality of adverse experiences which occur while receiving combination therapy should be assigned to the combination and not an individual agent.

- **Pre-existing conditions:** Pre-existing conditions are to be recorded as adverse experiences only if they change, worsen in intensity/seriousness, or if they were intermittent prior to initiation of study treatment and they recurred after treatment was initiated.

- **Ongoing adverse experiences:** Adverse experiences which are present when a subject completes the protocol-specified treatments/evaluations or when a subject dies, should be recorded as "ongoing" at the end of the study.

- **Post-treatment adverse experience collection:** Record all adverse experiences within the initial 7 to 10 days post-treatment period and only attributable SAEs thereafter.

- **Hospitalization/elective procedures:** An elective procedure is not an adverse experience and a hospitalization alone is not an SAE.

- **Multiple/ambiguous terms:** Record only one adverse experience per space. Please do not use ambiguous terms when reporting an adverse experience. Only use terms which are interpretable on their own merit or clarify the term in the comment field if a more suitable term is not available.

Appendix J: TABLES FOR GRADING THE INTENSITY OF ADVERSE EXPERIENCES

Appendix J1: Laboratory Test Abnormalities

Table for Grading Severity of Adverse Experiences for Dose Modification Purposes and Data Analysis

ITEM	GRADE 1 TOXICITY	GRADE 2 TOXICITY	GRADE 3 TOXICITY	GRADE 4 TOXICITY
**HEMATOLOGY**				
Hemoglobin	8.0-9.4 gm/dL	7.0-7.9 gm/dL	6.5-6.9 gm/dL	<6.5 gm/dL
Absolute Neutrophil Count	1000-1500/mm^3	750-999/mm^3	500-749/mm^3	<500/mm^3
Absolute WBC Count	2001-3000/mm^3	1501-2000/mm^3	1001-1500/mm^3	<1000/mm^3
Platelets	75,000-99,000/mm^3	50,000-74.999/mm^3	20,000-49,999/mm^3	<20,000/mm^3 or diffuse petechiae
Prothrombin Time (PT)	1.0-1.25 X upper normal limit	>1.25-1.5 X upper normal limit	>1.5-3.0 X upper normal limit	>3 X upper normal limit
Activated Partial Thromboplastin Time (APTT)	>1.0-1.66 X upper normal limit	>1.66-2.33 X upper normal limit	>2.33-3 X upper normal limit	>3 X upper normal limit
Fibrinogen	0.75-0.99 X lower normal limit	0.50-0.74 X lower normal limit	0.25-0.49 X lower normal limit	<0.25 X lower normal limit
Fibrin Split Product	20-40 µg/ml	41-50 µg/ml	51-60 µg/ml	>60 µg/ml
Methemoglobin	5-10.0%	10.1-15%	15.1-20%	>20%
**ENZYMES**				
AST (SGOT)	1.25-2.5 X upper normal limit	>2.5-5 X upper normal limit	>5.0-10 X upper normal limit	>10 X upper normal limit
ALT (SGPT)	1.25-2.5 X upper normal limit	>2.5-5 X upper normal limit	>5.0-10 X upper normal limit	>10 X upper normal limit
GGT	1.25-2.5 X upper normal limit	>2.5-5 X upper normal limit	>5.0-10 X upper normal limit	>10 X upper normal limit
Alkaline Phosphatase	1.25-2.5 X upper normal limit	>2.5-5 X upper normal limit	>5.0-10 X upper normal limit	>10 X upper normal limit
Amylase	1.1-1.5 X upper normal limit	>1.5-2.0 X upper normal limit	>2.0-5.0 X upper normal limit	>5.1 X upper normal limit
Creatine phosphokinase (CPK)	>1.0-2.0 X upper normal limit	>2.0-4.0 X upper normal limit	>4.0-6.0 X upper normal limit	>6.1 X upper normal limit
**CHEMISTRIES**				
Hyponatremia	130-135 meq/L	123-129 meq/L	116-122 meq/L	<116 meq/L OR mental status changes OR seizures
Hypernatremia	146-150 meq/L	151-157 meq/L	158-165 meq/L	>165 meq/L OR mental status changes OR seizures
Hypokalemia	3.0-3.4 meq/L	2.5-2.9 meq/L OR replacement Rx req	2.0-2.4 meq/L OR intensive replacement Rx req. OR hospitalization req.	<2.0 meq/L OR paresis OR ileus OR life-threatening arrhythmias
Hyperkalemia	5.6-6.0 meq/L	6.1-6.5 meq/L	6.6-7.0 meq/L	>7.0 meq/L OR life-threatening arrhythmias

NO TAG (Continued)

ITEM	GRADE 1 TOXICITY	GRADE 2 TOXICITY	GRADE 3 TOXICITY	GRADE 4 TOXICITY
**CHEMISTRIES (Cont.)**				
Hypoglycemia	55-64 mg/dL	40-54 mg/dL	30-39 mg/dL	<30 mg/dL OR mental status changes OR coma
Hyperglycemia: (nonfasting and no prior diabetes)	116-160 mg/dL	161-250 mg/dL	251-500 mg/dL	>500 mg/dL OR ketoacidosis OR seizures
Hypocalcemia - correct for albumin	7.8-8.4 mg/dL	7.0-7.7 mg/dL	6.1-6.9 mg/dL	<6.1 mg/dL OR life-threatening arrhythmias OR tetany
Hypercalcemia - correct for albumin	10.6-11.5 mg/dL	11.6-12.5 mg/dL	12.6-13.5 mg/dL	>13.5 mg/dL OR life-threatening arrhythmia
Hypomagnesemia	1.2-1.4 meq/dL	0.9-1.1 meq/L OR replacement Rx req.	0.6-0.8 meq/L OR intensive Rx req. OR hospitalization	<0.6 meq/L OR life-threatening arrhythmia
Hypophosphatemia	2.0-2.4 mg/dL	1.5-1.9 mg/dL OR replacement Rx req.	1.0-1.4 mg/dL OR intensive Rx req. OR hospitalization req.	<1.0 mg/dL OR life-threatening arrhythmias
Hyperbilirubinemia	>1.0-1.5 X upper normal limit	>1.5-2.5 X upper normal limit	>2.5-5 X upper normal limit	>5 X upper normal limit
Blood Urea Nitrogen (BUN)	>1.25-2.5 X upper normal limit	>2.5-5 X upper normal limit	>5.0-10 X upper normal limit	>10 X upper normal limit
Creatinine	>1.0-1.5 X upper normal limit	>1.5-3.0 X upper normal limit	>3.0-6 X upper normal limit	>6 X upper normal limit OR dialysis required
Serum Lactate	>1.0-2.0 X upper normal limit	>2.0-3.0 X upper normal limit	>3.0-3.9 X upper normal limit	>4.0 X upper normal limit
Albumin	>1.0-1.5X upper normal limit	>1.5-2.0 X upper normal limit	>2.0-5.0 X upper normal limit	>5.0 X upper normal limit
Triglycerides	ULN-399 mg/dL or ULN-4.50 mmol/L	400-750 mg/dL or 4.51-8.50 mmol/L	751-1200 mg/dL or 8.51-13.50 mmol/L	>1200 mg/dL or >13.50 mmol/L
Cholesterol	>1.0-1.3 X upper normal limit	>1.3-1.6 X upper normal limit	>1.6-2.0 X upper normal limit	>2.0 X upper normal limit
**URINALYSIS**				
Proteinuria	1+ OR <0.3% OR <3 g/L OR 200 mg-1 gm loss/day	2-3+ OR 0.3-1.0% OR 3-10 g/L OR 1-2 gm loss/day	4+ OR >1.0% OR >10 g/L OR 2-3.5 gm loss/day	>3.5 gm loss/day OR nephrotic syndrome
Hematuria	microscopic only	gross, no clots	gross+clots	requires transfusion OR obstructive

Appendix J2: Clinical Adverse Experiences

Table for Grading Severity of Adverse Experiences for Dose Modification Purposes

ITEM	GRADE 1 TOXICITY	GRADE 2 TOXICITY	GRADE 3 TOXICITY	GRADE 4 TOXICITY
**CARDIAC DYSFUNCTION**				
Cardiac Arrhythmia	- -	asymptomatic, transient dysrhythmia, no Rx required	recurrent/persistent; dysrhythmia; symptomatic Rx required	unstable dysrhythmia, hospitalization and Rx required
Hypertension	transient inc. >20 mm/Hg; no Rx	recurrent, chronic, >20 mm/Hg, Rx req.	requires acute therapy; outpatient hospitalization possible	requires hospitalization
Hypotension	transient orthostatic hypotension; No Rx	symptoms correctable with oral fluid Rx	requires IV fluids no. hosp. required	requires hospitalization
Pericarditis	minimal effusion	mild/mod asymp. effusion, no Rx	symptomatic effusion, pain, EKG changes	tamponade; OR pericardiocentesis OR surgery required
Hemorrhage, Blood Loss	- -	mildly symptomatic or Rx required	gross blood loss; OR 1-2 units transfused	massive blood loss OR >2 units transfused
**RESPIRATORY**				
Cough - for aerosol studies	transient - no Rx	treatment associated cough; inhaled bronchodilator	uncontrolled cough systemic Rx required	- -
Bronchospasm acute	transient; no Rx; <80%->70% FEV_1 (or peak flow)	req. Rx; normalizes with bronchodilator FEV_1 50%-70% (or peak flow)	no normalization w/ bronchodilator FEV_1 25%-50% (or peak flow), retractions	cyanosis FEV_1 <25% (or peak flow) OR intubated
**NEUROLOGIC**				
Neuro-Cerebellar	slight incoordination OR dysdiadokinesia	intention tremor, OR dysmetria, or slurred speech; OR nystagmus	Ataxia requiring assistance to walk or arm incoordination interfering with ADLS	unable to stand
Neuro-psych/mood	- -	- -	severe mood changes requiring medical intervention	acute psychosis require hospitalization

NO TAG (Continued)

ITEM	GRADE 1 TOXICITY	GRADE 2 TOXICITY	GRADE 3 TOXICITY	GRADE 4 TOXICITY
**NEUROLOGIC (continued)**				
Paresthesia (burning, tingling, etc)	mild discomfort; no Rx req	mod discomfort; non-narcotic analgesia req	severe discomfort; OR narcotic analgesia req with symptomatic improvement	incapacitating; OR not responsive to narcotic analgesia
Neuro-motor	mild weakness in muscle of feet but able to walk and/or mild increase or decrease in reflexes	Mod weakness in feet (unable to walk on heels and/or toes), mild weakness in hands, still able to do most hand tasks and/or loss of previously present reflex or development of hyperreflexia and/or unable to do deep knee bends due to weakness	Marked distal weakness (unable to dorsiflex toes or foot drop), and mod proximal weakness e.g., in hands interfering with ADLs and/or requiring assistance to walk and/or unable to rise from chair unassisted	confined to bed or wheel chair because of muscle weakness
Neuro-sensory	Mild impairment (dec sensation, e.g., vibratory, pinprick, hot/cold in great toes) in focal area or symmetrical distribution	Mod impairment (mod dec sensation, e.g., vibratory, pinprick, hot/cold to ankles) and/or joint position or mild impairment that is not symmetrical	severe impairment (dec or loss of sensation to knees or wrists) or loss of sensation of at least mod degree in multiple different body areas (i.e., upper and lower extremities)	sensory loss involves limbs and trunk.
**GASTROINTESTINAL**				
Nausea	mild discomfort; maintains reasonable intake	mod. discomfort; intake dec. for < 3 days	severe discomfort; minimal intake for ≥3 days	hospitalization required
Constipation	mild	moderate	severe	distention with vomiting
Abdominal pain	mild discomfort; no limits on activity	mild-moderate discomfort; no Rx required	moderate pain; Rx required	severe pain; hospitalization required
Vomiting	mild or transient; 2-3 episodes per day OR mild vomiting lasting < 1 week	mod or persistent; 4-5 episodes per day OR vomiting lasting ≥ 1 week	severe vomiting of all food/fluids in 24 hrs OR orthostatic hypotension OR IV Rx req.	hypotensive shock OR hospitalization req. for IV Rx req.
Diarrhea	mild or transient; 3-4 loose stools per day OR mild diarrhea lasting < 1 week	mod OR persistent; 5-7 loose stools per day OR diarrhea lasting ≥ 1 week	bloody diarrhea, OR orthostatic hypotension OR > 7 loose stools/day OR IV Rx req.	hypotensive shock OR hospitalization req.
Oral dyscomfort/ dysphagia	mild discomfort, no difficulty swallowing	difficulty swallowing but able to eat and drink	unable to swallow solids	unable to drink fluids; IV fluids req.

NO TAG (Continued)

ITEM	GRADE 1 TOXICITY	GRADE 2 TOXICITY	GRADE 3 TOXICITY	GRADE 4 TOXICITY
**OTHER PARAMETERS**				
Fever oral, >12 hours	37.7-38.5C OR 100.0-101.5F	38.6-39.5C OR 101.6-102 F	39.6-40.5C OR 103-105F	>40.5C OR >105F
Headache	mild, no Rx therapy	mod; OR non-narcotic analgesia Rx	severe OR responds to initial narcotic therapy	intractable, OR req. repeated narcotic therapy
Fatigue	normal activity reduced <25%	normal activity dec. 25-50%	normal activity dec. >50%; can't work	unable to care for self
Allergic Reaction	pruritus w/o rash	localized urticaria	generalized urticaria angioedema	anaphylaxis
Cutaneous/Rash/ Dermatitis	erythema, pruritus	diffuse, maculopapular rash, OR dry desquamation	vesiculation, OR moist desquamation, OR ulceration	exfoliative dermatitis, OR mucous membrane involvement, OR erythema, multiforme OR suspected Stevens-Johnson OR necrosis requiring surgery
Local Reaction (2° parenteral Rx not vaccination or skin test.	Erythema	induration <10 mm OR inflammation OR phlebitis	induration >10 mm OR ulceration	necrosis of skin

Appendix K: OPERATING PRINCIPLES OF END-POINT COMMITTEE

Composition :

A/Prof. Brew	(St Vincent's Hospital, Sydney, Australia)
Dr. Catalan	(Chelsea and Westminster Hospital, London, UK)
A/Prof. McArthur	(Johns Hopkins University, Baltimore, US)
Dr. Portegies	(Academic Medical Centre, Amsterdam, Holland)

Objectives :

1. To review and endorse all clinical dementia related end-points from study CNAA3001
2. To endorse any premature commencement of the study's open phase.

Purpose :

To standardise clinical dementia related end-points across participating centres

Operating principles:

End-points review will be coordinated by a designated scientist from Glaxo Wellcome.

Reviews will be carried out by faxed documentation which would include Neurological Evaluations, ADC rating, and Neuropsychological Evaluations Summary CRF pages plus supporting documentation

Expected turn around time would be 48 hours in order not to delay inclusion of patient on the open label phase.

Patients should not be enrolled into the open label phase until the end-point is confirmed.

Three votes per end-point would be required.

The origin of the end-point would be blinded.

If the end-point is generated by one of the investigators of the end-point committee he would not be asked to comment. The identity of the participating investigators of any given end-point review will not be revealed.

Appendix L: PATIENT AND CAREGIVER QUESTIONNAIRES

Appendix L1: Patient Health Status Questionnaires

To document the extent that addition of 1592U89 to current therapy improves patients physical and psychological functioning, patients will be asked to self-administer questionnaires containing the Medical Outcomes Study Health Status Questionnaire for HIV (MOS-HIV; 32) during clinic visits at weeks 0, 6, 12 and study discontinuation.

The MOS-HIV, a multidimensional self-report questionnaire, is the most extensively validated instrument for tracking HIV-infected patient's self-assessed health status. Its thirty questions allow patients to report their perceived health-related quality of life and functional status on eleven domains. MOS-HIV domains correspond to two dimensions of health status -- Physical and Psychological Health Status. The physical health status dimension consists of four scales assessing physical functioning (PF), role limitations - the ability to perform normal activities (RL), bodily pain (BP), and vitality (EF) domains. Domains associated with psychological health status include cognitive functioning (CF), mental health (MH), health distress (HD), and social functioning (SF). Two additional domains summarize perceived health (GHP) and global quality of life (QOL). The final domain captures how patients perceive their health status today compared with their health status 4 weeks ago using a single-item health transition (HT) scale. Scales of the MOS-HIV (except BP, SF, QOL, and HT) are based on the sum of two or more items and have a range of 0 (worst) to 100 (best).

Validity and reliability of the MOS-HIV have been examined in a number of studies including ACTG 019, 114, 116/117, and 118 (NO TAG), as well as in the North American pivotal trials for Epivir/Retrovir (NUCA3001, NUCA3002) (NO TAG-NO TAG). Multiple-item scales of the MOS-HIV consistently demonstrate high internal consistency (NO TAG). Its physical dimension scales tend to be more sensitive to changes over time than are psychological dimension scales, although psychological dimension scales have been shown to discriminate between patients with and without diagnosed major depression, or anxiety (NO TAG). Studies have also demonstrated that the MOS-HIV can detect clinically relevant differences, such as differences between HIV disease stages. Translations from the original US English to UK English (NO TAG,NO TAG) and Dutch (NO TAG) have demonstrated content and construct validity.

The US English version of the Patient Health Status Questionnaire, including the MOS-HIV, is attached.

HEALTH STATUS QUESTIONNAIRE (MOS-HIV version 2.1)

Instructions to the Coordinator:

Please complete the header information and items A & B on this page, then read the instructions to the patient. Hand the patient the questionnaire. If s/he does not wish to complete it, please have him/her indicate that s/he was given the opportunity to do so by writing her/his initials in the spaces provided in the box at the bottom of the page.

A. Today's Date ☐☐ Month ☐☐ Day ☐☐ Year

B. Study Visit Week this questionnaire was completed:

☐	☐	☐	☐	☐	☐	☐	☐	☐
Day 0	Week 6	Week 12	Week 20	Week 28	Week 36	Week 44	Week 52	Follow up

To be read to the Patient:

"This survey asks for your views about your health. This information will be summarized and will help keep track of how you feel and how well you are able to do your usual activities.

To complete the survey, please read the instructions for how to indicate your responses. Some questions ask you to check a box, others ask you to circle the answer you choose, and some questions request that you fill in information. Please mark your responses clearly. If you are unsure about how to answer any question, please give the best answer you can. It is important for you to answer EVERY question."

Please ask the patient to initial in the space below if he or she refuses to complete the questionnaire.

I was given the opportunity to complete this questionnaire.	
__________________<br>First Middle Last	__________________<br>Date

American English Version 2.1

Medical Outcomes Study
HEALTH STATUS QUESTIONNAIRE

(MOS-HIV© Version 2.1)

1. In general, would you say your health is:

Circle One

Excellent	1
Very Good	2
Good	3
Fair	4
Poor	5

2. How much <u>bodily</u> pain have you had during the <u>past 4 weeks</u>?

Circle One

None	1
Very Mild	2
Mild	3
Moderate	4
Severe	5
Very Severe	6

© Glaxo Wellcome PLC, 1996

	3. For how long (if at all) has your health limited you in each of the following activities?	Limited for more than 3 months	Limited in the last 3 months	Not limited at all
a.	The kinds or amounts of vigorous activities you can do, like lifting heavy objects, running or participating in strenuous sports.	1	2	3
b	The kinds or amounts of moderate activities you can do, like moving a table, carrying groceries or bowling.	1	2	3
c.	Walking uphill or climbing a few flights of stairs.	1	2	3
d.	Bending, lifting, or stooping.	1	2	3
e.	Walking one hundred yards.	1	2	3
f.	Eating, dressing, bathing, or using the toilet.	1	2	3

		YES, for more than 3 months	YES, for 3 months or less	NO
4.	Does your health keep you from working at a job, doing work around the house, or going to school?	1	2	3
5.	Have you been unable to do certain kinds or amounts of work, housework, or schoolwork because of your health?	1	2	3

	How much of the time, during the past month...	All of the time	Most of the time	A good bit of the time	Some of the time	A little of the time	None of the time
6.	has your health limited your social activities (like visiting with friends or close relatives)?	1	2	3	4	5	6
7.	have you been a very nervous person?	1	2	3	4	5	6
8	have you felt calm and peaceful?	1	2	3	4	5	6
9.	have you felt downhearted and blue?	1	2	3	4	5	6
10.	have you been a happy person?	1	2	3	4	5	6
11.	have you felt so down in the dumps that nothing could cheer you up?	1	2	3	4	5	6
12. a	How often during the last four weeks did you feel full of pep?	1	2	3	4	5	6
b.	did you feel worn out?	1	2	3	4	5	6
c.	did you feel tired?	1	2	3	4	5	6
d.	did you have enough energy to do the things you wanted to do?	1	2	3	4	5	6
e.	did you feel weighed down by your health problems?	1	2	3	4	5	6
f.	were you discouraged by your health problems?	1	2	3	4	5	6
g.	did you feel despair over your health problems?	1	2	3	4	5	6
h.	were you afraid because of your health?	1	2	3	4	5	6

	How much of the time during the past month did you...	All of the time	Most of the time	A good bit of the time	Some of the time	A little of the time	None of the time
13.	have difficulty reasoning and solving problems, for example, making plans, making decisions, learning new things?	1	2	3	4	5	6
14.	forget, for example, things that happened recently, where you put things, appointments?	1	2	3	4	5	6
15.	have trouble keeping your attention on any activity for long?	1	2	3	4	5	6
16.	have difficulty doing activities involving concentration and thinking?	1	2	3	4	5	6

17. How has the quality of your life been during the past 4 weeks?
i.e. How have things been going for you?

Circle One

Very well; could hardly be better 1

Pretty good 2

Good and bad parts about equal 3

Pretty bad 4

Very bad; could hardly be worse 5

18. How would you rate your physical health and emotional condition now compared to 4 weeks ago?

Circle One

Much better 1

A little better 2

About the same 3

A little worse 4

Much worse 5

with permission, Albert W. Wu, M.D., M.P.H.
The Johns Hopkins University

Appendix L2: HIV Caregiver Impact Assessment (HIV-CIA)

GlaxoWellcome

Project number: **131**
GOLD protocol
number: **CNAB3001**

HIV Caregiver Impact Assessment©

(HIV-CIA version 2.0)[1]

BASELINE

Instructions:

Please sign your initials in this box

to show you were given this
questionnaire to complete

This booklet contains a series of questions we would like you to answer that describe how you and the person you help who has HIV infection are doing. Your answers will be kept confidential but will provide important information on how well the person you help is able to care for himself or herself.

When you finish answering these questions, please return this booklet to the person who gave it to you.

Thank you for your time and help.

Study Coordinator: Please store this booklet in the patient's QOL binder

[1] The HIV-CIA v 2.0 was developed by Research Triangle Institute for Glaxo Wellcome Inc.
© 1996 by Glaxo Wellcome Inc.

GlaxoWellcome

Project number: 131
GOLD protocol
number: CNAB3001

HIV Caregiver Impact Assessment©
BASELINE

When it comes to helping someone with HIV infection, you are the expert. That is why we would like you to answer a series of questions in this booklet. These questions were created to find out how well people with HIV infection are able to do everyday things. They also will help doctors and researchers understand what helping someone with HIV infection does to the health and everyday lives of HIV caregivers (people like you who care for someone who is living with HIV infection).

Your answers are important. Please read the instructions below, then take a few minutes to complete the questionnaire.

Instructions:

It takes only a few minutes to answer all of the questions in this booklet. It is important that you answer EVERY question. You are the expert. If you are not sure how to answer a question, give the best answer you can.

Most questions in this booklet ask you to place a "✓" in the box below or beside the answer that you think best describes you or the person you help. The example below shows the correct way to mark your answer if you are a female.

Example:

What is your sex?
- ☐ Male
- ☑ Female

Please mark your answers in ink in this booklet. If you want to change your answer to a question you have already marked, please draw a single line through the incorrent answer, then mark the correct answer.

When you finish answering all the questions, please give this booklet to the person who gave it to you.

GlaxoWellcome
CONFIDENTIAL

Page600

Project Number: 131	
GOLD protocol number: CNAB3001	

HIV Caregiver Impact Assessment© (v.2.0)
BASELINE

Site number:	Subject's Initials:	Subject's number:	Session number: 3

(Please ✓ one answer per question)

1. What is your birth date? ______ Month ______ Day ______ Year
2. What is your sex? ☐ Male ☐ Female
3. Which of these describes your current employment status? (please ✓ one)
   - ☐ Employed full-time
   - ☐ Employed part-time
   - ☐ Unemployed/looking for job
   - ☐ Disabled
   - ☐ Retired, homemaker, student
4. How long have you known the person you help?
   - ☐ Less than one year
   - ☐ ________ year(s)
5. Do you live with the person you help? ☐ Yes ☐ No
6. Which of these best describes how you are related to the person you help (please check only one)

   The person I help is my
   - ☐ friend
   - ☐ partner or spouse
   - ☐ parent
   - ☐ child
   - ☐ brother or sister
   - ☐ other relative
   - ☐ neighbor or church member
   - ☐ employer or patient (I am a professional caregiver)
7. How long have you been helping this person?
   - ☐ Less than one year
   - ☐ ________ year(s)
8. On average, how much time did you spend helping this person during the past 30 days?
   - ☐ Less than 1 hour per week
   - ☐ Several hours per week
   - ☐ 1 or 2 hours per day
   - ☐ 3 to 6 hours per day
   - ☐ 7 to 12 hours per day
   - ☐ More than 12 hours per day

GlaxoWellcome
CONFIDENTIAL

Project Number: 131	**HIV Caregiver Impact Assessment© (v.2.0)**
	**BASELINE**
GOLD protocol number: CNAB3001	

Site number:	Subject's Initials:	Subject's number:	Session number: 3

Thinking about the person you help, how much assistance did he or she need to do the following things during the past 30 days?

(Put one ✓ per line in the box that best describes the person you help)

	How much help did he or she need during the past 30 days?						
	Sometimes did without help				*Always needed help*		
	**Did easily without help**	**Hard to do but did not need help**	**Rarely needed help**	**Often needed help**	**Always needed *a little help***	**Always needed *a lot of help***	**Could not do at all**
	6	5	4	3	2	1	0
Example Climb 12 steps			✓				
9. Bathe or shower							
10. Fill out forms							
11. Shop for groceries							
12. Fix a meal or snack							
13. Remember things, like appointments or to take medicines							
14. Get dressed							
15. Manage finances or pay bills							

GlaxoWellcome
CONFIDENTIAL

Project Number: 131
GOLD protocol number: CNAB3001

HIV Caregiver Impact Assessment© (v.2.0)
BASELINE

Site number:	Subject's initials:	Subject's number:	Session number: 3

Thinking about the person you help, how much assistance did he or she need to do the following things during the past 30 days?

(Put one ✓ per line in the box that best describes the person you help)

	How much help did he or she need during the past 30 days?						
	Sometimes did without help				*Always needed help*		
	Did easily without help	Hard to do but did not need help	Rarely needed help	Often needed help	Always needed *a little help*	Always needed *a lot of help*	Could not do at all
	6	5	4	3	2	1	0
16. Brush teeth or hair							
17. Wash dishes or clothes							
18. Make a telephone call							
19. Feed himself or herself							
20. Follow instructions from the doctor							
21. Count money							
22. Walk from room to room							

GlaxoWellcome
CONFIDENTIAL

Project Number: 131	
GOLD protocol number: CNAB3001	

HIV Caregiver Impact Assessment© (v.2.0)
BASELINE

Site number:	Subject's initials:	Subject's number:	Session number: 3

Thinking about the person you help, how much assistance did he or she need to do the following things during the past 30 days?

(Put one ✓ per line in the box that best describes the person you help)

	How much help did he or she need during the past 30 days?						
	Sometimes did without help				*Always needed help*		
	**Did easily without help**	**Hard to do but did not need help**	**Rarely needed help**	**Often needed help**	**Always** needed ***a little help***	**Always** needed ***a lot of help***	**Could not do at all**
	6	5	4	3	2	1	0
23. Take medicine, change bandages or connect IVs							
24. Get on or off the toilet							
25. Remember the time, date and day of week							
26. Get in or out of bed							

(Please ✓ only one)

27. Does the person you help need **more** help, **less** help or **about the same amount** of help today as he or she needed 30 days ago?

- ☐ needs more help now
- ☐ needs about the same amount of help now
- ☐ needs less help now

GlaxoWellcome
CONFIDENTIAL

Project Number: 131			
[redacted]			
GOLD protocol number: CNAB3001			
Site number:	Subject's initials:	Subject's number:	Session number: 3

HIV Caregiver Impact Assessment© (v.2.0)

BASELINE

During the past 30 days, how would you describe how you have been doing?

(Please ✓ one box on each line)

During the past 30 days....	Could not be better	Very Good	Good	Fair	Poor	Very Poor	Could not be worse
	6	5	4	3	2	1	0
28. ...how have your spirits been?							
29. ...how has your physical health been?							
30. ...how would you describe your ability to do your job, things around the house, or chores?							
31. ...how has your sleep been?							
32. ...how has your social life been?							
33. ...how would you describe your energy level most of the time?							
34. ...how would you describe your financial situation?							
35. ...how well have you been getting along with the people close to you?							

GlaxoWellcome
CONFIDENTIAL

Project Number: 131	**HIV Caregiver Impact Assessment© (v.2.0)** **BASELINE**		
GOLD protocol number: CNAB3001			
Site number:	Subject's initials:	Subject's number:	Session number: 3

(Please ✓ only one)

36. In general, how would you describe the quality of your life today?

- ☐ Excellent
- ☐ Very good
- ☐ Good
- ☐ Fair
- ☐ Poor
- ☐ Very poor

Thank you for your time and your answers.

Please give this booklet back to the person who gave it to you.

Appendix L3: Other Pharmacoeconomic Evaluations

If a patient has un unscheduled physician visit or hospitalization, information on diagnostic procedures and additional treatment associated with this incident may differentiate patient outcomes of treatment. To assist in this comparison, an Unscheduled Visit/Hospitalization Procedure Form has been incorporated in this study. ( ).

Appendix M: ABPI COMPENSATION

CLINICAL TRIALS - COMPENSATION FOR PATIENTS

Glaxo Research and Development Limited

GUIDELINES

Introduction

The following undertaking applies to all Phase II and Phase III clinical studies sponsored by Glaxo Research and Development Ltd unless the study is carried out in a country where there is a national scheme which provides compensation of an equivalent nature.

In the case of trials on marketed products where a product licence exists authorising supply for administration under the conditions of the trial, the GRD Compensation Guidelines will only apply to injuries arising as a result of a placebo or procedures undertaken in accordance with the protocol to which a patient would not be exposed during normal treatment. With respect to other injuries the normal rules regarding compensation will apply.

Glaxo Research and Development Ltd undertakes to adhere to the Clinical Trial Compensation Guidelines of the Association of the British Pharmaceutical Industry of January 1991, except that it undertakes that it will not impose the limitation in Section 3.3 which would permit the exclusion of compensation in respect of bodily injury resulting from the failure of a placebo to provide a therapeutic benefit.

ABPI CLINICAL TRIAL COMPENSATION GUIDELINES.

Preamble

The Association of the British Pharmaceutical Industry favours a simple and expeditious procedure in relation to the provision of compensation for injury caused by participation in clinical trials. The Association therefore recommends that a member company sponsoring a clinical trial should provide without legal commitment a written assurance to the investigator - and through him to the relevant research ethics committee -that the following Guidelines will be adhered to in the event of injury caused to a patient attributable to participation in the trial in question.

1. Basic Principles

1.1 Notwithstanding the absence of legal commitment, the company should pay compensation to patient-volunteers suffering bodily injury (including death) in accordance with these Guidelines.

1.2 Compensation should be paid when, on the balance of probabilities, the injury was attributable to the administration of a medicinal product under trial or any clinical intervention or procedure provided for by the protocol that would not have occurred but for the inclusion of the patient in the trial.

1.3 Compensation should be paid to a child injured in utero through the participation of the subject's mother in a clinical trial as if the child were a patient-volunteer with the full benefit of these Guidelines.

1.4 Compensation should only be paid for the more serious injury of an enduring and disabling character (including exacerbation of an existing condition) and not for temporary pain or discomfort or less serious or curable complaints.

1.5 Where there is an adverse reaction to a medicinal product under trial and injury is caused by a procedure adopted to deal with that adverse reaction, compensation should be paid for such injury as if it were caused directly by the medicinal product under trial.

1.6 Neither the fact that the adverse reaction causing the injury was foreseeable or predictable, nor the fact that the patient has freely consented (whether in writing or otherwise) to participate in the trial should exclude a patient from consideration for compensation under these Guidelines, although compensation may be abated or excluded in the light of the factors described in paragraph 4.2 overleaf.

1.7 For the avoidance of doubt, compensation should be paid regardless of whether the patient is able to prove that the company has been negligent in relation to research or development of the medicinal product under trial or that the product is defective and therefore, as the producer, the company is subject to strict liability in respect of injuries caused by it.

2. Type of Clinical Research Covered

2.1 These Guidelines apply to injury caused to patients involved in Phase II and Phase III trials, that is to say, patients under treatment and surveillance (usually in hospital) and suffering from the ailment which the medicinal product under trial is intended to treat but for which a product licence does not exist or does not authorise supply for administration under the conditions of the trial.

2.2 These Guidelines do not apply to injuries arising from studies in non-patient volunteers (Phase I), whether or not they are in hospital, for which separate Guidelines for compensation already exist.

2.3 These Guidelines do not apply to injury arising from clinical trials on marketed products (Phase IV) where a product licence exists authorising supply for administration under the conditions of the trial, except to the extent that the injury is caused to a patient as a direct result of procedures undertaken in accordance with the protocol (but not any product administered) to which the patient would not have been exposed had treatment been other than in the course of the trial.

2.4 These Guidelines do not apply to clinical trials which have not been initiated or directly sponsored by the company providing the product for research. Where trials of products are initiated independently by doctors under the appropriate Medicines Act 1968 exemptions, responsibility for the health and welfare of patients rests with the doctors alone (see also paragraph 5.2 overleaf).

3. Limitations

3.1 No compensation should be paid for the failure of a medicinal product to have its intended effect or to provide any other benefit to the patient.

3.2 No compensation should be paid for injury caused by other licensed medicinal products administered to the patient for the purpose of comparison with the product under trial.

3.3 No compensation should be paid to patients receiving placebo in consideration of its failure to provide a therapeutic benefit.*

3.4 No compensation should be paid (or it should be abated as the case may be) to the extent that the injury has arisen:

3.4.1 through a significant departure from the agreed protocol;

3.4.2 through the wrongful act or default of a third party, including a doctor's failure to deal adequately with an adverse reaction;

3.4.3 through contributory negligence by the patient.

4. Assessment of Compensation

4.1 The amount of compensation paid should be appropriate to the nature, severity and persistence of the injury and should in general terms be consistent with the quantum of damages commonly awarded for similar injuries by an English Court in cases where legal liability is admitted.

4.2 Compensation may be abated, or in certain circumstances excluded, in the light of the following factors (on which will depend the level of risk the patient can reasonably be expected to accept);

4.2.1 the seriousness of the disease being treated, the degree of probability that adverse reactions will occur and any warnings given;

4.2.2 the risks and benefits of established treatments relative to those known or suspected of the trial medicine.

This reflects the fact that flexibility is required given the particular patient's circumstances. As an extreme example, there may be a patient suffering from a serious or life-threatening disease who is warned of a certain defined risk of adverse reaction. Participation in the trial is then based on an expectation that the benefit/risk ratio associated with alternative treatment. It is, therefore, reasonable that the patient accepts the high risk and should not expect compensation for the occurrence of the adverse reaction of which he or she was told.

4.3 In any case where the company concedes that a payment should be made to a patient but there exists a difference of opinion between company and patient as to the appropriate level of compensation, it is recommended that the company agrees to seek at its own cost (and make available to the patient) the opinion of a mutually acceptable independent expert, and that his opinion will be given substantial weight by the company in reaching its decision on the appropriate payment to be made.

5. Miscellaneous

5.1 Claims pursuant to the Guidelines should be made by the patient to the company, preferably via the investigator, setting out details of the nature and background of the claim and, subject to the patient providing on request an authority for the company to review any medical records relevant to the claim, the company should consider the claim expeditiously.

5.2 The undertaking given by a company extends to injury arising (at whatever time) from all administrations, clinical interventions or procedures occurring during the course of the trial but not to treatment extended beyond the end of the trial at the instigation of the investigator. The use of unlicensed products beyond the trial period is wholly the responsibility of the treating doctor and in this regard attention is drawn to the advice provided to doctors in MAI 30[2] concerning the desirability of doctors notifying their protection society of their use of unlicensed products.

5.3 The fact that a company has agreed to abide by these Guidelines in respect of a trial does not affect the right of a patient to pursue a legal remedy in respect of injury alleged to have been suffered as a result of participation. Nevertheless, patients will normally be asked to accept that any payment made under the Guidelines will be in full settlement of their claims.

5.4 A company sponsoring a trial should encourage the investigator to make clear to participating patients that the trial is being conducted to the ABPI Guidelines relating to compensation for injury arising in the course of clinical trials and have available copies of the Guidelines should they be requested.

* Glaxo Research and Development Limited undertakes not to impose the limitation in Section 3.3

References

1. Guidelines for Medical Experiments in Non-patient Human Volunteers, ABPI March 1988, as amended May 1990.

2. MAI. 30 - A Guide to the Provisions affecting Doctors and Dentists, DHSS, (Revised June 1985).

Appendix N: PROJECT-SPECIFIC EXEMPTIONS TO SERIOUS ADVERSE EVENTS

The following conditions should not be recorded as Adverse Events or Serious Adverse Events unless they are fatal events:

AIDS dementia complex
Anognital warts (condyloma)
Bacterial septicemia or endocarditis
Candidiasis: bronchial, tracheal, lung, esophageal, oral, vaginal
Chancroid
Chlamydia
Coccidiodomycosis: disseminated, extrapulmonary, pulmonary
Community-acquired pneumonia
Cryptococcosis: extrapulmonary, pulmonary
Cryptosporidiosis: chronic intestinal
Cytomegalovirus disease: other than liver, spleen or nodes.
Cytomegalovirus retinitis (with loss of vision)
Encephalopathy (HIV-related)
Failure to thrive (patients <=12 years)
Gonorrhea
Hairy leukoplakia
Herpes simplex: chronic ulcers, bronchitis, pneumonitis, esophagitis
Herpes zoster: shingles, disseminated
Histoplasmosis: disseminated, extrapulmonary
Isosporiasis, chronic intestinal
Kaposi's sarcoma
Lymphoid interstitial pneumonitis (patients <=12 years)
Microsporidia, chronic colitis
Molluscum contagiosum
Mycobacterium avium complex: disseminated, extrapulmonary
Mycobacterium kansasii: disseminated, extrapulmonary
Mycobacterium tuberculosis
Mycobacterium other species or unidentified: disseminated, extrapulmonary, atypical
Otitis media
Pelvic inflammatory disease
Pneumocystis carinii pneumonia or extrapulmonary
Pneumonia, recurrent
Proctitis
Progressive multifocal leukoencephalopathy
Salmonella septicemia, recurrent

**Title of Protocol being Amended:**	A Phase III, Randomised, Double-Blind, Multicentre Study to Evaluate the Safety and Efficacy of 1592U89 in HIV-1 Infected Patients with AIDS Dementia Complex - Incorporating Amendment Number 4
**Number of Amendment and Date:**	Amendment Number 1: 25 September 1996

Protocol Amendment Number 1

This amendment consists of the following changes:

1. Replace Appendix L2 with a new version of the caregiver questionnaire.
2. Amend section 6.9 Adverse Experiences Reporting Requirements to reflect the new project guidelines.
3. Add Appendix N.

Signature of Investigator: ....

Name of Investigator:

Address of Site:

Signature on behalf of Sponsor: ....

Name:

Position in Company:

**Title of Protocol being Amended:**	A Phase III, Randomised, Double-Blind, Multicentre Study to Evaluate the Safety and Efficacy of 1592U89 in HIV-1 Infected Patients with AIDS Dementia Complex - Incorporating Amendment Number 4
**Number of Amendment and Date:**	Amendment Number 1: 25 September 1996

Change Number 1:

Replace Appendix L2 with a new version of the caregiver questionnaire.

Description of Change:

Please refer to Appendix L2.

Applies to:

Appendix L2.

Reason for Change:

The HIV-CIA (version 2.0) is a revised version of the earlier instrument chosen for this study and it is designed to be more sensitive to changes in patient functioning in patients with mild to moderate HIV related cognitive impairment.

Change Number 2:

Amend section 6.9 Adverse Experiences Reporting Requirements to reflect the new project guidelines

Description of Change:

6.9 Adverse Event Reporting

One of the aims of this study is to assess the safety and tolerability of the drug and therefore the investigator is responsible for recording and reporting adverse events observed during and after drug treatment. If treatment for an adverse event is being considered, the investigator should bear in mind that the new treatment may obscure the results of study drug withdrawal or discontinuation (dechallenge), or cause further adverse events.

**Title of Protocol being Amended:**	A Phase III, Randomised, Double-Blind, Multicentre Study to Evaluate the Safety and Efficacy of 1592U89 in HIV-1 Infected Patients with AIDS Dementia Complex - Incorporating Amendment Number 4
**Number of Amendment and Date:**	Amendment Number 1: 25 September 1996

6.9.1 *Recording*

The investigator should record all adverse events (see definitions below) and pregnancies. At each visit, after the patient has had an opportunity spontaneously to mention any problems, the investigator should enquire about adverse events by asking the following standard questions:

- "Have you had any (other) medical problems since your last visit/assessment?
- Have you taken any new medication, other than those given to you within this study, since your last visit/assessment?"

6.9.2 *Documentation*

All adverse events and pregnancies must be promptly documented by completing the adverse event forms supplied by Glaxo Wellcome. Their completeness and accuracy will be checked by the study monitor who visits the investigating centre.

6.9.3. *Definition of an Adverse Event (AE)*

An adverse event is defined as any untoward medical occurrence experienced by a patient. An event may consist of a disease, an exacerbation of a pre-existing illness, a recurrence of an intermittent illness, a set of related symptoms or signs or a single symptom or sign. A causal relationship to study drug is not necessarily implied.

6.9.4 *Definition of a Serious Adverse Event (SAE)*

A serious adverse event includes any experience or event that is:

a. **fatal**

b. **life-threatening (except if clearly related to HIV disease as listed in Appendix N)**

**Title of Protocol being Amended:**	A Phase III, Randomised, Double-Blind, Multicentre Study to Evaluate the Safety and Efficacy of 1592U89 in HIV-1 Infected Patients with AIDS Dementia Complex - Incorporating Amendment Number 4
**Number of Amendment and Date:**	Amendment Number 1: 25 September 1996

A life-threatening event is present when the subject was, in the view of the investigator, at immediate risk of death as the event occurred.

This definition does not include an event that, had it occurred in a more serious form, might have caused death.

c. **disabling or incapacitating (except if clearly related to HIV disease as listed in Appendix N)**

d. **requires in-patient hospitalisation (except if clearly related to HIV disease as listed in Appendix N)**

Hospitalisation for elective treatment of a pre-existing condition that did not worsen during the study is **not** considered an adverse event.

Complications which occur during hospitalisation are adverse events and if a complication prolongs hospitalisation then the event is serious.

e. **a congenital anomaly in the offspring of a patient who received drug (except if clearly related to HIV disease as listed in Appendix N)**

f. **cancer (except if clearly related to HIV disease as listed in Appendix N)**

g. **an event resulting from an overdose of the study drug**

   Overdose is defined as a single dose greater than the highest protocolled dose studied to date.

h. **an event which, though not included in (a) through (g) above, may jeopardise the patient or may require intervention to prevent one of the outcomes listed in (a) to (g), (except if clearly related to HIV disease as listed in Appendix N).**

i. **all grade 4 laboratory abnormalities, regardless of symptomatology are regarded as serious adverse events**

**Title of Protocol being Amended:**	A Phase III, Randomised, Double-Blind, Multicentre Study to Evaluate the Safety and Efficacy of 1592U89 in HIV-1 Infected Patients with AIDS Dementia Complex - Incorporating Amendment Number 4
**Number of Amendment and Date:**	Amendment Number 1: 25 September 1996

Laboratory abnormalities < grade 4 toxicity will not automatically be considered serious. However, if they meet any other of the SAE criteria they should be reported as SAEs.

6.9.4.1 Project-specific Exemptions from the AE and SAE Definition

The following events are not to be considered AEs or SAEs:

- **any condition which occurred prior to the patient being enrolled in the study and which did NOT worsen during the study**

These conditions should be recorded at screening.

The following HIV-related events are to be excluded from the definition of adverse events and serious adverse events and all of the associated reporting time frames (unless they are fatal), even if there is reason for their being classified as serious (e.g. hospitalisation, life-threatening etc):

- **any condition that is clearly related to HIV disease as listed in Appendix N. Lymphomas and invasive cervical carcinoma are excluded from this exemption; they must be reported even if they are considered to be HIV-related.**

Any of the HIV-related conditions which appear in Appendix N should be recorded on the "HIV-associated conditions" page in the CRF.

Fatal HIV-related events are not part of this exemption; they should be recorded and reported according to the criteria for SAEs.

6.9.4.2 Reporting

Life-threatening adverse events and deaths, whether causally related to treatment or not, must initially be notified by the investigator to Glaxo Wellcome within 24 hours. All data immediately available must be reported, preferably by completing and faxing the adverse event forms.

**Title of Protocol being Amended:**	A Phase III, Randomised, Double-Blind, Multicentre Study to Evaluate the Safety and Efficacy of 1592U89 in HIV-1 Infected Patients with AIDS Dementia Complex - Incorporating Amendment Number 4
**Number of Amendment and Date:**	Amendment Number 1: 25 September 1996

Telephone notification is required in the absence of facsimile facilities. Following the initial notification, the investigator must then record all additional information and fax or mail a copy of the completed SAE reporting forms within 48 hours.

For all other serious adverse events (see definition) and pregnancies the Serious adverse event form must be fully completed and a copy returned within 48 hours.

6.9.4. 3 Other Investigations

The investigator and others responsible for patient care should institute any supplementary investigations of serious adverse events based on their clinical judgement of the likely causative factors. This may include extra laboratory tests, histopathological examinations, or seeking a further opinion from a specialist in the field of the adverse event. Glaxo Wellcome may also request extra tests or extra follow-up information from the trialist. If a patient dies, any post-mortem findings including histopathology must be provided to Glaxo Wellcome.

6.9.4.4 Regulatory Aspects

Glaxo Wellcome has a legal responsibility to notify both the local regulatory authority and other overseas agencies about the safety of a drug under clinical investigation. Prompt notification of serious adverse events to the Glaxo Wellcome staff concerned, by the investigator, is essential so that legal obligations and ethical responsibilities towards the safety of other patients are met.

If there is difficulty in contacting the Glaxo Wellcome Personnel responsible for the study, information can be relayed through the Head of Clinical Studies Safety Management in International Product Safety & Pharmacovigilance, UK (0)181 422 3434.

In agreeing to the provisions of this protocol, the investigator accepts responsibility from prompt notification of SAEs to Glaxo Wellcome.

**Title of Protocol being Amended:**	A Phase III, Randomised, Double-Blind, Multicentre Study to Evaluate the Safety and Efficacy of 1592U89 in HIV-1 Infected Patients with AIDS Dementia Complex - Incorporating Amendment Number 4
**Number of Amendment and Date:**	Amendment Number 1: 25 September 1996

6.9.5 *Dose Modification due to Toxicity or Adverse Experience*

Adverse experiences which occur during the trial should be evaluated by the investigator and graded according to the ACTG toxicity scales (see Appendix J).

Study treatment dosing may be modified at the discretion of the investigator and according to the severity of the adverse experience. All dose modifications must be accurately recorded on the study drug pages of the case report forms. If treatment for an adverse event is being considered the investigator should bear in mind that the new treatment may obscure the results of study drug withdrawal or discontinuation (dechallenge), or cause further adverse events.

Should the investigator have evidence that the adverse experience is not caused by study medication, dosage may then remain unaltered. However, if the attributability of the AE cannot be ascertained dose modifications should be instigated.

Subjects requiring a dose modification must be re-evaluated on a weekly basis.

6.9.5.1 *Grade 1 Toxicity/Adverse Experience*

Subjects who develop a grade 1 adverse experience may continue therapy without alteration of the study treatment.

6.9.5.2 *Grade 2 Toxicity/Adverse Experience*

Subjects who develop a grade 2 adverse experience may continue therapy at the discretion of the investigator.

**Title of Protocol being Amended:**	A Phase III, Randomised, Double-Blind, Multicentre Study to Evaluate the Safety and Efficacy of 1592U89 in HIV-1 Infected Patients with AIDS Dementia Complex - Incorporating Amendment Number 4
**Number of Amendment and Date:**	Amendment Number 1: 25 September 1996

6.9.5.3 *Grade 3 Toxicity/Adverse Experience*

Subjects who develop a grade 3 adverse experience should have their study medication withheld until the AE diminishes to ≤ grade 1 for one week. At that time, subject may be re-challenged with 50% of study dosing. If the AE remains at ≤ grade 1 for two weeks, full treatment may be reinstated.

Should the same grade 3 adverse experience recur within 4 weeks in the same subject, the study medication should be permanently discontinued. Patients experiencing recurrence of haematological toxicity may remain on study at the discretion of the investigator and under strict clinical management.

Note : The aetiology of neuropathies may be difficult to ascertain, therefore study treatment should initially be withheld. Should the neuropathy not improve within one week, study treatment may be reinstated at the discretion of the investigator. The end-point committee must be notified.

6.9.5.4 *Grade 4 Toxicity/Adverse Experience*

Subjects who develop a grade 4 adverse experience should have their study medication permanently discontinued. The subject should be followed weekly until resolution of the adverse experience and encouraged to complete the protocolled study evaluations. Grade 4 neuropathies may be handled as above.

Applies to:

Section 6.9.

Reason for Change:

All new and continuing protocols within the 1592U89 project must reflect the project agreed SAE reporting guidelines which complies with the current Glaxo Wellcome AE reporting guidelines.

**Title of Protocol being Amended:**	A Phase III, Randomised, Double-Blind, Multicentre Study to Evaluate the Safety and Efficacy of 1592U89 in HIV-1 Infected Patients with AIDS Dementia Complex - Incorporating Amendment Number 4
**Number of Amendment and Date:**	Amendment Number 1: 25 September 1996

Change Number 3:

Add Appendix N.

Description of Change:

Please refer to Appendix N.

Applies to:

Appendix N.

Reason for Change:

It details the project specific exemptions referred to on section 6.9.

**Title of Protocol being Amended:**	A Phase III, Randomised, Double-Blind, Multicentre Study to Evaluate the Safety and Efficacy of 1592U89 in HIV-1 Infected Patients with AIDS Dementia Complex - Incorporating Amendment Number 4
**Number of Amendment and Date:**	Amendment Number 2<br>17 February 1997

Protocol Amendment Number 2

This amendment consists of the following changes:

1. Amend summary section, sections 3 (Study Design) and 5.5 (Concomitant Medication) to reflect change in the use of d4T during the 8 week pre-entry period and randomised phase of the study.

2. Amend section 3, 4.2 and 6.4 and introduce sections to reflect clarification on the documentation required to confirm ADC diagnosis/progression.

3. Include new sections 6.13 and 6.14 on the validation of Dementia End-points and Clinical HIV End-points and re-number current sections 6.13 and thereafter appropriately.

4. Amend section 4.2, exclusion criteria C to allow enrolment of subjects with a Karnofsky score of 60.

5. Amend section 4.1, inclusion criteria E to reflect the substitution of the word "tests" for "domains".

6. Amend section 1.3.3 to reflect the addition of 6 month animal toxicological data in the Clinical Investigators Brochure.

7. Amend section 6.9.4, point "g" to include definition of overdose.

**Title of Protocol being Amended:**	A Phase III, Randomised, Double-Blind, Multicentre Study to Evaluate the Safety and Efficacy of 1592U89 in HIV-1 Infected Patients with AIDS Dementia Complex - Incorporating Amendment Number 4
**Number of Amendment and Date:**	Amendment Number 2<br>17 February 1997

Signature of Investigator: ....

Name of Investigator:

Address of Site:

Signature on behalf of Sponsor: ....

Name:

Position in Company:

**Title of Protocol being Amended:**	A Phase III, Randomised, Double-Blind, Multicentre Study to Evaluate the Safety and Efficacy of 1592U89 in HIV-1 Infected Patients with AIDS Dementia Complex - Incorporating Amendment Number 4
**Number of Amendment and Date:**	Amendment Number 2<br>17 February 1997

Change Number 1:

Amend Summary Section, Sections 3 (Study Design) and 5.5 (Concomitant Medication) to reflect change in the use of d4T during the 8 week pre-entry period and randomised phase of the study

Description of Change:

Summary section, 4th paragraph: Delete the sentence "Concurrent use of d4T and"

To read:

"Change or addition of background antiretroviral agents will not be permitted during the 8 week pre-entry period or the randomised phase of the study"

Section 3 Study Design, 1st Paragraph: delete the last sentence: "Concurrent use of d4T will not be permitted during the 8 week pre-entry period or the randomised phase of the study due to available *in vitro* data suggesting antagonistic activity with 1592U89"

To read:

"This is a phase III randomised, double blind, multicentre study to evaluate..........Agents available by expanded access or other parallel track programs will be assessed on a case-by-case basis with the sponsor"

Section 4.4 Subject Stratification and Randomisation: add at the end of the first paragraph "Subjects receiving d4T will be stratified into group B."

**Title of Protocol being Amended:**	A Phase III, Randomised, Double-Blind, Multicentre Study to Evaluate the Safety and Efficacy of 1592U89 in HIV-1 Infected Patients with AIDS Dementia Complex - Incorporating Amendment Number 4
**Number of Amendment and Date:**	Amendment Number 2<br>17 February 1997

To read:

"Subjects will be stratified during the pre-entry period into group A if their existing therapy contains zidovudine or into group B if they are not taking zidovudine in their antiretroviral regimen. Subjects receiving d4T will be stratified into group B."

Section 5.5 Concomitant Medication: delete 5th paragraph.

Applies to:

Summary section, sections 3 (Study Design), 4.4 (Subject Stratification and Randomisation) and 5.5 (Concomitant Medication)

Reason for Change:

Results from repeated in vitro experiments indicate that there is no antagonism between d4T and 1592U89. Preliminary in vivo data also suggest 1592U89 may provide additional benefit to d4T pre-treated patients. It is expected that allowing d4T as part of the standard of care treatment will improve study recruitment, given the widespread use of this drug among the study population.

Change Number 2:

Amend Sections 3, 4.2, and 6.4 to reflect clarification on the documentation required to confirm ADC diagnosis/progression.

Description of Change:

Amend section 3, 5th paragraph, second sentence: "To confirm such deterioration is caused by HIV-1, any CNS opportunistic infections or other conditions will be excluded by CT or MRI scans and/or any other required supporting tests."

**Title of Protocol being Amended:**	A Phase III, Randomised, Double-Blind, Multicentre Study to Evaluate the Safety and Efficacy of 1592U89 in HIV-1 Infected Patients with AIDS Dementia Complex - Incorporating Amendment Number 4
**Number of Amendment and Date:**	Amendment Number 2<br>17 February 1997

To read:

To confirm such deterioration is caused by HIV-1, any CNS opportunistic infections or other conditions will be excluded by:

- MRI scan (or CT scan with contrast if MRI scanner facilities are not available to the site)
- CSF analysis (as detailed in Appendix F2) and/or
- Any other required supporting tests"

Amend section 4.2, exclusion criteria Dii

To read:

"Patients who have current CNS infections or neoplasms as revealed by MRI scan (CT scan with contrast if MRI scanner facilities not available) and CSF analysis" (as detailed in appendix F2)

Section 6.4, 1st paragraph, last sentence: delete "at the discretion of the investigator"

To read:

"Additional assessments may be performed throughout the study if deemed necessary by the investigator e.g. ADC progression and adverse experiences. The following evaluations should be conducted:"

Section 6.4, Clinical Evaluations: modify 8th bullet point

To read :

Magnetic Resonance Imaging Scan (or CT scan with contrast if MRI scanner facilities not available)

Section 6.4, Laboratory Evaluations, 4th bullet point: delete "(if deemed necessary)"

**Title of Protocol being Amended:**	A Phase III, Randomised, Double-Blind, Multicentre Study to Evaluate the Safety and Efficacy of 1592U89 in HIV-1 Infected Patients with AIDS Dementia Complex - Incorporating Amendment Number 4
**Number of Amendment and Date:**	Amendment Number 2<br>17 February 1997

To read:

"Exclusion Diagnosis CSF Analysis (as detailed in appendix F2)

Appendix A

Footnote "d": delete "at the discretion of the investigators"

To read:

"To be performed in cases of suspected ADC progression"

Footnote "h" modify

To read:

"To be performed if circumstances permit, in cases where a lumbar puncture is required as part of clinical management or end-point validation"

Page 45, CSF Analysis (Exclusion Diagnosis) at Extra Assesments: change footnote "h" to read footnote "d"

Applies to:

Sections 3, 4.2, 6.4 and Appendix A

Reason for Change:

Changes described will clarify the documentation required for the validation of Dementia End-points.

**Title of Protocol being Amended:**	A Phase III, Randomised, Double-Blind, Multicentre Study to Evaluate the Safety and Efficacy of 1592U89 in HIV-1 Infected Patients with AIDS Dementia Complex - Incorporating Amendment Number 2
**Number of Amendment and Date:**	Amendment Number 2<br>17 February 1997

Change Number 3:

Include new sections 6.13 and 6.14 on the validation of Dementia End-points and Clinical HIV End-points and re-number current sections 6.13. and thereafter appropriately

Description of Change:

Replace current section 6.13.

To read:

6.13 **Validation of Dementia End-Points**

For the purpose of this protocol, a dementia end-point is defined as progression by at least one stage within the MSK scale for ADC. At the time the subject is considered to have met the end-point, an exclusion diagnosis, confirmed by MRI Scan, CSF analysis and any other supporting documentation, will be required.

Given the subjectivity involved in the assessment and staging of ADC, an end-point committee comprised by four participating investigators (see appendix K), has been established to independently review all dementia end-points occurred during the study. To ensure an independent assessment, the origin of the site or investigator producing the end-point, will not be revealed to the committee. Similarly, any end-points arising from the site of any of the committee members will not be reviewed by the member in question.

The validation of the dementia end-points will be co-ordinated through the GW study co-ordinator. Further details of the process for confirmation of end-points can be found in the study procedures manual.

**Title of Protocol being Amended:**	A Phase III, Randomised, Double-Blind, Multicentre Study to Evaluate the Safety and Efficacy of 1592U89 in HIV-1 Infected Patients with AIDS Dementia Complex - Incorporating Amendment Number 2
**Number of Amendment and Date:**	Amendment Number 2<br>17 February 1997

Replace current section 6.14

<u>To read:</u>

6.14 Validation of HIV Clinical End-Points

Systemic clinical end-points will be analysed as part of the secondary objectives of this study. All AIDS defining events occurring during both phases of the study (randomised and open), will be validated by an external expert physician.

A section of the study procedures manual will be dedicated to define standardised criteria which must be met for the diagnosis of all AIDS events which develop during the study. In addition, a list of the diagnostic procedures which should be used and the documentation which should be available to support the unequivocal diagnosis of a particular AIDS event will be provided. Please refer to the procedures manuals for further details.

Re-number sections currently numbered 6.13 and thereafter

<u>Applies to</u>:

Sections 6.13 and thereafter up to section 7

<u>Reason for Change</u>:

In order to highlight the validation process for dementia and clinical HIV end-points in the study, a dedicated section has been added to the protocol.

**Title of Protocol being Amended:**	A Phase III, Randomised, Double-Blind, Multicentre Study to Evaluate the Safety and Efficacy of 1592U89 in HIV-1 Infected Patients with AIDS Dementia Complex - Incorporating Amendment Number 2
**Number of Amendment and Date:**	Amendment Number 2<br>17 February 1997

Change Number 4:

Amend section 4.2, exclusion criteria C to allow enrolment of subjects with a Karnofsky score of 60

Description of Change:

Section 4.2 : Amend Exclusion Criteria C

To read:

"Patients with a Karnofsky score < 60 (Appendix C)"

Applies to:

Section 4.2 Exclusion Criteria C

Reason for change

It is believed that prospective subjects meeting ADC stages 1 & 2 and Neuropsychological criteria are being excluded from the study due to their neurological abnormalities which may bias their Karnofsky Score negatively.

**Title of Protocol being Amended:**	A Phase III, Randomised, Double-Blind, Multicentre Study to Evaluate the Safety and Efficacy of 1592U89 in HIV-1 Infected Patients with AIDS Dementia Complex - Incorporating Amendment Number 2
**Number of Amendment and Date:**	Amendment Number 2<br>17 February 1997

Change Number 5:

Amend Section 4.1, inclusion criteria E to reflect the substitution of the word "domains" for "tests"

Description of Change:

Section 4.1 : Amend Inclusion Criteria E

To read:

"Patients will be required to be impaired by 1.5 Standard Deviations below normal in at least two neuropsychological domains from the battery described in Appendix D."

Applies to:

Section 4.1, Inclusion Criteria E

Reason for change

The use of the word "domains" is required due to the fact that some tests from the battery used, such as the grooved pegboard dominant versus non-dominant hand, examine the same domain.

**Title of Protocol being Amended:**	A Phase III, Randomised, Double-Blind, Multicentre Study to Evaluate the Safety and Efficacy of 1592U89 in HIV-1 Infected Patients with AIDS Dementia Complex - Incorporating Amendment Number 2
**Number of Amendment and Date:**	Amendment Number 2<br>17 February 1997

Change Number 6:

Amend Section 1.3.3 to reflect the addition of 6 month animal toxicological data in the Investigators Brochure.

Description of Change:

Modify section 1.3.3 Toxicology, last paragraph

To read:

Results from the six month toxicology studies are now availble in our updated version of the clinical investigators brochure dated 30th July 1996. Results on the 12 month studies in cynomolgus monkeys are expected to be available in Q2, 1997.

Applies to:

Section 1.3.3 Toxicology

Reason for change

Update on the availability of animal toxicological studies.

**Title of Protocol being Amended:**	A Phase III, Randomised, Double-Blind, Multicentre Study to Evaluate the Safety and Efficacy of 1592U89 in HIV-1 Infected Patients with AIDS Dementia Complex - Incorporating Amendment Number 2
**Number of Amendment and Date:**	Amendment Number 2<br>17 February 1997

Change Number 7:

Amend Section 6.9.4 point "g" to include definition of overdose.

Description of Change:

Modify section 6.9.4 point "g", 2nd sentence

To read:

"Overdose is defined as the ingestion of at or above 1200mg/dose of the study drug (1592U89)."

Applies to:

Section 6.9.4.

Reason for change

Definition of overdose was inadvertently omitted from the protocol when the first amendment was completed.

A Phase III, Randomised, Double-Blind, Multicentre Study to Evaluate the Safety and Efficacy of 1592U89 in HIV-1 Infected Patients with AIDS Dementia Complex - Incorporating Amendment Number 4

Protocol Number CNAB3001

PROTOCOL AMENDMENT 3 - 13 June 1997

Protocol Amendment Number 3

This amendment consists of the following changes:

1. **Amend Section, 5.1 (Study Drug) to incorporate change in study drug formulation (300mg 1592 hemisulphate tablets).**

2. **Amend Section 1.3.4 (Clinical Studies) and create section 1.3.4.3 titled "Clinical Experience in nRTI-Experienced Adults" to incorporate new information recently available**

Signature of Investigator: ....

Name of Investigator:

Address of Site:

Signature on behalf of Sponsor: ....

Name:

Position in Company:

A Phase III, Randomised, Double-Blind, Multicentre Study to Evaluate the Safety and Efficacy of 1592U89 in HIV-1 Infected Patients with AIDS Dementia Complex - Incorporating Amendment Number 4

Protocol Number CNAB3001

PROTOCOL AMENDMENT 3 - 13 June 1997

CHANGE NUMBER 1:

1. **Amend Section, 5.1 (Study Drug) to incorporate change in study drug formulation (300mg 1592 hemisulphate tablets).**

Description of change

Section 5.1, 1st paragraph at the end of the sentence: delete full stop and add sentence "and bottles of 70 tablets of 300mg. The 300mg formulation will be provided for open supplies only, whilst the randomised treatment will continue to be provided as 100mg tablets. To facilitate the switch of the two formulations, patients will be asked to finish their existing 100mg tablets supplies. The patient's information will be amended to reflect this change, however, site personnel responsible for the dispensing of study drug, must ensure that both the patient and the patient's caregiver are fully informed of this change in dose administration to avoid the risk of study drug overdose.

To read

"1592U89 will be supplied by Glaxo Wellcome in bottles of 100mg and bottles of 70 tablets of 300mg. The 300mg formulation will be provided for open supplies only, whilst the randomised treatment will continue to be provided as 100mg tablets. To facilitate the switch of the two formulations, patients will be asked to finish their existing 100mg tablets supplies. The patient's information document will be amended to reflect this change, however, site personnel responsible for the dispensing of study drug, must ensure that both the patient and the patient's caregiver are fully informed of this change in dose administration to avoid any risk of overdose."

Applies to:

Section 5.1

Reason for change

Changes reflect the introduction of new formulation which will reduce number of tablets to be taken. Information about the 300mg 1592U89

hemisulphate formulation and pre-clinical bioequivalence of the two forms of 1592U89 is available in the current Clinical Investigators Brochure dated 14th March 1997, under sections 3.2 (page 10) and 4.4.25 (page 47) respectively.

2. Amend Section 1.3.4 (Clinical Studies) and create section 1.3.4.3 titled "Clinical Experience in nRTI-Experienced Adults" to incorporate new information recently available

Description of change

Add section 1.3.4.3

To read:

Clinical Experience in nRTI-Experienced Adults

There has been very limited experience with the use of 1592 in patients previously treated with other anti-retroviral agents; especially nucleoside reverse transcriptase inhibitors (nRTI). Fifty two (52) patients who participated in CNAA2001 (a phase 2 dose-escalating study of 1592 and AZT/1592 in treatment naive adults) and discontinued 1592 therapy after completing the 12 week treatment phase of the protocol have reinstated 1592 therapy after a 1-6 month interim (treatment discontinuation required until adequate pre-clinical safety data were available to support longer term treatment in humans). During this interim, patients were encouraged to receive alternative anti-retroviral therapy with commercially available agents. Preliminary data are available from 15 patients who re-started 1592 treatment in addition to continuing their other anti-retroviral agents. The type of interim anti-retroviral treatment which these patients received was quite variable with the most common treatment chosen (6 patients) being AZT/3TC. At week 4 after re-starting 1592 treatment, 14 of these 15 patients (93%) had plasma HIV RNA below the limits of detection of the assay (400 c/ml; Roche Amplicor). At week 12 after re-starting 1592 treatment, 8 of 9 patients (89%) had plasma HIV RNA below the limits of detection of the assay. These data suggest that patients treated with other ant-retroviral agents for period of up to 6 months continue to experience additional anti-retroviral activity when 1592 is added to their current treatment regimen.

A Phase III, Randomised, Double-Blind, Multicentre Study to Evaluate the Safety and Efficacy of 1592U89 in HIV-1 Infected Patients with AIDS Dementia Complex - Incorporating Amendment Number 4
Protocol Number CNAB3001

PROTOCOL AMENDMENT 3 - 13 June 1997

A phase II study (protocol CNAA2003) is currently being conducted to evaluate the anti-retroviral effect of 1592 in patients who have received long-term nRTI therapy and are currently failing this treatment regimen (e.g. plasma HIV RNA >10,000 c/ml). This population was studied to assess the effect of 1592 in patients who probably harbor nRTI-resistant virus (e.g. resistant to one or more nRTI). Forty patients were intended to be enrolled in this protocol, however, enrollment was discontinued after 33 patients had been enrolled due to difficulties in identifying patients who met the strict inclusion criteria regarding prior treatment history. Patients were eligible for enrollment if they met one of the following treatment history criteria: 1) $\geq$6 months d4T monotherapy, 2) $\geq$6 months ddI ($\pm$ZDV) therapy, 3) $\geq$12 months ZDV monotherapy, or 4) $\geq$12 months ZDV/3TC therapy. These prior treatments were chosen to assure that the patient population studied would include a broad distribution of nRTI experience and potential resistance. All patients received 1592 300 mg BID for up to 24 weeks in addition to their current anti-retroviral therapy.

Preliminary virology data from 23 patients in this study are available for the first 4 weeks of treatment with 1592. Median baseline plasma viral load in these 23 patients was 4.70 $\log_{10}$ c/ml (range 3.07 to 5.83 $\log_{10}$ c/ml) Addition of 1592 to current treatment in these patients resulted in an additional median 1.02 $\log_{10}$ c/ml decrease in plasma HIV RNA (range 0.42 increase to -3.15 decrease) at week 4 of treatment. While this median decrease in plasma HIV RNA in this patient population is quite impressive, especially for a patient population that was failing their prior treatment regimen, a detailed analysis of individual patient data indicated a variable response to 1592 treatment. For example, 10 of 23 patients (43%) had a decrease in plasma HIV RNA of <0.5 $\log_{10}$ c/ml. However, 5 of 23 patients (21%) had a decrease in plasma HIV RNA to below the limits of detection of the assay (<400 c/ml; Roche Amplicor assay). While analysis of virologic genotypic and phenotypic data and viral load changes from this study are still incomplete, it appears that the patients in this study with prior long term exposure to multiple ($\geq$2) nRTI therapies (and who are failing treatment probably due to the development of resistance to these agents as implied by a high viral load) tended to exhibit an attenuated response to the addition of 1592 to existing therapy. Viral isolates from these patients demonstrated multiple genotype changes associated with resistance to a number of nRTI. The implications of these data are not currently clear although it suggests that patients who develop viral resistance

to multiple nRTI may exhibit only limited anti-retroviral response to 1592 when administered alone. This study does not give us any information on the contribution of 1592 in combination with other new agents (agents that the patient has never received) in which other factors such as synergy may play a role in the activity of the new regimen. A more detailed analysis of the data from this study will be conducted after the completion of the study. Also, additional studies are planned or ongoing to better define the benefit of 1592 treatment in nRTI-experienced patients.

Applies to:

Section 1.3.4

Reason for change

This section provides emerging information about 1592U89 which the company considers relevant to inform investigators and subjects participating in 1592U89 trials.

A Phase III, Randomised, Double-Blind, Multicentre Study to Evaluate the Safety and Efficacy of 1592U89 in HIV-1 Infected Patients with AIDS Dementia Complex - Incorporating Amendment Number 4

Protocol Number CNAB3001

PROTOCOL AMENDMENT 4 - Date 28 August 1997

Protocol Amendment Number 4

This amendment consists of the following changes:

1. Amend section 1.3.3 to reflect availability of 12 month toxicology studies included in our latest CIB (Clinical Investigators Brochure) version

2. Amend section 5.5 to include additional information on potential pharmacokinetic interactions of 1592 with other drugs.

3. Amend section 5.5 to ease restrictions on the use of concomitant medication during the open label phase.

4. Amend Section 6.6 (Follow-up Evaluation) Laboratory Evaluations to include Serum β-HCG testing for females of childbearing potential.

5. Amend Section 6.9 to identify AE reporting during screening phase.

6. Amend Section 6.9.2. to reflect the introduction of new forms to report pregnancies on study.

7. Amend Section 6.9.3 to update to latest GW standard definition of AE.

8. Amend Section 6.9.4.2 to delete "pregnancies" to reflect change in the different reporting requirements of pregnancies and SAEs.

9. Insert new section "Pregnancy Reporting" as section 6.9.4.4 and re-number current section 6.9.4.4 to 6.9.4.5.

10. Amend Section 6.9.5 and insert a new section: "6.9.5.5 Handling of Rash/Allergic Reaction Episodes".

11. Amend Section 6.13. to include the validation of "positive" ADC end-points.

12. Append Section 9. EXTENSION PHASE

13. Insert Appendix A1 (Extension Phase Flow Chart) after Appendix A.

14. Amend Appendix K to reflect change No.9 from this amendment.

15. Amend Appendix J1 to modify the toxicity grading values for triglycerides.

A Phase III, Randomised, Double-Blind, Multicentre Study to Evaluate the Safety and Efficacy of 1592U89 in HIV-1 Infected Patients with AIDS Dementia Complex - Incorporating Amendment Number 4

Protocol Number CNAB3001

PROTOCOL AMENDMENT 4 - Date 28 August 1997

Signature of Investigator: ....

Name of Investigator:

Date: ....

Address of Site:

Signature on behalf of Sponsor: ....

Name:

Date: ....

Position in Company:

A Phase III, Randomised, Double-Blind, Multicentre Study to Evaluate the Safety and Efficacy of 1592U89 in HIV-1 Infected Patients with AIDS Dementia Complex - Incorporating Amendment Number 4

Protocol Number CNAB3001

PROTOCOL AMENDMENT 4 - Date 28 August 1997

CHANGE NUMBER 1:

1. **Amend section 1.3.3 to reflect availability of 12 month toxicology studies included in our latest CIB (Clinical Investigators Brochure) version**

<u>Description of change</u>

- Section 1.3.3, last paragraph, change from:

"Results from the six month toxicology studies are now available in our updated version of the clinical investigators brochure dated 30th July 1996. Results on the 12 month studies in cynomolgus monkeys are expected to be available in Q2, 1997."

to read:

"Results from the six **and twelve** month toxicology studies are now available in our updated version of the clinical investigators brochure dated 14th March 1997."

<u>Applies to:</u>

Section 1.3.3 "Toxicology Studies"

<u>Reason for change</u>

To reflect the availability of 12 month toxicology studies data, included in our latest CIB version.

CHANGE NUMBER 2:

2. **Amend section 5.5 to include additional information on potential pharmacokinetic interactions of 1592 with other drugs.**

Description of change:

- Section 5.5 page 19, delete the last two paragraphs of the section, currently reading:

"...1592U89 is metabolized by a cystolic alcohol dehydrogenase. Alcohol and any agents that inhibit this enzyme may affect the metabolism of 1592U89. Caution should be taken in the co-administration of 1592U89 and these agents.

Given the investigational stage of this compound, no specific contraindications to 1592U89 are known. However caution should be exercised with the use of drugs known to affect renal tubular secretion (such as probenecid or cimetidine), cause liver toxicity or induce myelosuppression."

and replace with the following text **to read:**

"In humans, the 5′-carboxylic acid and the 5′-glucuronide are the major metabolites measured in plasma. Preliminary results from a single dose study in adults indicates that approximately 35% of a 300mg dose of 1592 is eliminated in the urine as the carboxylic acid metabolite and 26% as the 1592U89-glucuronide metabolite. Less than 3% is excreted unchanged in the urine, and oxidative metabolites of 1592U89 account for minor biotransformation products. *In vitro* experiments indicate that cystolic alcohol dehydrogenase (ADH) and UDP-glucuronyl transferase (UDPGT) are the enzymes responsible for the formation of the 5′-carboxylic acid and the 5′-glucuronide metabolites of 1592U89, respectively. At a clinically relevant concentration, *in vitro* experiments reveal that 1592U89 is not a substrate nor does it inhibit CYP3A4 activity.

To date, three formal drug interaction studies have been initiated for 1592U89. A single-dose drug interaction study has been conducted for 1592U89 in combination with ZDV and 3TC. No clinically significant interactions were observed and the study is summarized in the Investigator's Brochure. An ethanol interaction study has concluded its clinical phase and samples are being assayed. The third study is a methadone interaction study which has just begun enrolment. Other larger scale clinical studies are collecting population pharmacokinetic samples to screen for drug interactions affecting 1592U89.

Based upon the known metabolism of 1592U89, the following table has been constructed and highlights potential drug-drug interactions. Compounds with the potential for interaction with 1592U89 are

identified as co-substrates or inducers of the ADH and UDPGT metabolic pathways. Because 1592U89 metabolism predominately occurs by two distinct pathways, and thus an alternate metabolic route is available, the potential for an increase in 1592U89 concentration is less likely when concomitantly administered with a co-substrate for one metabolic pathway. However, an increase in plasma concentration of the co-substrate may result upon concomitant administration with 1592U89. The scientific literature on the metabolism of the following UDPGT substrates is being searched to determine whether or not UDPGT acts on the parent substrate or on a secondary metabolite. If it acts on the parent substrate then there is a potential for increases in the parent concentrations while if it acts on the secondary metabolite then the potential increases would be in the metabolite concentrations. Compounds known to induce UDPGT enzymes may cause the plasma concentrations of 1592U89 to decrease and thereby compromise the optimal therapeutic effect.

Potential increase in concentrations of a concomitant medication (Co-Substrates)					
ADH Substrates		UDPGT Substrates			
alcohol	isoniazid	imipramine	chlorpromazine	lamotrigine	morphine
chlorzoxazone	chloral hydrate	amitriptyline	promethazine	valproic acid	dapsone
chlorpromazine		doxepin	chlorpheniramine	acetaminophen	propofol
		propranolol	labetalol	NSAIDS	temazepam
		oxazepam	naloxone	bumetanide	ketoconazole
		miconazole	fluconazole		
Potential decrease in 1592U89 concentrations (UDPGT Inducers)					
phenytoin	clofibrate	phenobarbital	cigarette smoke	isoniazid	T3-thyroid hormone

- If a patient is taking one of the above co-therapies in the co-substrate categories and is not experiencing an adverse reaction or change in a pre-existing adverse reaction, then there is no reason to alter therapy. If, on the other hand, a patient is experiencing an increase in adverse reactions with the co-therapy, then a reduction in the dose of the co-therapy should be considered.

- If a patient is taking one of the above co-therapies in the inducer category, then the potential interaction would be to decrease 1592U89 exposure and potentially, efficacy.

To date, no specific contra-indications to 1592U89 have been identified. The listing above is produced in the interest of completeness to highlight potential interferences that could theoretically occur. In this light, it is advised to exercise cautious monitoring when co-administering drugs known to cause liver toxicity or induce myelosuppression.

Applies to:

Section 5.5. "Concomitant Medication".

Reason for change:

To provide recently available information about the metabolism of 1592U89 and potential drug interactions.

CHANGE NUMBER 3:

3. **Amend section 5.5 to ease restrictions on the use of concomitant medication during the open label phase.**

Description of change:

- Section 5.5, 1st paragraph, 7th line, delete "study period" and replace with "randomised phase of the study"

  to read:

  "...Immunomodulating agents such as systemic corticosteroids, interleukins, thalidomide, anti-cytokine agents, or interferons, cytotoxic chemotherapeutic agents, anti-oxidants and radiation therapy (with the exception of local KS treatment) must not be used throughout the **randomised phase of the study.....**"

Applies to:

Section 5.5 "Concomitant Medication"

Reason for change:

The advanced stage of disease of the study population may require use of such concomitant medication. Use of this medication does not have a significant effect on the open label phase data analysis.

A Phase III, Randomised, Double-Blind, Multicentre Study to Evaluate the Safety and Efficacy of 1592U89 in HIV-1 Infected Patients with AIDS Dementia Complex - Incorporating Amendment Number 4
Protocol Number CNAB3001

PROTOCOL AMENDMENT 4 - Date 28 August 1997

CHANGE NUMBER 4:

4. **Amend Section 6.6, Laboratory Evaluations to include Serum β-HCG testing for females of childbearing potential at the Follow-up (off-drug) Evaluation**

Description of change:

- Section 6.6., page 25, Laboratory Evaluations please add bullet point titled: "Serum β-HCG testing for females of childbearing potential" before the "Haematology" bullet point.

Applies to:

Section 6.6 "Follow-up (Off-drug) Evaluation, Laboratory Evaluations"

Reason for change:

To conform with latest GW guidelines on pregnancy testing in clinical trials

CHANGE NUMBER 5:

5. **Amend Section 6.9 to identify AE reporting during screening phase**

Description of change:

- Section 6.9, 3rd line, change from:

  "...adverse events observed during and after drug treatment. If treatment for an adverse event ....."

  to read:

  "...adverse events observed during **(commencing from Day 1)** and after drug treatment **(up to the last follow-up visit in the study). In addition, serious adverse events related to study participation only should be reported in the screening phase.** If treatment for an adverse event ....."

A Phase III, Randomised, Double-Blind, Multicentre Study to Evaluate the Safety and Efficacy of 1592U89 in HIV-1 Infected Patients with AIDS Dementia Complex - Incorporating Amendment Number 4
Protocol Number CNAB3001

PROTOCOL AMENDMENT 4 - Date 28 August 1997

Applies to:

Section 6.9 "Adverse Event Reporting".

Reason for change:

To clarify adverse event reporting during the screening phase of the study.

CHANGE NUMBER 6:

6. **Amend Section 6.9.2 to reflect the introduction of new forms to report pregnancies on study.**

Description of change:

- Section 6.9.2, 2nd line, delete the word "forms" and replace with: "and pregnancy notification forms respectively"

  to read:

  "All adverse events and pregnancies must be promptly documented by completing the adverse event **and pregnancy notification forms respectively** supplied by Glaxo Wellcome...."

Applies to:

Section 6.9.2 "Documentation".

Reason for change:

To reflect the introduction of new GW forms to report pregnancies on study.

CHANGE NUMBER 7:

7. **Amend Section 6.9.3 to update to latest GW standard definition of AE**

Description of change:

- Section 6.9.3 change the entire section from:

"An adverse event is defined as any untoward medical occurrence experienced by a patient. An event may consist of a disease, an exacerbation of a pre-existing illness, a recurrence of an intermittent illness, a set of related symptoms or signs or a single symptom or sign. A causal relationship to study drug is not necessarily implied."

to read:

"An adverse event is defined as any untoward medical occurrence in a patient or clinical investigation subject administered a pharmaceutical product which does not necessarily have a causal relationship with this treatment. An adverse event (AE) can therefore be any unfavourable and unintended sign (that could include a clinically signficant abnormal laboratory finding), symptom, or disease temporally associated with the use of a medicinal product, whether or not considered related to the medicinal product.

An adverse event does include:

- An exacerbation of a pre-existing illness.
- Increase in frequency or intensity of a pre-existing episodic event or condition.
- A condition detected or diagnosed after trial medication administration even though it may have been present prior to the start of the study.
- Continuous persistent disease/symptoms present at baseline that worsen following the start of the study.

An adverse event does not include:

- Medical or surgical procedures (e.g., surgery, endoscopy, tooth extraction, transfusion); the condition that leads to the procedure is an AE.
- Pre-existing disease or conditions present or detected at the start of the study that do not worsen.

- Situations where an untoward medical occurrence has not occurred (e.g., hospitalisations for cosmetic elective surgery/social/ convenience admissions).
- The disease being studied or sign/symptoms associated with the disease unless more severe than expected for the subject's condition.
- Overdose of either trial medication or concurrent medication without any signs or symptoms."

Applies to:

Section 6.9.3. "Definition of an Adverse Event (AE)"

Reason for change:

To update to the latest GW standard definition of AE

CHANGE NUMBER 8:

8. **Amend Section 6.9.4.2 to delete "pregnancies" to reflect change in the different reporting requirements of pregnancies and SAEs**

Description of change:

- Section 6.9.4.2, 3rd parragraph, 1st line, please delete "and pregnancies"

  to read:

  "For all other serious adverse events (see definition), the serious adverse event form must be fully completed and a copy returned within 48 hours"

Applies to:

Section 6.9.4.2 "Reporting"

Reason for change:

To reflect recent change in the reporting requirements of pregnancies and SAEs

A Phase III, Randomised, Double-Blind, Multicentre Study to Evaluate the Safety and Efficacy of 1592U89 in HIV-1 Infected Patients with AIDS Dementia Complex - Incorporating Amendment Number 4
Protocol Number CNAB3001

PROTOCOL AMENDMENT 4 - Date 28 August 1997

CHANGE NUMBER 9:

9. **Insert new section "Pregnancy Reporting" as section 6.9.4.4 and renumber current section 6.9.4.4 to 6.9.4.5**

Description of change:

- Please insert new section "Pregnancy Reporting" after section 6.9.4.3

  to read:

  "Pregnancies must be notified by the investigator to the Glaxo Wellcome monitor within two weeks of the investigator learning of the pregnancy.

  Glaxo Wellcome will provide a Pregnancy Notification Form to collect the required data. Women who become pregnant must discontinue from the study."

Applies to:

Section 6.9.4.4 "Pregnancy Reporting"

Reason for change:

To reflect recent change in the GW standard requirements of pregnancy reporting.

CHANGE NUMBER 10:

10. **Amend Section 6.9.5 and insert a new section: "6.9.5.5 Handling of Rash/Allergic Reaction Episodes"**

Description of change:

- Please insert new section "6.9.5.5. Handling of Rash/Allergic Reaction Episodes"

  to read:

"Rash associated with systemic symptoms (eg. fever, nausea, vomiting) has been reported in approximately 4% of patients treated with 1592U89 which recurs within a few hours of rechallenge. Subjects who develop a rash in association with systemic symptoms should have all study medications interrupted. Subjects must not be rechallenged with 1592U89 without prior discussion with the sponsor. When the symptoms have resolved, subjects may be rechallenged with background antiretrovirals at the discretion of the investigator."

Applies to:

Section 6.9.5.5 "Handling of Rash/Allergic Reaction Episodes"

Reason for change:

To alert participating physicians about this side effect and to prevent subjects from experiencing a repetition of the event.

CHANGE NUMBER 11:

11. Amend Section 6.13. to include the validation of "positive" ADC end-points

Description of change:

- Section 6.13 change first two paragraphs from:

"For the purpose of this protocol, a dementia end-point is defined as progression by at least one stage within the MSK scale for ADC. At the time the subject is considered to have met the end-point, an exclusion diagnosis, confirmed by MRI Scan, CSF analysis and any other supporting documentation will be required."

Given the subjectivity involved in the assessment and staging of ADC, an end-point committee comprised by four participating investigators (see attached appendix K), has been established to independently review all dementia end-points occurred during the study."

to read:

"For the purpose of this protocol, a **negative** dementia end-point is defined as progression by at least one stage within the MSK scale for ADC. At the time the subject is considered to have met a **negative** end-point, an exclusion diagnosis, confirmed by MRI Scan, CSF analysis and any other supporting documentation will be required.

Given the subjectivity involved in the assessment and staging of ADC, an end-point committee comprised by four participating investigators (see attached appendix K), has been established to independently review all dementia end-points occurred during the study. **In addition to the validation of negative end-points, improvement by at least one stage within the MSK scale will also require validation by the end-point committee. For the validation of improvement, a clinical rationale, which will include a brief description of the improvement observed by the physician, caregiver and/or participating subject, will be submitted to the end-point committee.** To ensure an independent..."

Applies to:

Section 6.13 "Validation of Dementia End-Points"

Reason for change:

- Positive ADC end-points will be described as part of the efficacy assessment of 1592U89, therefore they merit the same validation scrutiny as the "negative" end-points.

CHANGE NUMBER 12:

12. **Append Section 9. EXTENSION PHASE to allow continuation of the study CNAB3001**

Description of change:

- Append Section 9 **to read:**

9. EXTENSION PHASE

9.1 Objectives

1) To assess long term safety and tolerance of 1592U89 in subjects with AIDS Dementia Complex

2) To evaluate survival

9.2 Rationale

Safety updates on the use of 1592U89 in clinical trials is regularly reported to the FDA. These data suggest that 1592U89 is generally well tolerated with an acceptable adverse experience and laboratory toxicity profile.

GW is committed to provide access to 1592U89 treatment to those subjects who have come to the end of the CNAB3001 study and continue to benefit from 1592U89. Extended therapy beyond 52 weeks is therefore warranted.

9.3 Study Design

This amendment provides a means to prolong access to 1592U89 treatment to those study participants who continue to benefit from 1592U89. Enrolment into this extension phase will only be offered to those subjects who successfully complete 52 weeks in the CNAB3001 study. Prior to enrolment patients will provide informed consent incorporating the provisions of this treatment extension phase. This study is extended until 1592U82 becomes commercially available. Subjects will be monitored every 12 weeks for safety and limited efficacy evaluations. Neurological assessments will be performed whenever a subject's clinical dementia status is suspected to have deteriorated. End-point validation will not be required in this part of the study.

9.4 Subject Selection

9.4.1 Inclusion Criteria

A. Subjects must have participated in study CNAB3001.

B. Written informed consent which incorporates this protocol amendment.

C. Subjects must be considered by the investigator to derive benefit from continued therapy with 1592U89.

9.4.2 Exclusion Criteria

A. Any Subjects that permanently discontinued 1592U89 therapy during the original 52 weeks of this protocol are excluded.

9.5 Concomitant Medications

There are no restrictions on the use of concomitant medication in this part of the study, however, attention is drawn to those compounds listed in section 5.5 to minimise the risk of any drug interaction.

9.6 Clinical Trial Material

1592U89 will be supplied as described in Section 5.1. Study drug will be shipped to study centres by Glaxo Wellcome. Accurate drug accountability records will be maintained by study personnel.

9.6.1 Dose & Regimens

Subjects will continue to receive 1592U89 600mg twice a day (approximately every 12 hours). Toxicity management guidelines are described in section 6.9.5. These guidelines are to be used at the discretion of the investigator.

9.7 Measurements & Evaluations

9.7.1 On Study

Laboratory Evaluations

Haematology (Weeks 64, 76, 88 , 100 and 12 weekly thereafter)
Serum Chemistries (Weeks 64, 76, 88 , 100 and 12 weekly thereafter)
Lymphocyte Subset (Weeks 64, 76, 88, 100 and 12 weekly

thereafter)
Plasma storage for virology analysis (Weeks 64, 76, 88, 100 100 and 12 weekly thereafter)
CSF storage for virology/inflammatory markers (optional)

Clinical Evaluations

ADC Rating (MSK scale) (Weeks 64, 76, 88 , 100 and 12 weekly thereafter)
Neurological assessment (as required)
HIV Associated Conditions (as required)
Adverse Event reporting (as required)
Study Drug Recording
Concomitant Medication (Summarised data)

9.7.2 Study Discontinuation

Laboratory Evaluations

Haematology
Serum Chemistries
Lymphocyte Subset
Plasma storage for virology analysis
CSF storage for virology/inflammatory markers (optional)

A Phase III, Randomised, Double-Blind, Multicentre Study to Evaluate the Safety and Efficacy of 1592U89 in HIV-1 Infected Patients with AIDS Dementia Complex - Incorporating Amendment Number 4
Protocol Number CNAB3001

PROTOCOL AMENDMENT 4 - Date 28 August 1997

Clinical Evaluations

ADC Rating (MSK scale)
Neurological assessment
HIV Associated Conditions
Adverse Event reporting
Karnofsky Score
Study Drug Recording
Concomitant Medication

9.7.3 Follow-up (Off-drug)

A Follow-up evaluation should be scheduled at approximately 30 days following discontinuation of therapy with 1592U89

Laboratory Parameters

Haematology
Serum Chemistries
Lymphocyte Subset
Plasma storage for virology analysis
CSF storage for virology/inflamatory markers (optional)

Clinical Evaluations

ADC Rating (MSK scale)
Adverse Event reporting

Applies to:

Section 9

Reason for change:

To allow access to 1592U89 treatment to subjects beyond 52 weeks

A Phase III, Randomised, Double-Blind, Multicentre Study to Evaluate the Safety and Efficacy of 1592U89 in HIV-1 Infected Patients with AIDS Dementia Complex - Incorporating Amendment Number 4
Protocol Number CNAB3001

PROTOCOL AMENDMENT 4 - Date 28 August 1997

CHANGE NUMBER 13:

13. Insert Appendix A1 (Assessment Timeline for the Extension Phase) after Appendix A

Description of change:

- Insert Appendix A1 (Assessment Timeline for the Extension Phase) immediately after Appendix A

  to read:

  Please refer to Appendix A1

Applies to:

Appendix A1

Reason for change:

To describe the evaluation schedule of the Extension Phase

CHANGE NUMBER 14:

14. Amend Appendix K to reflect change No.9 from this amendment

Description of change:

- Appendix K, Objectives section, objective No.2, delete "Study Treatment Failure and"

  to read:

  "... 2. To endorse any premature commencement of the study's open phase."

Applies to:

Appendix K, "Operating Principles of End-Point Committee"

Reason for change:

The objective of endorsing "Study Treatment Failure or Success" is already covered by objective 1.

CHANGE NUMBER 15:

15. Amend Appendix J1 to modify the toxicity grading values for triglycerides

Description of change:

- Appendix J1, Laboratory Test Abnormalities, modify grading values for triglycerides from:

  Grade 1: >1.0-1.5 X Upper Normal Limit
  Grade 2: >1.5-2.0 X Upper Normal Limit
  Grade 3: >2.0-5.0 X Upper Normal Limit
  Grade 4: >5.0 X Upper Normal Limit

  to read:

  Grade 1: ULN-399 mg/dL or ULN-4.50 mmol/L
  Grade 2: 400-750 mg/dL or 4.51-8.50 mmol/L
  Grade 3: 751-1200 mg/dL or 8.51-13.50 mmol/L
  Grade 4: >1200 mg/dL or >13.50 mmol/L

Applies to:

Appendix J1, "Laboratory Test Abnormalities"

Reason for change:

Hypertriglyceridemia has been documented in patients with advanced HIV infection and has also been seen in patients receiving antiretroviral therapy with protease inhibitors. The magnitude and frequency of triglyceride elevation associated with antiretroviral therapy is currently uncertain.

Hypertriglyceridemia is a risk factor for the development of pancreatitis and triglyceride levels are monitored in all patients enrolled in clinical trials. A number of toxicity scales are currently in use. Since pancreatitis is unusual at triglyceride levels below 1000 mg/dL the above scale will be used in all Glaxo Wellcome protocols evaluating anti-HIV compounds.
