# Supplementary material for: Factors in AIDS Dementia Complex Trial Design: Results and Lessons from the Abacavir Trial
Source: PLoS Clin Trials. 2007 Mar 30;2(3):e13. doi: 10.1371/journal.pctr.0020013 (PMC1845158; doi:10.1371/journal.pctr.0020013)
Supplement: Text S1 Clinical Evaluation Form [file pctr.0020013.sd003.pdf]

Patient #: [- -] Exam (T) # [.]

NEUROLOGICAL HISTORY

(Patient and Companion Complaints Elicited)

COGNITIVE FUNCTION (Elicited Complaints)

a. Concentration/Speed of Thought.. [ ]

0 - normal mental agility; 1 - notes some diminished capacity for concentration or need for longer time to accomplish normal tasks, but able to manage nearly all of daily affairs; 2 - definitely loses track of conversation or task, takes more than twice as long to complete some tasks, needs help to manage more difficult tasks of daily life (eg financial records) or complex work activities; 3 - marked slowing of mental processes, perseveration or loss of thought train, needs help to manage almost all daily affairs requiring cognition; 4 - only rudimentary cognition; 8 - cannot evaluate or not applicable; 9 - not elicited.

b. Reading (or TV).. [ ]

0 - normal; 1 - notes mild increase in effort or occasionally loses place in either reading or TV; 2 - reduced reading or TV because of slowness, clearly more laborious; or requires repeated efforts, but can follow news and similar subjects; 3 - reading or TV is markedly reduced because of difficulty attending or following plot, but occasionally reads or watches with reduced attention or comprehension; 4 - unable to read or watch TV at all because cannot make sense of subject, etc.; 8 - cannot evaluate or not applicable; 9 - not elicited.

c. Memory.. [ ]

0 - normal; 1 - mildly more forgetful than usual, will occasionally miss appointments; 2 - definite memory difficulty, needs to keep lists, forgets events of day or activities in mid-task; 3 - frankly confused at times about events, places, person; 4 - persistently disoriented to time, frequently to place or person; 8 - cannot evaluate or not applicable; 9 - not elicited.

d. Speech.. [ ]

0 - normal; 1 - mild hesitancy, extra deliberation, or occasional use of inappropriate words; 2 - definite slowness or difficulty finding words or use of wrong or inappropriate words; 3 - reduced speech output or frequent speech errors; 4 - speech limited to few phrases at best; 8 - cannot evaluate or not applicable; 9 - not elicited.

e. ADC COGNITIVE SYMPTOM INDEX.. [|]

(Sum a-d; invalid if any response 8 or 9, score as 99)

f. Date of Onset of ADC-Related Cognitive Symptoms........................ [/ /]

MOTOR/COORDINATION (Elicited Complaints)

a. Gait.. [ ]

0 - normal; 1 - mild unsteadiness, imbalance, slowness or weakness, but fully ambulatory without assistance; 2 - unsteady or weak, requiring cane or some prop; 3 - requires bilateral (e.g. walker or human) help; 4 - unable to walk; 8 - cannot evaluate or not applicable; 9 - not elicited.

b. Upper Extremity Coordination.. [ ]

0 - normal dexterity; 1 - mild slowing or clumsiness of hands without functional impairment; 2 - moderate clumsiness of hands, altering handwriting or slowing regular hand activities (eating, etc.); 3 - marked clumsiness resulting in difficulty performing ADL (e.g. difficulty feeding without manual help of others); 4 - unable to perform hand- related ADL because of incoordination; 8 - cannot evaluate or not applicable; 9 - not elicited.

c. Involuntary Movement (includes tremor, myoclonus & chorea)........................ [ ]

0 - none or normal mild tremor; 1 - noticeable new or increase in adventitious movements, but doesn't interfere with ADL; 2 - new or increased adventitious movements impair handwriting or other manual tasks, but still can function independently for most ADL; 3 - marked adventitious movements interferes with most ADL; 4 - severe adventitious movements necessitate nearly full-time care; 8 - cannot evaluate or not applicable; 9 - not elicited.

d. Bladder Incontinence ....

0 - nl control; 1 - rare or occasional loss (doesn't make it) or hesitancy; 2 - incontinence > 1/day; 3 - requires indwelling Foley or Texas; 8 - cannot evaluate or not applicable; 9 - not elicited.

e. ADC MOTOR SYMPTOM INDEX ....

(Sum a-d; invalid if any response 8 or 9, score as 99)

f. Date of Onset of ADC-Related Motor Symptoms .... / /

BEHAVIOR/MOOD (Elicited Complaints - patient & others)

a. Mood ....

0 - normal spirits, no depression; 1 - depressed about illness but doesn't intrude on function; 2 - frequently feels depressed, interferes with functioning; 3 - very depressed, requiring medication or regular therapy; 4 - severe depression, requires suicide precautions; 8 - cannot evaluate or not applicable; 9 - not elicited.

b. Social apathy/withdrawal ....

0 - normal socialization; 1 - diminished interest but continues with normal activities; 2 - activities reduced because of lack of interest or initiative but maintains most contacts; 3 - marked loss of interest in friends and normal social activities, doesn't seek social contact; 4 - little or no meaningful socialization; 8 - cannot evaluate or not applicable; 9 - not elicited.

c. Emotional lability/agitation ....

0 - normal; 1 - more easily agitated or excited, but doesn't alter social ability; 2 - clearly labile or hyperactive so that interactions are altered (inappropriate insults, hypomanic behavior); 3 - maintained in a state of constant hyperactivity or agitation, may experience hallucinations; 4 - frank delirium; 8 - Cannot evaluate or not applicable; 9 - not elicited.

d. ADC BEHAVIOR SYMPTOM INDEX ....

(Sum a-c; invalid if any response 8 or 9, score as 99)

e. Date of Onset of ADC-Related Behavioral Symptoms .... / /

OVERALL ADC SYMPTOM INDEX ....

(Sum Cognitive, Motor & Behavioral, but omit Mood; invalid of any entries 8 or 9, score as 99)

HEADACHE ....

0 - none or occ. as before illness; 1 - mild, occasional (approximately 1/week); 2 - moderate (nearly daily, requiring repeated nonnarcotic analgesics; 3 - severe (daily, requiring narcotic analgesics); 8 - cannot evaluate or not applicable; 9 - not elicited.

ALERTNESS/SLEEP

a. Decreased Alertness ....

0 - normal diurnal alertness; 1 - tendency to fall asleep, sleeps somewhat more than normal pattern; 2 - frequently falls asleep, but still easily arousable; 3 - sleeps much of the day, may be more difficult to arouse; 4 - clear impairment of consciousness with lethargy or coma; 8 - cannot evaluate or not applicable; 9 - not elicited.

b. Insomnia ....

0 - normal or increased nocturnal sleep; 1 - mild insomnia, may require occ. hypnotic; 2 - moderate-severe insomnia, up much of night or requires nightly hypnotic; 8 - cannot evaluate or not applicable; 9 - not elicited.

SEIZURES (within last 4 weeks) ....

0 - none; 1 - single partial seizure; 2 - repeated partial or grand mal seizures; 3 - frequent (> 4/wk) partial or generalized seizures; 8 - cannot evaluate or not applicable; 9 - not elicited.

SENSATION

a. Paresthesias (tingling or numbness) or pain due to neuropathy.................... 2
0 - none; 1 - mild distal sensations; 2 - moderate distal sensations to ankles or wrists; 3 - severe disturbing sensations interfering with normal functioning; 8 - cannot evaluate or not applicable; 9 - not elicited.

b. Loss..
0 - none; 1 - mild distal decrease in sensation; 2 - moderate distal loss to ankles or wrists, interferes with normal activities; 3 - severe loss such that interferes with ADL; 8 - cannot evaluate or not applicable; 9 - not elicited.

Date of Onset of HIV-Related Neuritic Symptoms.......................... / /

COMMENT:

NEUROLOGICAL EXAMINATION

LEVEL OF CONSCIOUSNESS (best fit)...
0 - normal; 1 - delirious; 2 - agitated; 3 - anxious; 4 - slow; 5 - lethargic but readily arousable; 6 - lethargic, requiring frequent arousal; 7 - stuporous, unresponsive to verbal stimuli; 8 - persistent vegetative state or coma; 9 - did not assess.

COMMENT

MENTAL STATUS

a. Time: year, season, date, day, month (1-5; score 1 for each correct)................

b. Place: state, county, town, hospital, floor (1-5; score 1 for each correct).........

c. Registration...
Name 3 objects: a color, a fruit and a city, with 1 second to say each. Then ask patient all 3 after you have said them. (1-3; score 1 for each correct)

d. Attention and Concentration...
Spell "world", "horse", "black", "catch" or "bread" backwards.
(1-5; score number of letters correct before first error)

Language:

e. Name a pencil, and watch (2)..

f. Repeat the following, "No ifs, ands or buts". (1)........................

g. Follow a 3-stage command..
"Touch your left ear with your right thumb and stick out your tongue."
(1-3; score 1 for each correct action; left ear, right thumb, tongue)

h. Recall...
Ask for 3 objects repeated above. (1-3; score 1 point for each correct)

i. Read and obey the following: "Close your eyes." (1)..........................

j. Write a sentence: "Today is a nice day." (1) ....

Patient #: [- -] Exam (T) # [.]

k. Copy a design. (1) .... [ ]

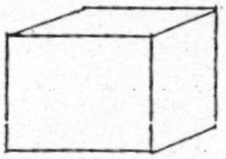

TOTAL SCORE OF MENTAL STATUS (sum a-k; possible 30) .... [|]

RESPONSE SLOWING .... [ ]
0 - normal or hyperactive; 1 - mild slowing of response time;
2 - moderate slowing of response time; 3 - severely slowed, nearly or fully mute;
8 - could not assess; 9 - did not assess.

AFFECT:

a. Degree of Alteration .... [ ]
0 - normal emotional responses & expression; 1 - mildly abnormal affect;
clearly inappropriate but allows coherent & full interaction; 2 - moderate-to-severely altered affect, heavily colors all interactions; 8 - could not assess;
9 - did not assess.

b. Type of Alteration .... [ ]
0 - normal; 1 - labile, increased emotionality; 2 - prevalent anxiety,
hyperactivity; 3 - hypomania or mania; 4 - giddy, childlike, inappropriately
light; 5 - depressed; 6 - flat, lacking emotional expression; 7 - other;
8 - could not assess; 9 - did not assess.

Comment ____

CRANIAL NERVES

II:

a. retina .... [ ]
0 - normal; 1 - cotton wool (AIDS) retinopathy; 2 - hemorrhagic (presumed CMV retinopathy); 3 - other abnormality; 8 - could not assess; 9 - not tested

b. Mydriatic used .... [ ]
0 - no; 1 - yes; 9 - not tested.

c. Fields .... [ ]
0 - normal; 1 - abnormal; 8 - could not assess; 9 - not tested

d. Acuity .... [ ]
0 - normal; 1 - abnormal; 8 - could not assess; 9 - not tested

Comment ____

Ocular motility

e. Smooth Pursuits .... [ ]
0 - normal; 1 - mildly abnormal; 2 - moderately-severely abnormal;
8 - could not assess; 9 - not tested.

f. Saccades .... [ ]
0 - normal; 1 - mildly abnormal; 2 - moderately-severely abnormal;
8 - could not assess; 9 - not tested.

g. Muscle Weakness (strabismus) .... [ ]
0 - none; 1 - abnormal; 8 - could not assess; 9 - not tested.

h. Nystagmus .... [ ]
0 - none (or gaze paretic); 1 - fine horizontal nystagmus; 2 - coarse, vertical or directional; 8 - could not assess; 9 - not tested.

i. Possibly related to medication .... [ ]
0 - no; 1 - yes; 9 - not tested.

Comment ____

Patient #: [- -] Exam (T) # [.]

j. Facial Expression.. [ ]
0 - normal; 1 - mild hypomotility; 2 - moderate-severe loss of expression, including frank mask-like facies; 8 - could not assess; 9 - not tested.
Comment ______________________________

k. Dysarthria.. [ ]
0 - normal; 1 - mild slurring or slowing of speech, readily understood; 2 - moderate to severe slurring or slowing of speech, enough to impair understanding at times; 8 - could not assess; 9 - not tested.

MOTOR

Strength (worst muscle group or groups)

a. R Leg.. [ ]
0 - normal; 1 - mild weakness (4/5); 2 - moderate weakness (3/5); 3 - severe weakness (2/5 or worse); 8 - could not assess; 9 - not tested.

b. L Leg.. [ ]
0 - normal; 1 - mild weakness (4/5); 2 - moderate weakness (3/5); 3 - severe weakness (2/5 or worse); 8 - could not assess; 9 - not tested.

c. R arm.. [ ]
0 - normal; 1 - mild weakness (4/5); 2 - moderate weakness (3/5); 3 - severe weakness (2/5 or worse); 8 - could not assess; 9 - not tested.

d. L arm.. [ ]
0 - normal; 1 - mild weakness (4/5); 2 - moderate weakness (3/5); 3 - severe weakness (2/5 or worse); 8 - could not assess; 9 - not tested.

e. Tone.. [ ]
0 - normal or decreased tone; 1 - mild hypertonia; 2 - moderate-severe hypertonia; 8 - could not assess; 9 - not tested.
Comment ______________________________

COORDINATION

a. Gait coordination.. [ ]
0 - normal gait; 1 - mild impairment (evident only on rapid turns or tandem); 2 - moderate impairment (clear difficulty of unassisted gait); 3 - severe impairment (walking only with assistance); 4 - non-ambulatory; 7 - weakness precludes assessment of gait coordination; 8 - could not assess; 9 - not tested.

b. Type of gait disorder (predominant).. [ ]
0 - normal (default); 1 - cerebellar ataxia; 2 - spastic; 3 - spastic & ataxic; 4 - "extrapyramidal"; 5 - paraparetic; 6 - other; 8 - could not assess; 9 - not tested.
Comment ______________________________

c. Limb coordination.. [ ]
(test rapid finger-tapping and successive movements, wrist rotation, finger-nose-finger, foot-tapping, and heel to knee-shin-ankle maneuvers)
0 - normal; 1 - mild slowness or clumsiness (compared to examiner, the movement is slightly slower, fatigues or breaks down earlier); 2 - moderate slowness or clumsiness (notably slowed or dysmetric, readily appreciated even at start of task); 3 - severe slowness or clumsiness; 7 - weakness precludes testing; 8 - could not assess; 9 - not tested.

d. Pattern of limbs affected.. [ ]
0 - normal (default); 1 - legs predominate; 2 - ipsilateral arm & leg; 3 - one arm; 4 - one leg; 5 - all 4 extremities; 6 - arms predominate; 7 - other; 8 - could not assess; 9 - not tested.
Comment ______________________________

Patient #: [- -] Exam (T) # [.]

ADVENTITIOUS MOVEMENTS

a. Tremor (essential, elicited in outstretched hands).................................. [ ]
0 - none; 1 - mild; 2 - moderate; 3 - severe; 8 - could not assess;
9 - not tested.

b. Myoclonus.. [ ]
0 - none; 1 - mild; 2 - moderate; 3 - severe; 8 - could not assess;
9 - not tested.

c. Chorea... [ ]
0 - none; 1 - mild; 2 - moderate; 3 - severe; 8 - could not assess;
9 - not tested.

d. Resting tremor (Parkinsonian)... [ ]
0 - none; 1 - mild; 2 - moderate; 3 - severe; 8 - could not assess;
9 - not tested.

Comment ____

REFLEXES

a. Deep Tendon Reflexes... [ ]
0 - normal; 1 - increased in legs only; 2 - generalized increase; 3 - general decrease (most notably distally); 4 - increased proximally & depressed distally; 5 - increased on one side; 6 - other; 8 - could not assess; 9 - not tested.

b. Jaw jerk... [ ]
0 - absent; 1 - present; 2 - unusually brisk (2-3+); 8 - could not assess;
9 - not tested.

c. Glabellar reflex (Myerson's sign).. [ ]
0 - absent; 1 - present; 2 - prominent; 8 - could not assess;
9 - not tested.

d. Grasp reflex... [ ]
0 - absent; 1 - present; 2 - prominent; 8 - could not assess;
9 - not tested.

e. Snout reflex... [ ]
0 - absent; 1 - present; 2 - prominent; 8 - could not assess;
9 - not tested.

Comment ____

SENSATION

a. Loss due to neuropathy... [ ]
0 - normal; 1 - mild impairment (eg, increased threshold to position, vibration in great toes); 2 - moderate impairment (eg, decrease in position or vibration, pin or cold to ankles, or consistent similar impairment on one side of body); 3 - severe impairment (eg, decrease or loss of sensation to knees or wrists or comparable level); 8 - could not assess; 9 - not tested.

b. Modality... [ ]
0 - normal (default); 1 - predominantly pin or temperature;
2 - predominantly position or vibration sense; 3 - mixed;
8 - could not assess; 9 - not tested.

c. Pattern of sensory loss.. [ ]
0 - normal (default); 1 - distal symmetrical; 2 - hemisomatic;
3 - monoradiculopathy or mononeuropathy; 4 - polyradiculopathy or mononeuritis multiplex; 5 - sensory level (transverse myelopathy; specify);
8 - could not assess; 9 - not tested.

d. Hyperesthesia.. [ ]
0 - None; 1 - mild hyperesthesia; 2 - moderate-to-severe hyperesthesia;
8 - could not assess; 9 - not tested.

Comment ____

Patient #: [- -] Exam (T) # [.]

SUMMARY ASSESSMENTS:

a. Overall Opinion of Dementia.. [ ]

0 - normal; 1 - mild dementia (symptoms or mild signs, but not functional impairment in ADL); 2 - moderate dementia (functionally impaired so that cannot do work or demanding tasks of ADL, but can simply interact socially); 3 - severe dementia with very limited intellectual or social interaction; 8 - cannot judge; 9 - not assessed.

Comment ______________________________

b. Overall Behavioral Disturbance.. [ ]

0 - none; 1 - mild symptoms or signs but able to function socially normally or close to normally; 2 - moderate symptoms & signs, interfers with normal social interaction or capacity to work; 3 - severe symptoms & signs requiring nearly constant attention or institutionalization; 8 - cannot judge; 9 - not assessed.

Comment ______________________________

c. Overall CNS Motor.. [ ]

0 - none; 1 - mild symptoms or signs but independent ambulation and ADL; 2 - moderate symptoms & signs, needs prompts or assistance with walking or ADL; 3 - severe symptoms & signs, nonambulatory or can only walk few feet with full assistance; 8 - cannot judge; 9 - not assessed.

Comment ______________________________

d. Overall Peripheral Neuropathy

Severity of neuropathy.. [ ]

0 - none; 1 - mild weakness or sensory impairment but independent ambulation and hand use; 2 - moderate, needs assistance to ambulate or with hand tasks; 3 - severe, unable to ambulate or do hand tasks; 8 - cannot judge; 9 - not assessed

Type of neuropathy.. [ ]

0 - default
1 - predominantly distal sensory
2 - predominantly motor
3 - mixed distal
4 - mononeuritis localized radiculopathy
5 - mononeuritis multiplex or polyradiculopathy
6 - other
8 - cannot judge
9 - not assessed

Comment ______________________________

NEUROLOGY SUBSCORES		
SOURCE	ITEMS	SCORE
NEUROLOGICAL SYMPTOMS: Cognitive Function	Combined score of:<br>a. Concentration/speed of thought (0-4)<br>b. Reading/TV (0-4)<br>c. Memory (0-4)<br>d. Speech (0-4)	If (a+b+c+d)≥2, but ≤5, score=1<br>If (a+b+c+d)≥6, but ≤8, score=2<br>If (a+b+c+d)≥8, score=3
NEUROLOGICAL SYMPTOMS: Motor Function	Combined score of:<br>a. Gait (0-4)<br>b. Upper extremity coordination (0-4)<br>c. Involuntary movement (0-4)<br>d. Bladder incontinence (0-4)	If (a+b+c+d)≥1, but ≤4, score=1<br>If (a+b+c+d)≥5, but ≤7, score=2<br>If (a+b+c+d)≥8, score=3
NEUROLOGICAL EXAMINATION: Mental Status	Combined score of:<br>a. Modified Mini Mental Status:<br>25-23, score=0<br>22-20, score=1<br>≤20, score=2<br>b. Response slowing (0-3)	If (a+b)=2, score=1<br>If (a+b)≥3, score=2
MOTOR FUNCTION: Ocular Motility	Combined score of:<br>a. Smooth pursuits (0-2)<br>b. Saccades (0-2)	If (a+b)≥1, score=1<br>If (a+b)≥3, score=2
MOTOR FUNCTION: Coordination	Combined score of:<br>a. Gait coordination (0-4)<br>b. Limb coordination (0-2)	If (a+b)=1, score=1<br>If (a+b)≥2, score=2<br>If (a+b)≥4, score=3
MOTOR FUNCTION: Reflexes	Combined score of:<br>a. Jaw jerk (0-2)<br>b. Snout (0-2)	If (a+b)=2, score=1<br>If (a+b)≥3, score=2
NEUROLOGY SUBSCORE TOTAL		
